# Bayesian Optimization of Catalysis with In-Context Learning

**DOI:** 10.1021/acscentsci.5c02418

**Published:** 2026-04-14

**Authors:** Mayk Caldas Ramos, Shane S. Michtavy, Andrew D. White, Marc D. Porosoff

**Affiliations:** † Department of Chemical and Sustainability Engineering, University of Rochester, Rochester, New York 14627, United States; ‡ Edison Scientific Inc., San Francisco, California 94107, United States

## Abstract

Large language models (LLMs) can perform accurate classification with zero or few examples through in-context learning (ICL), allowing the model to observe query-relevant examples at inference time and eliminating the need for additional weight updates to generalize beyond its original training data. We extend this capability to regression with uncertainty estimation using frozen LLMs (e.g., GPT-4o, Gemini), enabling Bayesian optimization (BO) in natural language without explicit model training or feature engineering. We apply this to materials discovery by representing materials as synthesis and testing procedures for use in natural language prompts. This Bayesian, design-first approach prioritizes optimization toward target material properties before detailed characterization, in contrast to conventional experimental workflows that often emphasize characterization of suboptimal materials. On benchmarks like aqueous solubility and oxidative coupling of methane (OCM), BO-ICL matches or outperforms Gaussian processes. In live experiments on the reverse water–gas shift (RWGS) reaction, BO-ICL identifies multimetallic catalysts that approach equilibrium CO yield within 6 and 10 iterations from a pool of 3,700 and 360,000 candidates, respectively. Our method redefines materials representation and accelerates discovery, with broad applications across catalysis, materials science, and AI. Code: https://github.com/ur-whitelab/BO-ICL.

## Graphical abstract

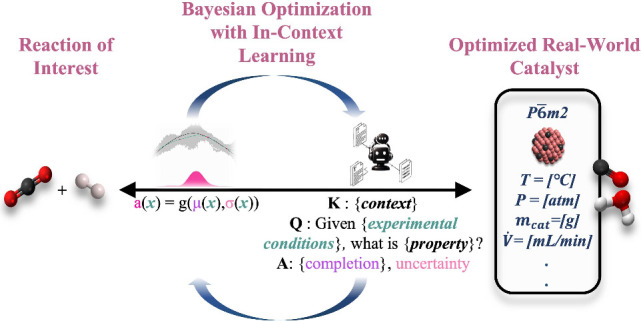

## 1 Introduction

Transformer-based large language models (LLMs) leverage large-scale pretraining to learn representations that transfer across tasks, reducing the need for task-specific engineering.^1–9^ Beyond natural-language tasks, LLMs (and closely related transformer models) are applied to medicine,^10–14^ material property predictions,^15–20^ and molecular design.^21–26^ A distinctive capability of modern pretrained LLMs is in-context learning (ICL), where performance on a task can improve when the model is conditioned on a small number of query relevant examples in the prompt.^27^ Here, we investigate whether ICL can be coupled with sequential optimization to design new materials.

Bayesian optimization (BO) is a common technique for sample-efficient black-box optimization, including constrained settings.^28^ BO addresses problems of the form

1 $$\arg\underset{x\in \Omega }{\max}f\left(x\right)$$

i.e., finding an input *x* in the parameter space Ω that maximizes the objective function *f* (eq 1). BO uses predictions and uncertainty estimates from probabilistic models to balance exploration and exploitation when selecting query points.^29–32^ More specifically, BO performs gradient-free optimization of a black-box function *f*(*x*) by employing a surrogate model S(x) to approximate *f*(*x*) and an acquisition function α­(*x*) to select the next evaluation point. After each evaluation, the observed data are used to update S(x) , and α­(*x*) is recomputed to guide subsequent queries.^33^ A detailed description of BO is available in Section 5.1.

A common choice for a surrogate function is a Gaussian process (GP) model; GPs are flexible nonparametric models that yield posterior predictive distributions and principled uncertainty estimates.^33,34^ In this work, we instead use an LLM as the surrogate model. Pretrained LLMs provide rich transfer-learning priors that improve generalization and can enable faster convergence in low-data regimes. We leverage the model’s conditional predictive distribution to obtain an uncertainty signal (e.g., via the entropy or dispersion of its output distribution). Through ICL, the LLM surrogate can be updated at inference by appending newly observed (*x*, *f*(*x*)) pairs to the prompt. This allows rapid sequential updates without costly retraining required by conventional surrogates. Together, these properties motivate LLM-based surrogates for fast, iterative decision-making in BO.

Using an LLM as a surrogate model enables the use of natural language as a feature vectors. This is particularly valuable for domain applications that are challenging to model, such as experimental protocols that represent a catalyst.^35–38^ Natural language provides a straightforward way to integrate both relevant qualitative and quantitative information into representations, which can then be optimized. Building on this capability, Jablonka et al.^18^ demonstrated that decoder-only models, like the generative pretrained transformer (GPT) can predict material and chemical properties using Language-Interfaced Fine-Tuning (LIFT).^27,39^ LIFT converts tabular data into sentences and then fine-tunes an LLM using the resulting natural language representation (illustration in Section S1 and Figure S1).

The application of LIFT using GPT models has succeeded in tasks such as classification, regression, and inverse design, without requiring modifications to model architectures or training procedures.^39,40^ However, using GPT models as surrogates for BO introduces additional challenges, such as the requirement of substantially more training compute. Surrogate models are updated upon each observation in BO, which is a significant additional burden on LLM training in the LIFT paradigm.^41^

Fortunately, there are alternative strategies to retraining the LLM upon a BO update, such as ICL.^42^ ICL enhances performance by allowing the model to observe query-relevant examples at inference time,^27^ eliminating the need for additional weight updates to generalize beyond its original training data.^43,44^ Recent research highlights success using similar ICL prompting techniques, such as chain-of-thought^45–47^ and the use of symbolic tools (e.g., programming languages) to improve accuracy.^48,49^ Thus, ICL enables models to improve prediction accuracy even when new data are available at a limited rate, a useful attribute for a BO workflow.

The integration of pretrained LLMs with BO has become an active area of research after our early demonstrations of their potential.^14,50,51^ Notably, Kristiadi et al.^52^ shows that domain-specific LLMs, trained via parameter-efficient fine-tuning (PEFT), achieve success in simpler BO settings. With inspiration from these prior ideas, we present a novel approach that successfully leverages LLMs as surrogate models in a BO policy via an ICL. Figure 1 shows a high-level illustration of our method of integrating BO with the ICL, and further details are available in Section 5. Our process introduces an AskTell algorithm that utilizes ICL as the primary mechanism for updating the surrogate LLM’s knowledge during the BO process (Sections 5 and S2).

**Figure 1:**
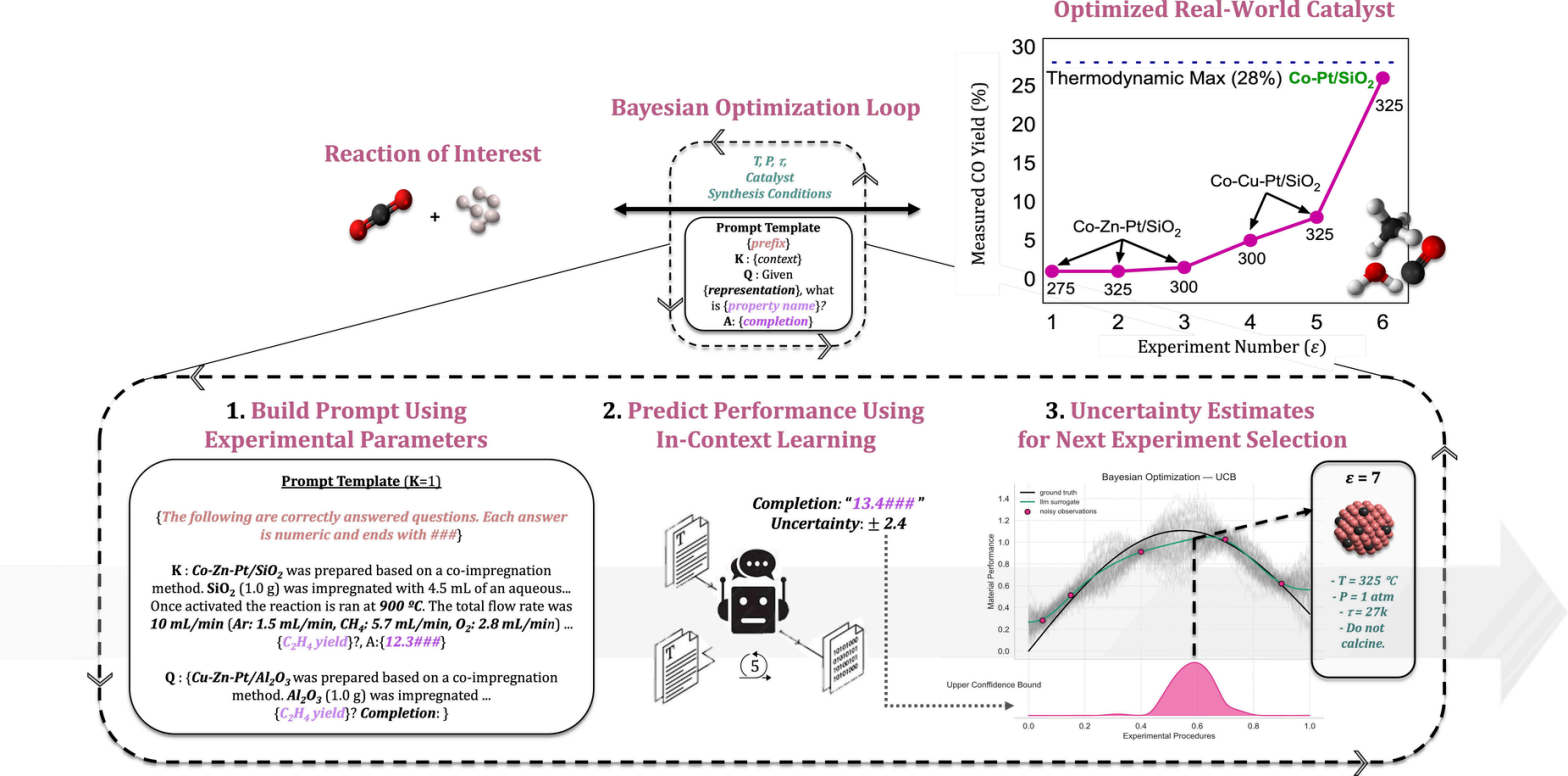
A high-level overview of our closed-loop Bayesian optimization (BO) method that uses natural language to represent a material design space for efficient sample exploration. The workflow involves conversion of tabular data into an experimental procedure, which incorporates both synthesis and reaction parameters. By formatting material parameters for compatibility with state-of-the-art large language models, this approach leverages well-established BO techniques to efficiently identify actionable experimental conditions that maximize a desired objective function. In this figure, we highlight a success case for optimizing catalysts for selective CO_2_ conversion to CO via BO-ICL.

AskTell means we first query the model for a point with an Ask, and then we respond to the model with a Tell step, reporting the outcome of the experiment. By dynamically constructing prompts with relevant context at inference time, we eliminate the need for resource intensive weight updates, as is common when updating a model as new data becomes available. This yields a task-agnostic, ready-to-use approach that operates directly with natural language.

To validate our workflow, we focus on the materials design for greenhouse gas (GHG) upcycling, an application area of global significance. Accelerating materials discovery in this domain can reduce reliance on crude oil for high-demand precursors such as carbon monoxide and olefins.^53^ By targeting heterogeneous catalytic reactions involving GHGs such as CO_2_, we may help mitigate GHG emissions, and in turn, the global temperature rise.^54,55^ Applying AI to enhancing materials design and discovery has the potential to impact each step in such a circular carbon economy by helping to offset the inherent entropic penalties associated with the capture and conversion of relevant GHGs.^55,56^

Given the vast design space of heterogeneous catalysts and the additional complexity of optimizing reaction conditions, catalysis offers a compelling use case for frozen LLMs as surrogate models within a BO framework.^56^ Language-based representations of materials allow experimentalists to optimize catalytic performance by formatting inputs, such as synthesis procedures and reaction conditions, in a structured and intuitive manner with property values as outputs (see Figure 1). Leveraging pretrained LLMs for prompt-level transfer learning is expected to improve optimization efficiency, reduce experimental overhead, and accelerate catalyst discovery.

In this work, we investigate whether ICL with state-of-the-art LLMs serves as an effective surrogate model within a BO framework. Our central hypothesis is that language-based representations contain sufficient structure and physical information to enable efficient experimental design, even without domain-specific feature engineering. We begin by evaluating scalability through two regression tasks: predicting molecular solubility from IUPAC names and catalytic performance in the oxidative coupling of methane (OCM) reaction using natural language descriptions of synthesis and reaction conditions (Section 2.1). We then assess BO-ICL’s sample efficiency on the OCM data set from Nguyen et al.^57^ and an alloy interface property data set from Gerber et al.^58^ (Sections 2.2.1 and 2.2.2, respectively), showing rapid convergence to the 1% top-performing candidates after labeling only 30 experiments. Finally, we apply BO-ICL to guide real-world on-the-fly experimental synthesis and testing for the reverse water gas-shift (RWGS) reaction using multimetallic catalysts, achieving near-thermodynamic equilibrium performance in less than ten iterations in two different data sets (Section 2.2.3). Together, these results support our goal of enabling general-purpose, language-native optimization workflows for broad applications in catalysis and materials design.

## 2 Results and Discussion

We use four use cases to evaluate the performance of our method: estimated solubility (ESOL),^59^ oxidative coupling of methane (OCM),^57^ modeled alloy interface interaction (AII),^58^ and in-house data sets generated for CO_2_ hydrogenation via RWGS. Detailed descriptions of these data sets are available in Section S1.

Initially, we employ ESOL and OCM data sets in a regression task to investigate how the performance of our ICL approach depends on key hyperparameters: the number of examples used in the prompt (*k*), the uncertainty scaling factor for calibration, and the temperature (*T*) (see Section 5.2 for application steps). These regression experiments (Section 2.1) confirm that the model learns directly from the natural language representations. To benchmark the performance of the LLM against other commonly used machine learning models, we test three baseline methods: *k*-nearest neighbor^60^ (KNN), kernel ridge regression^61,62^ (KRR), and Gaussian process regression^63^ (GPR). Implementation details for the baselines are provided in Section S3.

Next, in Section 2.2, we perform optimization using LLMs as surrogate models combined with ICL to iteratively update model knowledge using the OCM and AII data sets (retrieval-augumented generation (RAG) workflow illustration is in Section 5.2 and associated algorithms in Section S2). We observe that BO-ICL reaches the 99th percentile of active catalysts while requiring, on average, less than 30 iterations (Section 2.2).

Finally, we construct an unlabeled pool of potential experiments for in-house synthesis and testing, comprising experimental procedures for the RWGS reaction. We use BO-ICL to iteratively guide the selection of subsequent experiments with the CO yield as the objective function in the RWGS catalyst design space. We demonstrate that BO-ICL effectively selects catalyst formulations and experimental procedures that achieve CO yields closely approaching the thermodynamic limit (see Section 2.2.3). All results use an embedded natural language representation of the sampled experimental procedures as the input feature representation.

### 2.1 Regression

We begin our analysis by identifying key hyperparameter values and examining how the number of known examples stored in the model’s memory (available context) influences prediction performance using regression analysis (Section 5.3). Motivated by insights from this exploratory analysis, we conducted subsequent experiments using five context examples per prompt, a temperature setting of 0.7, and an uncertainty scaling factor of 5. Section 5.3 illustrates the impact of these hyperparameters on prediction performance for the ESOL and OCM data sets using the gpt-3.5-turbo-0125 and gpt-4o-2024–08–06 models.

To assess the performance of our ICL approach relative to those of more traditional methods, we benchmark against KRR, a fine-tuned variant of gpt-3.5-turbo-0125, and GPR. Figure 2 and Section S4.1 present results of the OCM and solubility data sets, respectively. The baselines demonstrate strong performance across data sets in comparison with the ICL approach, consistent with previous findings in the literature.^18^ Baseline model performance advantages likely arise from task specific parameter updates, in contrast with the continuous reuse of a single general-purpose LLM in the ICL setup. Specifically, the KRR likely benefits from its capacity to manage high-dimensional feature spaces through loss regularization. In the fine-tuned LLM case, it would be surprising for the ICL case to perform better since it uses the same models, with omission of the task specific training. Nevertheless, using ICL with general-purpose LLMs does not require any adaptation of the model or further training, proven to be a promising approach to quickly adapting off-the-shelf LLMs to domain-specific problems. The literature supports our hypothesis that the efficacy of ICL likely stems from a nearest-neighbor-like mechanism.^64,65^

**Figure 2:**
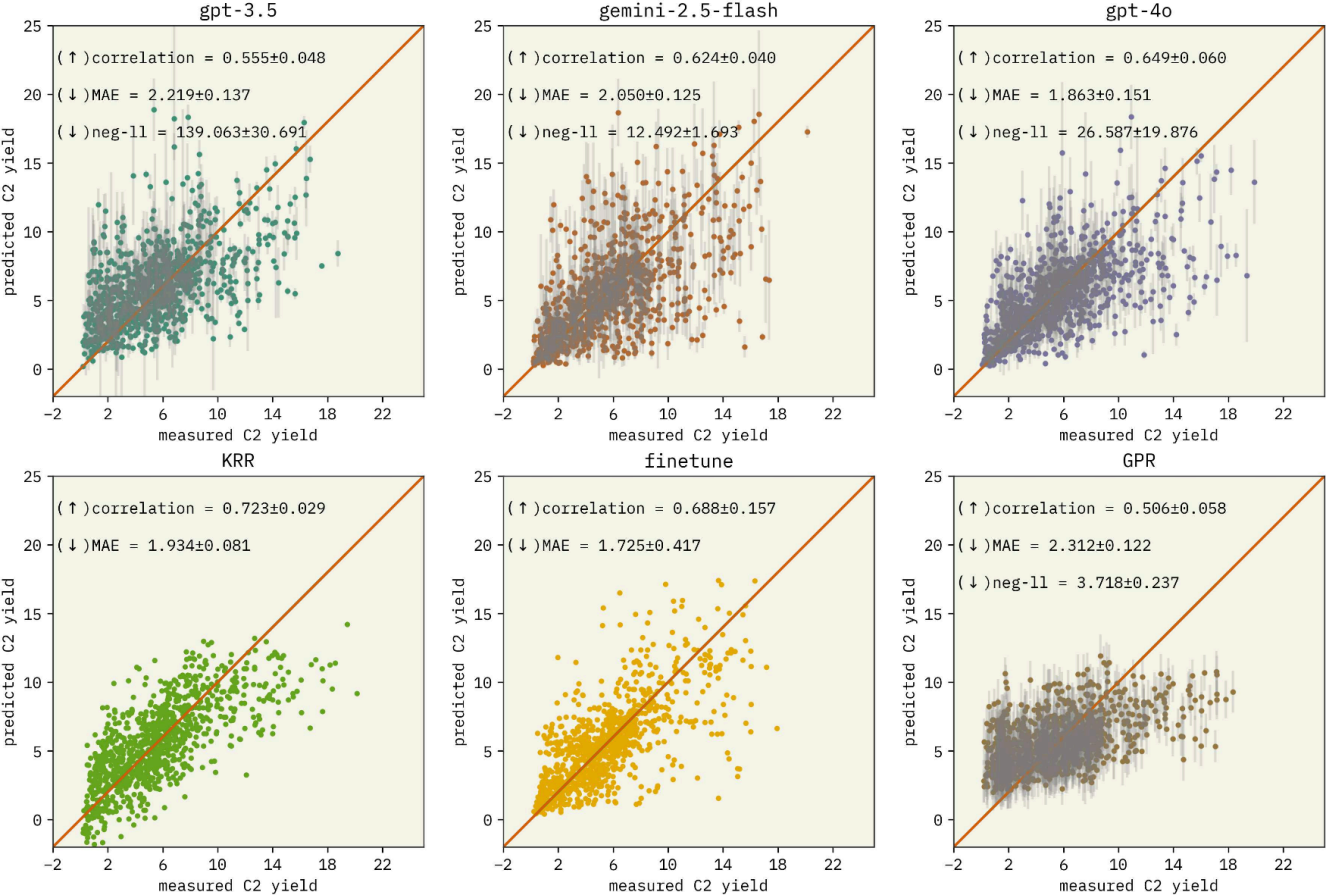
Parity plots for the regression task on the OCM data set across different models. Each model was evaluated over five independent replicates, with each plot aggregating all predicted vs true values. Reported metrics reflect the mean and standard deviation across replicates. Large language models (LLMs) exhibit comparable performance, with gpt-4o-2024–08–06 showing a slight advantage. Interestingly, kernel ridge regression (KRR) achieves the highest correlation among all models, though it was not further explored due to its lack of uncertainty estimates.

Because KRR does not produce uncertainty estimates, it is less suitable for BO, and we therefore do not explore it further. Additionally, due to the high output token cost associated with OpenAI fine-tuned models and our focus on ICL, we also do not employ the fine-tuned gpt-3.5-turbo-0125 model for the BO task.^66^

Testing on both the solubility and OCM data sets demonstrates that common machine learning performance metrics improve as the number of available few-shot examples increases (Figure 3). For example, using the OCM data set, we observe improvements with newer OpenAI models. Specifically, gpt-3.5-turbo-0125 achieves a mean absolute error (MAE) of 2.219 ± 0.137 and a correlation of 0.555 ± 0.048, whereas the newer gpt-4o-2024–08–06 attains an MAE of 1.863 ± 0.151 and a correlation of 0.649 ± 0.060 (see Table S5 for complete results). Additionally, gemini-2.5-flash performs similarly with OpenAI models in the regression task but shows better calibration, supported by the observed smaller negative log likelihood. This is an interesting characteristic for achieving accurate results with BO. With the exception of KRR, gpt-4o-2024–08–06 outperforms all other baselines in this study (see Figure 3 and Table S5). These results support our hypothesis that expanding the model’s accessible memory pool (context) thereby increases the probability of retrieving more query-relevant examples and simulates a form of continual learning. This scaling capability is particularly important for BO. Although the retrieval-augmented ICL approach does not update the models’ internal parameters over time as in traditional learning, ICL is a practical and effective strategy for adapting new data and overcoming the inherent constraint posed by the fixed context window of an LLM.

**Figure 3:**
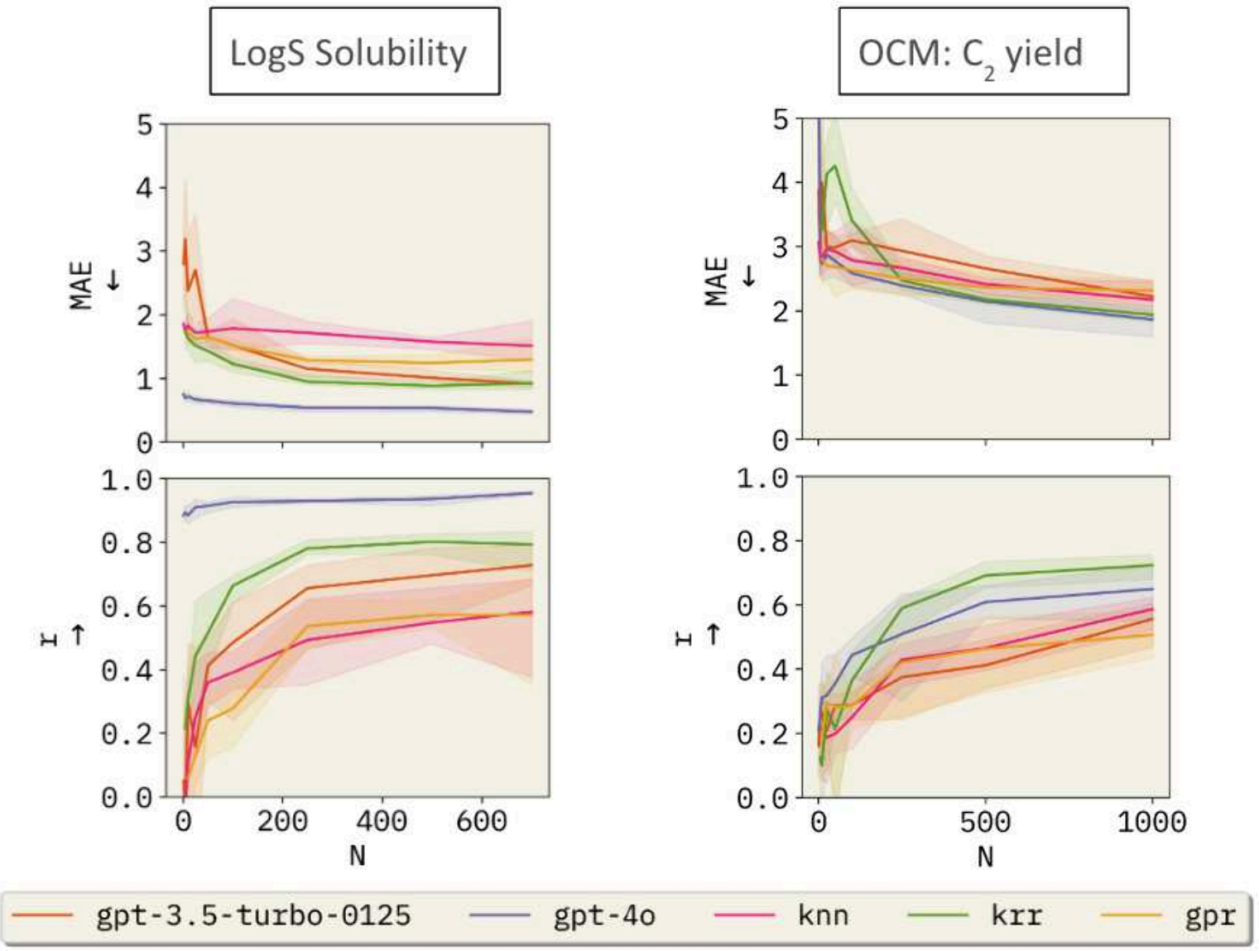
Performance comparison of baseline models versus BO-ICL based on the number of points in the model’s memory or used to train, as applicable. The top row shows the Mean Absolute Error (MAE) as a function of the number of training samples (*N*), while the bottom row shows Pearson correlation (*r*). The models compared include gpt-3.5-turbo-0125, gpt-4o-2024–08–06, Kernel Ridge Regression (KRR), *k*-Nearest Neighbors (KNN), and a Gaussian Process Regressor (GPR). The shaded areas represent the range of the predictions in each replicate, while the solid lines represent the average value.

Our regression results indicate that LLMs can predict properties and directly produce uncertainty estimates from natural-language inputs (expanded regression evaluation in Section S4). Additionally, in scenarios with abundant labeled data, ICL outperforms established methods such as Gaussian process regression (GPR) when applied to experimental procedure embeddings. Thus, we apply BO directly on language-based representations to maximize material properties within the OCM, AII, and RWGS data sets.

### 2.2 Bayesian Optimization

We first apply BO-ICL to the OCM data set, which provides an empirical, high-fidelity, and unambiguous environment for initial evaluation. In this setting, querying the black-box function *f*(*x*) corresponds to accessing the labeled data set. Details of the BO-ICL nomenclature and algorithmic implementation are provided in Section 5.

To further evaluate generality and mitigate potential data leakage, we additionally optimize procedural parameters in two distinct settings: (i) a synthetic data set representing alloy interface interactions (AII), and (ii) in-house data sets aimed at identifying synthesis and reaction conditions that maximize CO yield under RWGS reaction conditions.

Across optimization scenarios using the adopted data sets, we benchmark against Bayesian optimization with Gaussian process surrogates (BO-GP), implemented as a widely used baseline. To ensure comparability, both approaches operate on identical feature representations derived from the LLM embeddings.

We acknowledge that Gaussian process surrogates are not always naturally suited to language-derived feature spaces or to the discrete, irregular design landscapes characteristic of heterogeneous catalysis. Small changes in catalyst composition, support identity, or promoter loading can produce abrupt performance shifts that violate the smoothness assumptions implicit in many commonly used GP kernels. In practice, obtaining accurate BO-GP predictions requires multiple modeling choices and hyperparameter adjustments that are not required with BO-ICL.

We hypothesize that language-based representations in pretrained LLM embeddings provide a chemically meaningful geometry that partially regularizes the optimization landscape while allowing candidates to be specified directly as discrete, human-interpretable design descriptions without training a task-specific model.^67^ However, these embeddings are highly dimensional (1532 dimensions in this work), which can make GP training computationally expensive and numerically ill-conditioned, particularly in the small-data regime typical of catalyst screening. To enable stable BO-GP optimization, we apply dimensionality reduction (Isomap to 32 dimensions), introducing additional modeling assumptions (e.g., preservation of local neighborhood structure) and hyperparameters such as neighborhood size and target dimensionality that can influence optimization performance.^68^

BO-ICL instead uses the pretrained LLM directly as an implicit surrogate and operates on the original language representation without kernel specification, surrogate fitting, or dimensionality reduction. Candidate selection is guided through few-shot in-context examples, leveraging both the semantic structure encoded in the embedding space and the data-efficient generalization behavior of the LLM.

#### 2.2.1 Oxidative Coupling of Methane

When testing on the OCM data set, our goal in applying BO-ICL is to rediscover the optimal experimental conditions for maximizing the yield of value-added *C*_2_ products (chemical eq 2).

OCM:

2 $$2{CH}_{4}+O_{2}\to C_{2}H_{4}+2H_{2}O,\Delta H_{rxn}=-280\mathrm{kJ}/\mathrm{mol}$$

Thus, after converting the tabular Nguyen et al.^57^ data set to an unlabeled pool of possible experiments represented in natural language, we show that using an LLM as a surrogate model for BO is comparable to using GPR with identical feature vector representations. GPR is renowned as a surrogate model for BO applications and thus is a reasonable baseline for performance analysis throughout the analyses of this study.^69–71^ Results are shown in Figure 4. Details about the data set can be found in Section S1.

**Figure 4:**
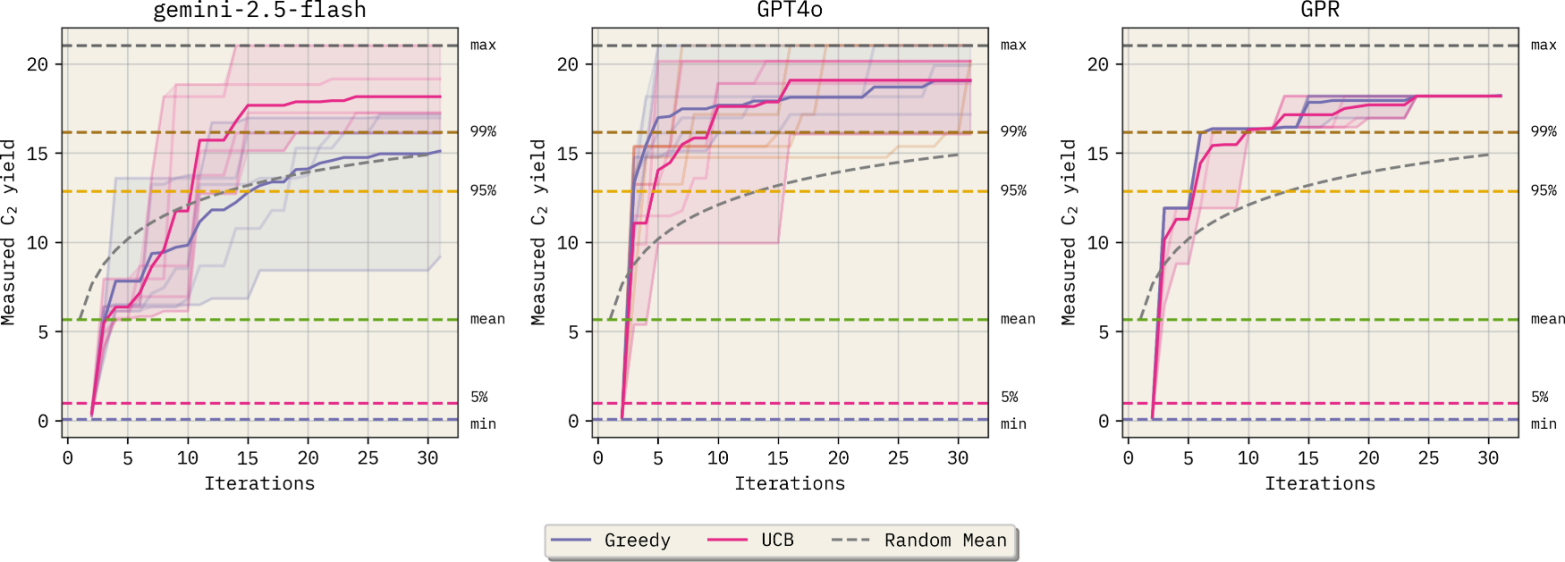
Bayesian optimization results for the OCM data set. All results use an embedded natural language representation of the sampled experimental procedures as the input feature representation. While Gemini-2.5-flash requires, on average, 12 iterations to achieve the 99^*th*^ percentile of the OCM data set distribution, both gpt-4o-2024–08–06 and GPR achieve this goal after only 10 new samples, on average. Additionally, this figure implies that GPR using LLM embeddings performs satisfactorily (for GPR specifics, see Section S3.6). The shaded areas represent the range of the predictions in each replicate, while the solid lines represent the average value.

Applying BO-ICL to the OCM data set demonstrates that gpt-4o-2024–08–06 improves convergence rates toward higher *C*_2_ yields over gemini-2.5-flash. This corroborates our findings in early regression experiments (Section 2.1). When using the upper confidence bound (UCB) acquisition function and iterating the BO loop for 30 new samples, Gemini-2.5-flash reaches the top 36^*th*^ procedure in the data set, on average, while gpt-4o-2024–08–06 achieves the top 12^*th*^. These experimental procedure rankings correspond to *C*_2_ yields of 18.16 and 19.08, respectively. It is worth noting that even though gpt-4o-2024–08–06 outperforms Gemini-2.5-flash on average, Gemini was able to find the top procedure in the data set in one of the replicates. Comparatively, GPR’s best selected point corresponds to a *C*_2_ yield of 18.19 (top 33^*rd*^). On average, both the UCB and Greedy acquisition functions (Section 5) result in the same final procedure selection with either gpt-4o-2024–08–06 or GPR surrogates. However, with gpt-4o-2024–08–06, the best possible procedure in the pool of approximately 12.8k examples is selected using the greedy acquisition function in three of the five replicates.

These results imply that optimizing experimental procedures using language-based representations is a feasible method for optimizing experimental design. It is also evident that using embedding representations for GPR is also effective for property prediction and may offer the added advantage of reproducible results. However, LLMs may still be preferable over GPR for catalytic applications due to their ability to produce comparable results without requiring kernel tuning or other complex hyperparameter optimizations associated with GPR. Thus, BO-ICL is a straightforward and ready-to-use BO strategy for property prediction in complex material spaces.

Because the OCM data set includes catalytic parameters that are well established in the literature, it is natural to ask to what extent field-specific biases might influence BO-ICL performance. In particular, prior catalysis studies on oxidative coupling of methane (OCM) often highlight *Mn*–*Na*_2_ *WO*_4_ as a top-performing catalyst, with many OCM studies published before the gpt-4o-2024–08–06 knowledge cutoff date.^57,72,73^ Notably, the BO-ICL often converges on the *Mn*–*Na*_2_ *WO*_4_/*SiO*_2_ catalyst. This raises the question of whether the apparent success of BO-ICL reflects genuine learned relationships between the natural language features and the labels, or instead arises from spurious correlations and/or data leakage.

As a control to verify that the method relies on the relationship between the natural-language features and the labels, we corrupted the OCM data set. Specifically, we sampled pseudo performance values from the same distribution as the true values and randomly assigned them to the same feature set, ensuring there is no true correlation between features and labels. As shown in Section S3, BO-ICL with Greedy and UCB converges to the analytical random-search trajectory within a similar number of iterations to those in earlier runs. This supports the important conclusion that, in the absence of a true feature–label relationship, BO-ICL is ineffective at guiding optimization within the design space.

To further address concerns that performance could be driven by pretraining-related leakage (although this is unlikely given the transformation of tabular data into natural language and the variability of reported catalytic performance across studies), we extend our workflow to the AII data set. We expect the AII data set to further reduce the risk of leakage because the objective is based on a less commonly used analytical equation for interfacial material properties and was published after relevant model training cutoff dates (Section 2.2.2).

#### 2.2.2 Estimated Alloy Interface Interaction

Using a capacitor model to describe an alloy interface, as proposed by Gerber et al.,^58^ we apply BO-ICL to relate alloy–material pairs to the maximum unidirectional charge transfer across a pool of 9k alloys. The model approximates the calculated charge-transfer labels using only Fermi levels, the transfer gap (defined as the sum of the largest van der Waals radii of the two alloys), and the alloy stoichiometric chemical formulas, each specified in natural language (see Section S1 for details).

The AII data set provides a setting designed to minimize potential data leakage, reducing the likelihood that performance gains are driven primarily by domain-specific knowledge encoded during pretraining. The original data set was published after the gpt-4o-2024–08–06 knowledge cutoff and is therefore absent from model pretraining. We additionally incorporate alloy Fermi levels from the Materials Project database, which are not explicitly reported in the original manuscript.^75^ The analytical model used to describe interfacial charge transfer also deliberately omits spin–orbit coupling effects to maintain a simplified, controlled relationship between inputs and outputs. This simplification, together with the logarithmic scaling of the charge-transfer labels, reduces the influence of highly specialized physical detail and limits opportunities for memorization-based performance. As a result, the AII data set serves as a useful test of BO-ICL under comparatively unfamiliar domain structure. The rediscovery of material pairs within the top 99*^th^* percentile of the AII data set demonstrates that BO-ICL can effectively guide materials selection under these conditions (Figure 5, left: gpt-4o-2024–08–06; center: gpt-4o-2024–08–06 + davinci-002; right: GPR baseline).

**Figure 5:**
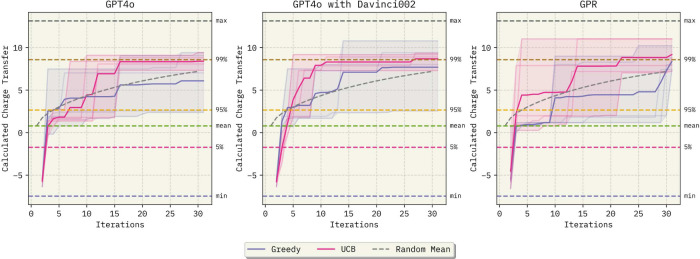
Results for the alloy interface charge-transfer data set (AII) using BO-ICL and a GPR baseline with natural-language embeddings. All three settings use the same BO loop and acquisition function; the difference is which model is used for the property-prediction/uncertainty step versus the inverse-design generation step. Left: BO-ICL using the chat model gpt-4o-2024–08–06 for both inverse design and property prediction/uncertainty estimation (workflow step *A*7). Center: mixed-model BO-ICL using gpt-4o-2024–08–06 for inverse design and the base completion model davinci-002 for property prediction and uncertainty estimation (step *A*7). Right: GPR baseline (see Section S3.6). Comparable convergence and final selected property values are observed within 30 BO iterations. The shaded areas represent the range of the predictions in each replicate, while the solid lines represent the average value.

Using the AII data set, we further examine whether different LLMs are better suited to certain inference steps within the BO-ICL workflow. For all other data sets, we use gpt-4o-2024–08–06 at every inference step (Figure 4).^76^ In contrast, for the property-value prediction and uncertainty-estimation step (workflow step *A*7), we use the davinci-002 base completion model. Empirically, davinci-002 produces better-calibrated uncertainty estimates on AII, indicating that predicted uncertainties more closely track observed prediction errors than gpt-4o-2024–08–06 (Figure 5, center). One possible explanation is that instruction-tuned models optimized with reinforcement learning from human feedback (RLHF) may trade off probabilistic calibration for human preferences, which can be disadvantageous when accurate uncertainty quantification is required.^76^

Our decision to incorporate davinci-002 comes from the observed importance of model calibration on overall performance (see Section 5.3). Using a well-calibrated off-the-shelf model for the regression step alleviates the need for posttraining calibration and reduces the number of initially labeled data points required to achieve satisfactory performance. For the inverse-design generation step (workflow step *O*1 in Section 5.2), we use gpt-4o-2024–08–06, as its RLHF training encourages an output structure that more closely aligns with the natural-language format of the experimental procedures. This alignment is particularly useful for the similarity comparison and retrieval steps in the optimization loop (workflow steps *A*2–*O*3, and Section S2). The performance differences when using a single model (gpt-4o-2024–08–06) versus a combination of a base model and a chat model (davinci-002 and gpt-4o-2024–08–06) in the workflow may further highlight the critical role of accurate uncertainty estimation when comparing upper confidence bound (UCB) trajectories (see Section 5.1 for acquisition function details)

It is important to note that observed performance on a data set like AII may relate to the use of a well-defined analytical objective function, as opposed to the other data sets relying on experimental labels, which are more susceptible to aleatoric measurement errors. Although direct comparison between the use of different data sets and models remains challenging due to replicate limitations and inherent model differences, achieving performance that outpaces random-walk baselines on complex data sets like AII is sufficient motivation to synthesize and test materials in-house, using BO-ICL to guide the experimental parameter selection for optimizing catalyst synthesis and reaction conditions (Section 2.2.3).

#### 2.2.3 In-House RWGS

To extend our workflow to scenarios where experimental outcomes are not known *a priori*, we apply BO-ICL to on-demand experiments where we synthesize and test heterogeneous multimetallic catalysts to maximize CO yield under RWGS reaction conditions (eq 3).

Reverse Water–Gas Shift:

3 $${\mathrm{CO}}_{2}+H_{2}⇌\mathrm{CO}+H_{2}O,\Delta H_{rxn}=41.2\;kJ/mol$$

Selecting an equilibrium-limited reaction imposes a thermodynamic upper bound on our objective function of the CO yield. Leveraging this bound provides a principled stopping criterion for BO-ICL when the best observed performance approaches the thermodynamic ceiling, reducing ambiguity about whether additional iterations can materially increase the observed CO yield under the same operating conditions.

Our initial RWGS design space consists of 3,720 possible catalysts, with the metals constrained to Pt-TM (Pt with one transition metal) or Pt-TM_1_-TM_2_ (Pt with two transition metals) (Table S1). Reaction conditions span 275–325 °C, with a fixed feed ratio of CO_2_:H_2_ = 1:3 at 1 atm, to selectively control carbon-based products to CH_4_ and CO for ease of evaluation across candidates (Figure S1). Figure 6 shows three closed-loop BO-ICL trajectories: a random walk (purple), BO-ICL with gpt-4 using a Greedy acquisition function (green), and BO-ICL with a chat model, gpt-4o-2024–08–06, using the Upper Confidence Bound (UCB) acquisition function (orange). The random walk represents a series of experiments that are chosen using a random number generator to provide baseline insight of the CO yield distribution within the sample space. As expected, Greedy yields near-monotonic improvement through exploitation, whereas UCB exhibits exploratory selections (e.g., iterations 4–5). Both BO-ICL trajectories identify conditions that achieve >20% CO yield within the six iterations, which is within measurement uncertainty of the calculated equilibrium CO yield for the inlet composition and reactor temperature.

**Figure 6:**
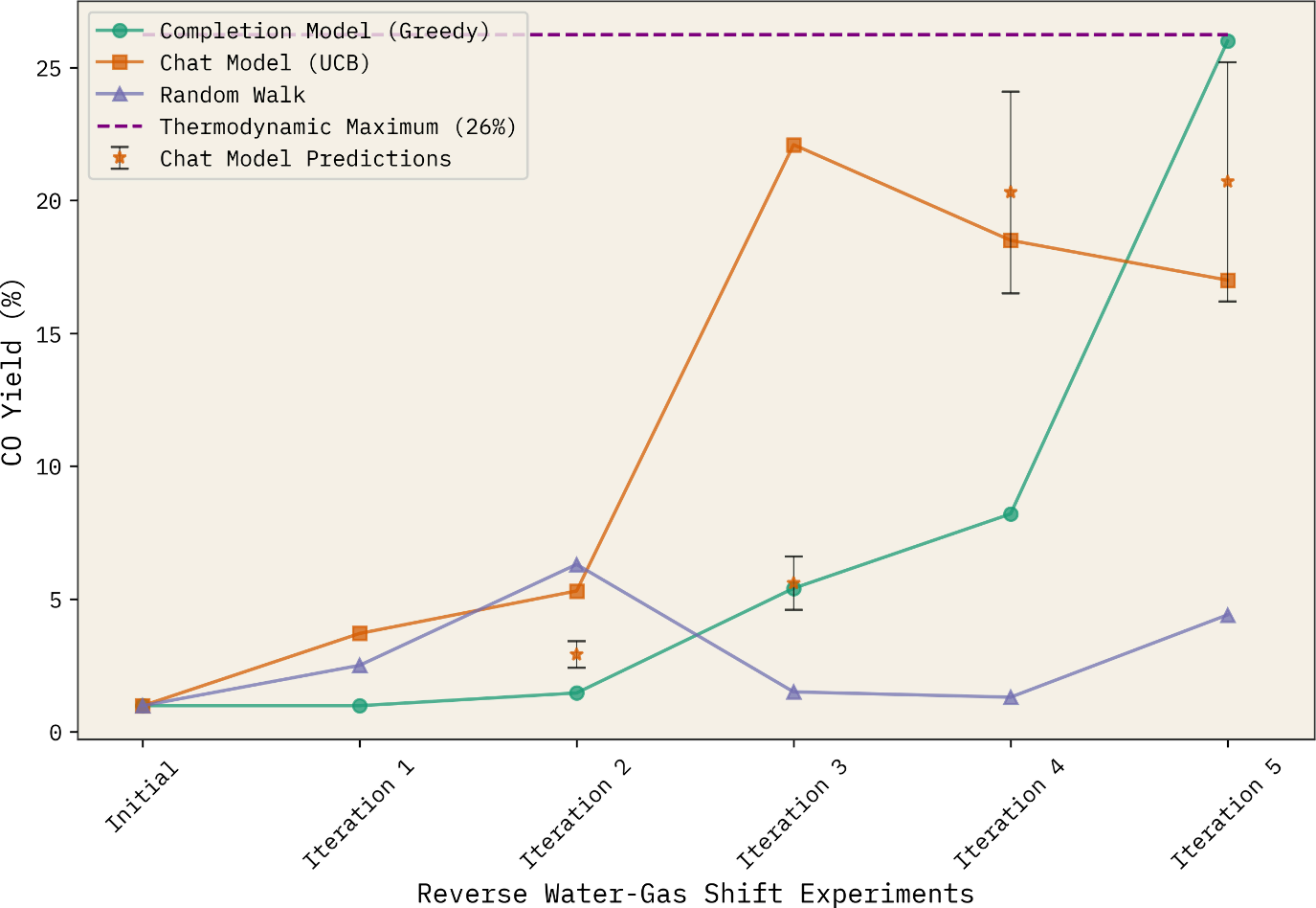
BO-ICL results on a pool of RWGS experiments. Purple: six randomly selected experiments. Green: BO-ICL with gpt-4 using a greedy acquisition function. Orange: BO-ICL with gpt-4o-2024–08–06 using UCB; stars indicate the surrogate mean prediction prior to execution, and error bars indicate model uncertainty (μ ± σ). The dashed line denotes the equilibrium CO yield computed for the inlet composition and reactor temperature (eq S5).

In the above RWGS experimental pool, we intentionally relaxed constraints such as cost to focus on evaluation of the BO-ICL method applied to an unlabeled data set. We then increased the system complexity by shifting to a new data set with less-studied, trimetallic catalysts and greatly restricted the metal combinations of abundant and safe transition metals (Section S1.4, Figure S5). Additionally, we avoid metals that are known to be highly active for CO_2_ activation (e.g., Fe, Cu, Ni, Co) (Section S1.4.2), making it more difficult for an expert in the field to intuitively select a top-performing catalyst from the pool. Our intention with this data set is to increase the size of the design space while focusing on discovery of nonobvious trimetallic catalyst formulations, whereas the first data set containing platinum focused on rediscovery of known catalysts. Under these constraints, the resulting sample space is orders of magnitude larger (360,000 possible experiments).

Given the size and complexity of this trimetallic data set, we used EI for the first 7 BO-ICL iterations (after 2 initial seed experiments) to prioritize informative sampling, and then we switched to a Greedy policy for the 10^th^ and final procedure selection, consistent with earlier OCM, ESOL, and AII BO-ICL experiments where we switched to the Greedy acquisition function for the final candidate selection. We carried out two trajectories using OpenAI models gpt-4.1–2025–04–14 and gpt-4o-2024–08–06. We capped each campaign at 10 total experiments because multiday synthesis is required to achieve reproducible results. To implicitly probe catalyst stability, reactions were run for a minimum of 12 h after pretreatment, and the CO yield was computed from averaging the performance of the final 3 h on-stream.

In expanding the BO-ICL workflow to the trimetallic data set comprising 360,000 possible experiments, we identified active catalyst compositions that are not well represented in the existing literature within just 8 iterations of the BO-ICL loop (after the 2 initial seed experiments): K@ZnZrMo(11.3:1.5:1)/CeO_2_ and K@ZnZrMo(11.3:1.5:1)/Al_2_O_3_. These catalysts were tested after CO pretreatment at 500 and 450 °C, respectively, for 2 h at 50 psi, using a GHSV of 16,000 mL gcat^–1^ h^–1^. Under the standard reaction conditions (300 °C and 1 atm), they achieved CO yields of 18.0% and 17.2%, respectively. The time-on-stream (TOS) performance of K@ZnZrMo(11.3:1.5:1)/CeO_2_ and K@ZnZrMo(11.3:1.5:1)/Al_2_O_3_ is shown in Figure 7; because an induction period was observed, an extended TOS run for K@ZnZrMo(11.3:1.5:1)/CeO_2_ is provided in Figure S6, exhibiting convergence toward the thermodynamic maximum CO yield. Notably, independent surrogate models (gpt-4o-2024-08-06 and gpt-4.1-2025-04-14) with different contexts converged on the same metal identities, ratios, and pretreatment species (Table S3).

**Figure 7:**
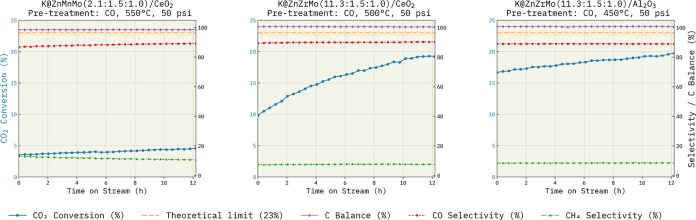
Comparative time on stream (TOS) performance of representative BO-ICL-selected trimetallic RWGS catalysts (see Table S2 for operating conditions). Left to right: K@ZnZrMo(2.1:1.5:1.0)/CeO_2_ after CO pretreatment at 550 °C; K@ZnZrMo(11.3:1.5:1.0)/CeO_2_ after CO pretreatment at 500 °C (top-performing catalyst highlighted in the main text); and K@ZnZrMo(11.3:1.5:1.0)/Al_2_O_3_ after CO pretreatment at 450 °C. Blue circles (left axis) denote CO_2_ conversion, while red squares and green triangles (right axis) denote CO and CH_4_ selectivity, respectively; purple diamonds indicate carbon balance. The dashed orange line marks the RWGS-only equilibrium CO_2_ conversion (23%). The best-performing K@ZnZrMo/CeO_2_ catalyst exhibits an induction period, with CO_2_ conversion increasing monotonically to ∼20% while maintaining high CO selectivity and >95% carbon balance over the 12 h TOS.

Although alkali promotion (K), reducible oxides (e.g., CeO_2_, ZrO_2_), and transition metal carbides are often associated with CO_2_ activation, these factors alone do not explain the catalyst performance, as closely related compositions such as K@ZnZrMo(2.1:1.5:1.0)/CeO_2_, compared in Figure 7, are significantly less active.^53^ The selection of Mo may appear intuitive, given prior reports of bulk Mo_2_C activity under RWGS conditions; however, under the selected pretreatment protocols, formation of a fully carbidic bulk Mo_2_C phase is not necessarily expected. Prior carburization studies commonly report oxycarbide-rich materials at 500–600 °C and complete carburization at higher temperatures (e.g., 700–800 °C), depending on the exact protocol and carbon chemical potential.^77^ If carbides form under these pretreatement conditions, these yet-to-be characterized trimetallic interactions may be responsible for accelerating the rate of carburization.

## 3 Limitations and Practical Considerations

Across our tests of BO-ICL, we observed several limitations that stem from both standard Bayesian optimization (BO) considerations and the nondeterministic behavior of LLM-based in-context learning (ICL) when used as an updating surrogate.

### 3.1 Initialization and Exploration Coverage

A practical requirement of BO is that the initial evaluation set provides adequate coverage of the design space (i.e., diversity and performance), because early samples strongly influence the surrogate posterior and therefore the acquisition-driven exploration–exploitation trade-off.^78,79^ Similarly, in early BO-ICL experiments, we found that a low diversity in the initial prompts can induce procedural and local bias; in these cases, simply increasing nominal exploration parameters (e.g., the UCB scaling factor λ; Section 5) is not always sufficient to recover broad exploration.

To reduce this failure mode, we seeded BO-ICL with diverse initial experiments. After selecting an initial reference point, we compute cosine similarity between its embedding and all remaining candidates and select the most dissimilar point (a farthest-point heuristic) to construct a maximally diverse initial context. In our experiments, we use *k* = 2, but this approach generalizes to larger *k* by iteratively selecting the candidate with the largest minimum distance to the running labeled set. When larger labeled data sets are available, practitioners should explicitly balance allocating labeled data to seed a diverse BO-ICL context set versus reserving labeled data for supervised fine-tuning or calibration, since both choices can materially affect exploration behavior and sample efficiency. While this initialization strategy improves coverage in practice, it does not guarantee global exploration, particularly in very large or strongly multimodal design spaces.

### 3.2 Uncertainty Calibration and Transferability

Uncertainty calibration is an additional limitation. For global optimization, BO relies on reasonably accurate uncertainty estimates to guide exploration.^28,34^ However, modern LLMs—particularly those aligned by RLHF—can exhibit miscalibrated confidence, complicating uncertainty estimation without substantial validation data^76,80^ (Section 5.3). This requirement partially conflicts with BO’s advantage of optimizing objectives with minimal data. In this work, we mitigate calibration challenges by (i) using base models that empirically yield more stable uncertainty behavior in our setting and (ii) leveraging transfer when a calibration mapping can be learned from a related data set. For example, as described in Section 5, we derive an uncertainty scaling factor using a validation subset from the OCM data set (via Uncertainty Toolbox) and find that this scaling improves BO-ICL behavior beyond the OCM (e.g., AII, ESOL, and RWGS). While calibration is often treated as domain- and data-set-specific, these results suggest that cross-domain calibration transfer can sometimes be a practical compromise when only limited validation data are available.

We emphasize that the cross-task transferability of a fixed uncertainty scaling factor should not be assumed *a priori*. In our experiments, a value tuned on OCM (e.g., a multiplicative factor of 5) improved acquisition behavior on other tasks, but this should be interpreted as an empirical observation rather than a guaranteed property of BO-ICL. A plausible explanation is that the scaling primarily compensates for systematic miscalibration in the surrogate’s uncertainty magnitude (i.e., an overall amplitude mismatch) rather than encoding task-specific structure. In small-data ICL settings, uncertainty estimates can be systematically mis-scaled due to limited context, heteroscedastic noise, and model-form mismatch; a single multiplicative factor can therefore act as a global calibration “temperature” on the uncertainty term. This is consistent with standard BO practice, where UCB-style acquisition functions include an exploration coefficient (e.g., β) that rescales uncertainty to set the exploration–exploitation trade-off. Accordingly, we treat the scaling factor as a tunable hyperparameter and recommend reselecting it when transferring to substantially different tasks or noise regimes.

### 3.3 Hallucinations and Inverse-Design Constraints

Hallucinations are particularly salient during the inverse-design step of BO-ICL (steps A1–O1 in Section 5.2), where the model may propose infeasible or irrelevant procedures, reducing search efficiency during subpool candidate population (steps O1–O2). A practical hedge is to constrain inverse-design outputs to predefined design parameters (e.g., via a custom system message) so generated candidates remain within the application’s admissible design space (Section S5). This constraint is especially important when BO-ICL uses retrieval or retrieval-augmented generation (RAG)-style components (steps A4–A8 in Figure 8). Support for the importance of procedure structure and format is presented in Section S7 (Figures S17, S18), where we evaluate the relative impact of linguistic form versus chemical knowledge by analyzing variations in similarity scores when a reference procedure is compared with relevant counterfactuals, which may substantially influence subpool composition.

**Figure 8:**
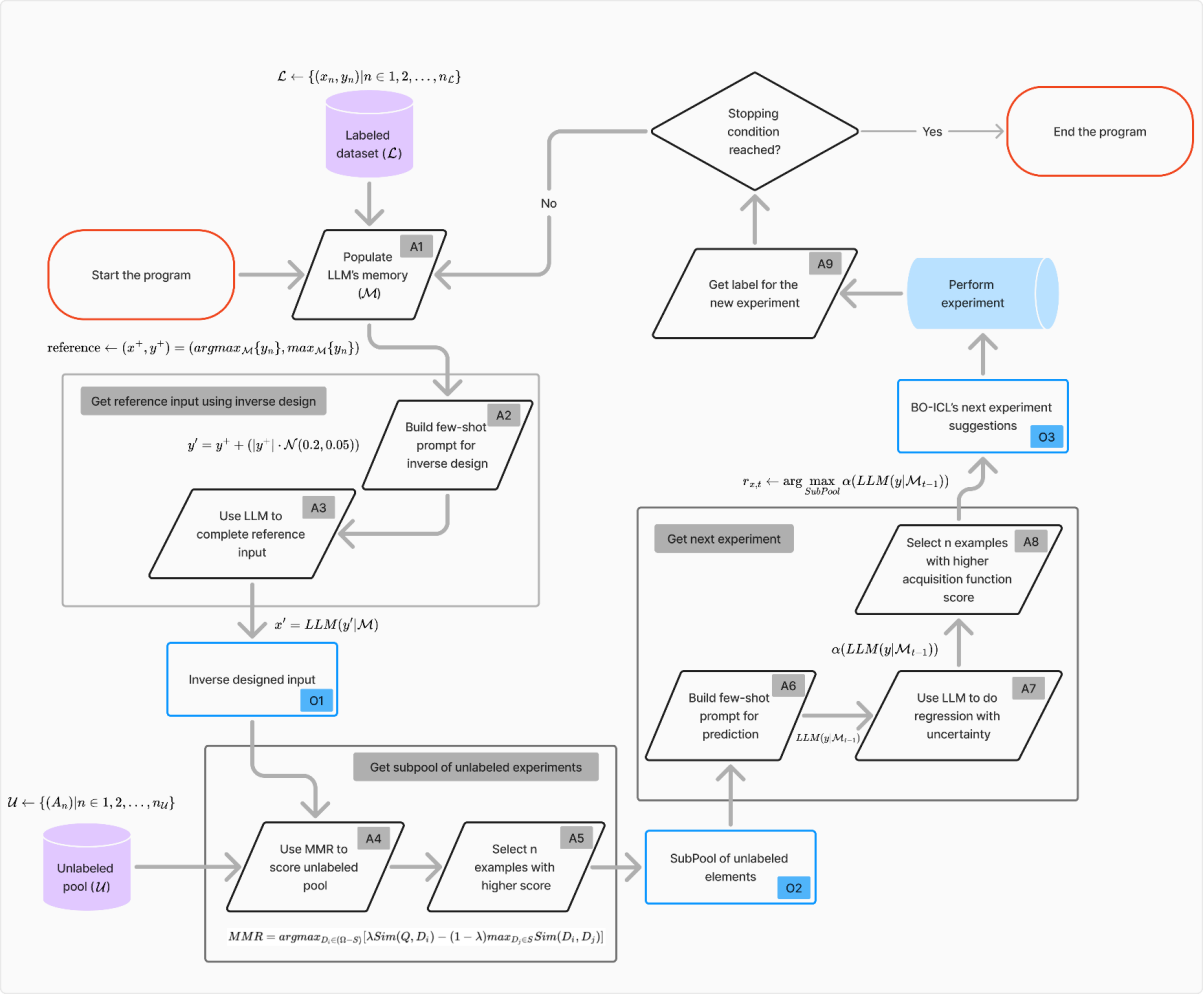
Flowchart diagram of the information flow in BO-ICL. Angled black rectangles represent actions; blue rectangles highlight key objects used in the workflow. Some actions with a common goal are grouped together within a gray box, with a label describing its goal. Actions are identified using the *An* indexer, and objects use the *On* syntax. *n* is an index without further meaning. The same pipeline is shown in Section S2 using pseudocode.

### 3.4 Subpool construction and candidate-space coverage

A related limitation arises from subpool construction. In the default BO-ICL implementation, we use a single inverse-design call followed by maximal marginal relevance (MMR) to populate a subpool and reduce token cost and inference latency. While efficient, this single-round inference-sampling (IS) step can, in principle, uncover the global design space and exclude candidates with high acquisition values that lie outside the retrieved region, since BO is effectively performed over a filtered subset rather than the full pool. We adopt a single IS round to limit hyperparameter proliferation and improve reproducibility, as multiple inverse-design/MMR passes introduce additional user-defined settings (e.g., number of IS rounds and intermediate pool sizes) and increase the LLM query cost. Empirically, we observe stable performance across data sets with a single IS round, suggesting this added complexity is unnecessary for the problem classes studied here. However, for very large, highly heterogeneous, or strongly multimodal design spaces, increasing the number of IS rounds and/or enlarging the candidate set prior to MMR may improve coverage and mitigate selection bias; thus, the number of IS iterations can be treated as a tunable parameter that trades off cost against design-space coverage.

### 3.5 Stochasticity, Subsampling, and Evaluation Variance

Evaluation is further complicated by the stochasticity of the LLM generation. Fixing hyperparameters (e.g., low temperature and a consistent sampling strategy such as top-*k*) can reduce, but not eliminate, run-to-run variation, particularly for closed-source models. Deterministic surrogates such as Gaussian processes do not share this drawback. Accordingly, we estimate average performance by running five replicates per acquisition configuration and using nonparametric comparisons, while noting that small-*n* uncertainty remains. Notably, stochasticity can also act as an implicit source of exploration and occasionally aid novelty.

Subsampling is used across data sets to reduce API latency and inference cost, but it introduces sampling variance and can obscure the true behavior of the optimization policy. When the candidate pool is small enough that cost and latency are not limiting, practitioners should avoid subsampling and instead evaluate the full candidate set directly, since exhaustive scoring eliminates sampling variance and provides the most faithful assessment of BO performance. We did not include a full global predictive-search baseline in this study because our focus is the regime where BO is typically most useful—large candidate spaces where exhaustive evaluation is impractical—and because full-space LLM querying can be expensive and energy-intensive at scale. More generally, the appropriate choice depends on candidate-space size and resource constraints: full evaluation is preferable when feasible, while subsampling is a practical compromise when throughput, monetary cost, or environmental considerations dominate.

### 3.6 Retrieval Scaling and Context-Budget Effects

As the BO loop progresses, the labeled set *L* (and memory *M*) grows, so a fixed retrieval count *k* is not guaranteed to remain optimal. We nevertheless keep *k* fixed as a pragmatic design choice for three reasons. First, our sensitivity analysis (Figure 9) shows that varying *k* in the tested regimes yields no consistent improvement, suggesting a trade-off between adding helpful demonstrations and diluting relevance under a finite context budget. Second, keeping *k* modest is consistent with prior findings in in-context learning that gains can saturate or degrade as demonstrations increase.^65^ Third, while *k* is fixed, retrieval is dynamic: at each iteration the *k* demonstrations are reselected from the current *M* based on relevance, so the method benefits from improved memory quality primarily through better example selection rather than more examples. We view adaptive *k* schedules (e.g., increasing *k* with |*M*| or conditioning on retrieval confidence/diversity) as a natural extension, but we do not study them here because long-context behavior varies across models and inference stacks.

**Figure 9:**
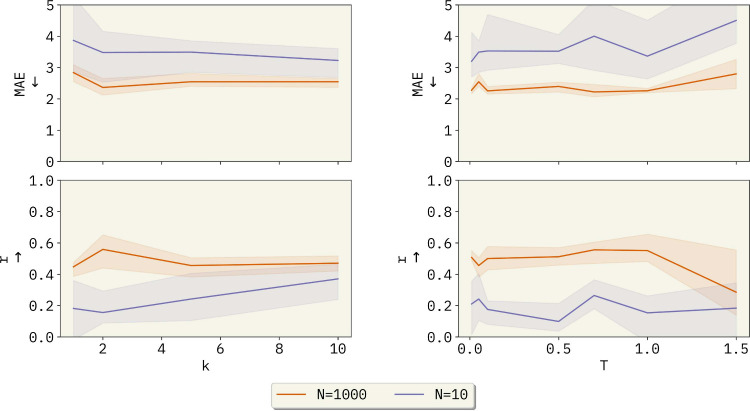
Analysis for hyperparameter selection. Varying *k* (the number of context examples per prompt) and the temperature (*T*) for gpt-3.5-turbo-0125 controls the spread of the output distribution over the model’s vocabulary and tunes the degree of randomness.

### 3.7 Pretraining Bias, Novelty, and RWGS-Specific Experimental Constraints

A major strength of using general-purpose models as surrogates is their broad base knowledge acquired through large-scale pretraining; however, this same prior knowledge can be a liability. While transfer learning can accelerate BO-ICL convergence, it can also introduce domain-specific biases because pretrained models may preferentially suggest literature-familiar designs, constraining novelty in the inverse-design step. Our working hypothesis is that exploratory acquisition functions (e.g., UCB) can partially counteract this tendency by repeatedly rewarding high-uncertainty candidates. Prior work also suggests that when prompts are less aligned with the pretraining distribution, performance relies more heavily on the provided in-context examples than on broad priors, increasing the influence of curated context.^64,81^ A complementary strategy to promote novelty is to carefully construct the candidate pool to include less-studied combinations, encouraging evaluation outside common design landscapes.

We probed this limitation by making the final RWGS campaign intentionally more challenging and expanding the catalyst design space to 360,000 trimetallic compositions. This constraint steers the search toward less-explored catalysts, reducing reliance on default literature-familiar suggestions and increasing the extent to which performance reflects the BO-ICL procedure itself (Section S1.4). In this in-house RWGS data set, fixed time-on-stream (TOS) evaluation reveals an additional practical limitation: several highly ranked catalysts exhibit induction behavior and may not reach steady state within the 12 h window. As a result, the performance computed from a fixed window can under- or overestimate steady-state performance, complicating comparisons across catalysts. Future campaigns could mitigate this issue by extending the evaluation for finalists, adopting adaptive stopping criteria, or explicitly modeling induction dynamics.

### 3.8 Safety and Deployment Considerations

More broadly, the BO-ICL performance and safety depend critically on how the design space is constructed and constrained. Poorly informed candidate specifications or operating conditions can waste resources and, in the worst case, introduce hazards (e.g., insufficient heat removal or thermal runaway risk in exothermic regimes). BO-ICL deployments should therefore be grounded in standard experimental safeguards, including operability constraints, compatibility checks, and expert review, when expanding into unfamiliar chemistries or operating regimes before finalizing and testing the candidate set.

## 4 Conclusions

This work introduces BO-ICL, a framework that integrates Bayesian Optimization (BO) with In-Context Learning (ICL) via large language models (LLMs) to optimize experimental conditions directly from natural language representations. We demonstrate the effectiveness of BO-ICL across four data sets: solubility (ESOL), oxidative coupling of methane (OCM), alloy interface interaction (AII), and reverse water–gas shift (RWGS). On the OCM data set, BO-ICL reaches the 99th percentile of candidate procedures using only ten additional samples, matching the performance of Gaussian Process Regression (GPR) with natural language embeddings. Moreover, the BO-ICL successfully guides real-world RWGS catalyst experiments, achieving CO yields near the thermodynamic limit. More broadly, the RWGS case study highlights the workflow enabled by BO-ICL, which is rapid convergence on novel and active catalysts, reducing time and experimental resources spent on suboptimal formulations.

Our results highlight that LLMs are practical surrogates for BO by leveraging their scalability through example-based reasoning. Unlike traditional approaches, BO-ICL operates without feature engineering, architectural tuning, or retraining, making it a zero-shot, task-agnostic solution for design optimization in catalysis and materials science. BO-ICL is a reliable and accessible framework for accelerating experimental design, using natural language as a universal chemical representation, enabling optimization with minimal computational resources, thereby eliminating the need for task-specific fine-tuning or feature selection (soft cost analysis in Section S6). The framework is available open-source at https://github.com/ur-whitelab/BO-ICL.

## 5 Methods

### 5.1 Bayesian Optimization

BO is a sequential, gradient-free strategy for optimizing an expensive to evaluate black-box function *f*(*x*).^28^ BO is particularly useful in settings where direct evaluation of the objective function is costly, such as catalysis-focused wet-lab research. BO aims to solve the optimization problem

4 $$\arg\underset{x\in \Omega }{\max}f\left(x\right)$$

where Ω is typically a hyper-rectangle domain that limits the set of possible experiments (eq 4). We call Ω the sample space.

In order to run BO, a surrogate model S(x) is used to approximate the expensive-to-evaluate black-box function *f*(*x*). Surrogate models are often probabilistic, offering query predictions along with corresponding uncertainty estimations at inference. GP models are commonly used as surrogates.

Initially, the prior S(x) is trained using all already available data D . Then the posterior probability distribution can be computed as S(x|D) . On each iteration, the probabilistic model is used to compute a set of posterior probability distributions and an acquisition function α­(*x*) is used to rank and select the next sample to evaluate. Most acquisition functions use the prediction mean (μ­(*x*)) and uncertainty (σ­(*x*)) to balance the trade-off between exploring uncertain regions of the input space and regions where the surrogate model predicts high values for *f*(*x*).

In this work, we focus on three acquisition functions: The Upper Confidence Bound (UCB), which balances exploration and exploitation by incorporating both the mean and uncertainty: α_UCB_(*x*) = μ­(*x*) + *λσ*(*x*), where λ is a tunable parameter that controls the exploration-exploitation trade-off. Another acquired function considered was the greedy acquisition function. This function always selects the point with the highest predicted mean from the surrogate model, favoring exploitation. The greedy acquisition function can be expressed as α_greedy_ = μ­(*x*). Lastly, we employed random sampling as a baseline. The random sampling selects the next point to evaluate using a random number generator to define an index to select from the sample space Ω. In this case, the next experiment is selected as *x*_next_ ∼ Uniform­(Ω).

In the sequence, the black-box function *f*(*x*) is evaluated to obtain the label for the selected point, which is then added to the training data set D for the next iteration of the BO policy.

The BO algorithm proceeds iteratively as follows:
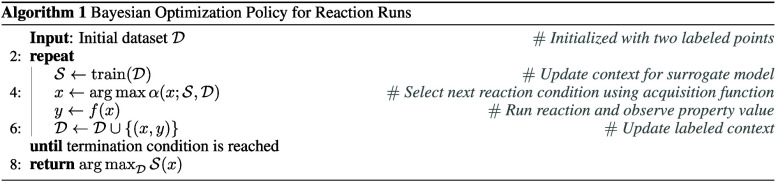


### 5.2 BO-ICL Workflow

BO-ICL leverages LLMs as surrogate models for the BO of select parameters. We use ICL to dynamically update the posterior inference using labeled examples. To ensure scalability with new data, we implement a long-term memory of labeled samples, allowing the use of a relevant context for prompt construction. By dynamically generating prompts, we show that model performance can improve even beyond its context window (i.e., the maximum amount of input data the model can process at once) as new data is acquired (Section 2.1).

We use cosine similarity with the query of focus as the reference to downsample the labeled pool for prompt generations. Thus, for each query, often an unlabeled experimental procedure, we identify the most relevant examples and prefix them for the ICL at inference time. This prompt generation process uses LangChain^82^ and the available FAISS library,^83^ along with Ada-002 embeddings.^84^

The queries follow a general prompt structure for LLM input: {prefix}­{few-shot template}­{suffix}. The {prefix} provides instructions and constraints for the task, including the expected response format, to minimize hallucinations: a procedural output outside of the design search space. This step, often implemented as a system_message, is especially important for guiding the chat model behavior. Including the task description in the system_message significantly improves the performance, as shown in Section S5.

The {few-shot template} formats the context by concatenating *k* examples using the following structure: “Given {representation}. What is {property_name}? {completion}”. Figure 1 illustrates how the prompt is constructed by selecting *k* = 1 examples as the context. In all BO-ICL experiments presented, we use *k* = 5; if available context count is less than *k*, all available context is included in the prompt. Finally, {suffix} contains the primary query of interest for which the LLM should provide a completion.

For the regression steps with uncertainty, we use token probabilities, following an approach similar to the action selection process described in Ahn et al.^85^ To estimate model uncertainty, we marginalize the logarithmic probabilities of the completion tokens to derive a discrete probability distribution after *n* iterations (eq 5). This distribution can then be leveraged for weighted uncertainty approximations, which are directly applied within the acquisition functions for BO^28^

5 $$\sigma =\sqrt{\frac{\overset{N}{\underset{i=1}{\sum }}w_{i}{\left(x_{i}-{\mathrm{x̅}}^{*}\right)}^{2}}{\frac{\left(N-1\right)}{N}\overset{N}{\underset{i=1}{\sum }}w_{i}}}$$

where *N* is the total number of observations, *x*_*i*_ means the value of the *i* ^*th*^ observation. We represent the weighted mean of the observation as ${\mathrm{x̅}}^{*}$ , calculated as ${\mathrm{x̅}}^{*}=\frac{\overset{N}{\underset{i=1}{\sum }}w_{i}x_{i}}{\overset{N}{\underset{i=1}{\sum }}w_{i}}$ . Finally, *w*_*i*_ is the weight assigned to the *i* ^*th*^ observation, reflecting its relative importance or observation probability.

Finally, these methods are combined into a BO loop to optimize experimental parameters. This is advantageous because the BO approach requires no traditional training and has minimal compute requirements for inference. A flowchart illustrating the implementation of BO-ICL is provided in Figure 8, and a pseudocode implementation is available in Algorithm S1.

BO-ICL starts by using an optional labeled data set L to populate the LLM long-term memory M (step *A*1 in Figure 8). If L is not available, the LLM initiates the optimization without prior knowledge of the space of possible experiments. Typically, the surrogate model is used to evaluate the entire space of possible examples U . However, due to the computational cost of using LLMs and the latency associated with API calls, we adopt an embedding-similarity retrieval approach to subsample U for the regression step (steps *A*5–*A*7).

We create a subpool by using MMR, with an inverse-designed completion serving as the reference embedding for retrieval.^86,87^ MMR aims to reduce redundancy in the sampled set while ensuring that the selected points remain relevant to the query. We use cosine similarity to compare the Ada embedding representations. MMR is computed as shown in eq 6, and a pseudocode implementation is provided in Algorithm S2. Results exhibiting exclusive reliance on the inverse design procedures support the importance of this step in the BO-ICL algorithm in Section S3.3.

6 $$MMR=\underset{d_{i}\in \Omega \backslash S}{argmax}\left[\lambda Sim\left(d_{i},q\right)-\left(1-\lambda \right)\;\underset{d_{j}\in S}{\max}Sim\left(d_{i},d_{j}\right)\right]$$

To obtain this reference procedure, we first search M for examples with labels similar to the current best label *y* ^+^ (step *A*2). These examples are used as context to query a new procedure *x*′ corresponding to a slightly higher predicted label *y*′, defined as

7 $$y^{\prime }=y^{+}+\left(\left|y^{+}\right|\cdot N\left(0.2,0.05\right)\right),,x^{\prime }=LLM\left(y^{\prime }|M\right)$$

Here, *x*′ is the inverse-designed input (object *O*1), representing a hypothesized experiment with a label greater than *y* ^+^ (eq 7). We then use *x*′ as a reference to retrieve *n* similar experiments from U using MMR (steps *A*4 and *A*5). These *n* experiments form the subpool (object *O*2), which is passed to the regression step (step *A*7) to select the next experiment (object *O*3). As with the inverse design step, we construct a dynamic prompt context for each experiment *x* in the subpool by searching M for the most similar examples (step *A*6), using cosine similarity. The LLM is then used to predict a label *y* for each *x* in the subpool (step *A*7), and these predictions are scored using an acquisition function α. The top *n* candidates, based on α, are selected (step *A*8).

Next, we obtained the ground-truth labels for the selected experiments (step *A*9). For the ESOL, OCM, and AII data sets (see Section S1), the label is directly queried from the available data sets. In the case of the in-house RWGS unlabeled data sets, the experiments proposed by BO-ICL are physically run and analyzed to determine the corresponding labels (step *A*9). The optimization loop continues until a specified stopping criterion is met (e.g., when the sample selected maps to the thermodynamic maximum performance). Until that point, newly labeled experiments are added to M , and the loop proceeds. Upon reaching the stopping condition, the experiment with the highest observed label *y* ^+^ is retrieved from M .

### 5.3 Hyperparameter Tuning

Our algorithm requires defining key hyperparameters, including the number of few-shot examples (*k*) used as context and the temperature (*T*), which controls sampling for the LLM’s output. To investigate the effects of these hyperparameters, we conducted a systematic study by varying both *k* and *T* using gpt-3.5-turbo-0125, given its reduced cost.

For the systematic study, we first fixed *T* = 0.05 and *N* = 1000 for the OCM data set, or *N* = 700 for ESOL. The orange curves in Figure 9 show that our system is weakly influenced as a function of *k*. Results for both *k* = 5 and *k* = 10 lie around a mean absolute error (MAE) of ∼2.5 and a correlation of ∼0.5 . These two results are not statistically different with a *p*-value of 0.985 (Table S4).

This result is somewhat counterintuitive. To further investigate why the number of examples in context does not affect the model, we performed the same analysis but added only ten random examples to the LLM’s memory. Figure 9 (blue curve) shows a small MAE decrease from 3.490 ± 0.380 to 3.224 ± 0.361, while the correlation increased from 0.241 ± 0.114 to 0.370 ± 0.073, likely highlighting the importance of context in the low-data regime. These results corroborate with literature in observation of diminishing returns from extended context lengths.^88^

These results, along with the relationships shown in Section S4 (Figures S12–S16), may indicate varying degrees of bias influenced by the model’s pretraining familiarity with different data sets. For example, the solubility data set, where correlation values for gpt-4o-2024–08–06 reach 0.9 (Section S4.1) with minimal available examples, suggests a higher level of familiarity compared to OCM (where *r* ≈ 0.6). This aligns with the expectation that models rely more on prior knowledge in familiar settings but depend more heavily on in-context data in less familiar test spaces.^81^

Similarly, we fixed *k* = 5 to run the systematic study for *T*. The *T*-test studies (Section S4) show that differences in results for experiments with *T* within the range 0.1 to 1.0 are not statistically significant. However, we observed a considerable decrease in performance for *T* > 1.0 (Figure 9), caused by increased hallucination in the LLM outputs. The temperature variation effects are also related to the degree of model calibration.

We acknowledge that some of the models explored in this study were trained using reinforcement learning from human feedback (RLHF), which can lead to less calibrated probability estimates during inference.^76,89^ Instruction tuning with RLHF may introduce biases in a model’s output probability distribution due to subjective human annotations, potentially resulting in poor confidence estimates.^76^ Given that BO policies rely on accurate likelihood representations, we first sought to quantify the calibration of relevant models using uncertainty estimations extracted as mentioned in Section 5.2.

To assess the level of miscalibration between the predictive methods for uncertainty extraction, we utilized the ‘Uncertainty Toolbox’ (UCT)^90,91^ package. UCT provides tools to calculate calibration metrics such as the calibration error and prediction interval coverage probability. Validation samples were grouped based on their model prediction uncertainties to form confidence intervals for binning inferred values. The model’s prediction accuracy was then evaluated for samples that fall within each confidence interval to analyze how well the predicted intervals align with observed outcomes. The relationship between the predicted and observed proportions was used to plot the calibration curve and compute the miscalibration area (MA), which quantifies the deviation from the ideal, monotonic calibration curve.

The MA can then guide the optimization of an uncertainty scaling factor expected to enhance calibration. Figure 10 illustrates calibration differences with and without applying this scaling factor, using 1000 points from the OCM data set for evaluation, along with a comparison of the uncertainties using the two aforementioned extraction methods. A validation set (25 samples) used from the OCM data set was used to optimize this scaling factor; beyond the use of 25 points, exhibited nominal variation in the MA of *gpt-3.5-turbo-0125*. Interestingly, applying this calibration factor during testing of BO-ICL across different data sets consistently displayed performance improvements (see the SI). This observation is notable, as calibration is often considered a subjective process, with parameter effectiveness typically varying between tasks and data sets. The ability to calibrate models effectively using a small number of samples from a single data set, may further indicate the transfer learning potential of these SOTA LLMs.

**Figure 10:**
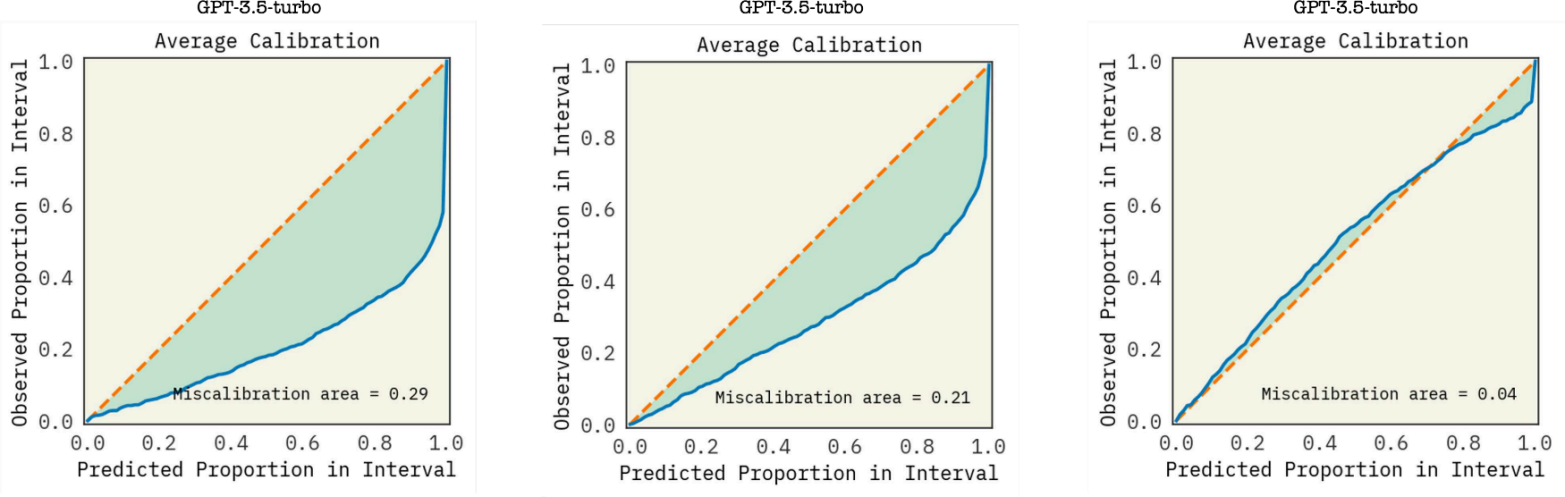
Comparison of calibration estimates for gpt-3.5-turbo-0125 on 1000 OCM data points, using five repeated predictions per prompt. We evaluate calibration using three approaches: (left) conditional probabilities to compute a weighted standard deviation as a per-prediction confidence score; (center) the standard deviation across repeated predictions as a consistency-based uncertainty estimate; and (right) a fitted scaling factor applied to quantify calibration error. Notably, increasing the calibration set beyond 25 samples (e.g., to 100, 250, or 1000) did not yield a measurable improvement in calibration.

As supported in the literature, using simple consistency arguably offers a greater degree of calibrated uncertainties over a model’s inferred distribution *p*(*y*_*i*_|θ, *x*_*i*_) following preference or instruction tuning (Figure 10).^92,93^ Based on this analysis, we defined the hyperparameters as *k* = 5, *T* = 0.7, and a calibration factor of 5. These values were used for all BO experiments presented in the main paper.

## Supplementary Material





## Notes

Data sets (solubility; oxidative coupling of methane; alloy interface; in-house RWGS); BO-ICL algorithms; baselines (analytical random; OCM with no true correlation; *k*-nearest neighbor; kernel ridge regression; Gaussian process); additional results (solubility; regression – OCM); prompts and system messages; cost analysis; effect of variation in language expression (PDF)
Transparent Peer Review report available (PDF)
