# Supplementary material for: Bayesian Optimization of Catalysis with In-Context Learning
Source: ACS Cent Sci. 2026 Apr 14;12(5):599–615. doi: 10.1021/acscentsci.5c02418 (PMC13220214; doi:10.1021/acscentsci.5c02418)
Supplement: Supplementary file 1 [file oc5c02418_si_001.pdf]

# Supporting Information for:
# Bayesian Optimization of Catalysis with In-Context Learning


**Mayk Caldas Ramos**[*,2]
mcaldas@edisonscientific.com

**Shane S. Michtavy**[*,1]
smichtav@che.rochester.edu

**Marc D. Porosoff**[†,1]
marc.porosoff@rochester.edu

**Andrew D. White**[†,1,2]
andrew@edisonscientific.com

[1] Department of Chemical and Sustainability Engineering, University of Rochester, Rochester, NY 14627, USA
[2] Edison Scientific Inc., San Francisco, CA 94107, USA

[*] These authors contributed equally to this work.
[†] Corresponding authors


## Contents of Supporting Information


**1.** Datasets . . . S2

**1.1.** Oxidative Coupling of Methane . . . S2
**1.2.** Solubility . . . S3
**1.3.** Alloy Interface . . . S3
**1.4.** In-house RWGS . . . S4

**2.** BO-ICL Algorithms . . . S10

**3.** Baselines . . . S13

**3.1.** Analytical Random . . . S13
**3.2.** BO dependence control experiment using OCM dataset . . . S15
**3.3.** OCM with No True Correlation . . . S14
**3.4.** k-Nearest Neighbor . . . S16
**3.5.** Kernel Ridge Regression . . . S16
**3.6.** Gaussian Process . . . S16

**4.** Additional Results . . . S18

**4.1.** Solubility . . . S20
**4.2.** Regression – OCM . . . S21

**5.** Prompts and System Messages . . . S24

**6.** Cost Analysis . . . S27

**7.** Effect of variation in language expression . . . S27

# S1 Datasets

To validate this workflow, we focus on application areas of global significance, such as catalytic material design for green house gas (GHG) upcycling. Accelerating material discovery in this area can reduce reliance on crude oil used for high-demand chemical precursors by using relevant waste C1 species like $CO_2$, to close the emission loop and life cycle[1]. Catalytic materials can play a major role in promoting each step in such a circular carbon economy by helping to offset related cost. These materials allow one to selectively exploiting the hysteresis gap in relevant thermodynamically controlled chemical processes. For example, inorganic catalytic materials are well studied to selectively reduce $CO_2$ (C=O bond separation energy of 432 kJ/mol) for rapid conversion of CO to valuable $C_2$ to $C_8$ products ($CO_2$ Fischer-Tropsch synthesis). The size of the design space for these materials, the necessity to match relevant reaction conditions for efficacy, and the cost associated with running relevant experiments makes it an ideal test space to test capabilities of using frozen SOTA LLMs as useful surrogate models.

Extrapolating surface temperature fluctuations in response to the average $CO_2$ concentration variation over the last decade, at current carbon dioxide removal (CDR) efficiencies of 1.3 million tonnes of $CO_2$ per year, annual capture capacities must increase by a factor of 30, by 2030, to meet UN targets and avoid predicted catastrophic affects of global warming[2]. Thus, without significant economic incentive, the widespread adoption rate of necessary capture and conversion processes may not match the pace of greenhouse gas accumulation in the troposphere. Traditional catalytic discovery and deployment pathways have a record for broad implementation time-lines ranging from 5-40 years. This rate is unacceptable if the goal is to find materials that will allow us to avoid adverse affects due to tropospheric temperature fluctuations.

Although catalyst informatics is a rapidly growing field, due to its potential to increase efficiency in the material discovery process, a common challenge in developing models useful for structure-property approximations is the disproportionate availability of experimental data, relative to the size of the parameter design space. Using language as an agnostic feature space with BO may offer a solution to this issue as it could allow the unbiased use of material data for design and property prediction, while significantly reducing the cost of experiments needed to identify effective materials. In this setting, we can use BO-ICL to efficiently guide us through the complex design space of matching material and process design, by directly representing materials as standard operating procedures that includes important levers for both synthesizing and testing these materials.

## S1.1 Oxidative Coupling of Methane

This dataset focuses on catalysis optimization for the oxidative coupling of methane (Equation S1). Nguyen et al. [3] evaluated 12,708 experimental configurations across a range of parameters, including different catalyst active phases, support types, chemical compositions, reaction temperature, and reactant contact times. Nguyen et al. [3] reported the catalyst performance for $C_2$ (%) yield production under oxidative coupling of methane reaction conditions for 59 different catalysts, including reference materials. Catalyst performance was measured using a high-throughput screening instrument for consistent analyses, resulting in a high-fidelity dataset, ideal for early testing of BO-ICL. Reported tabulated conditions and results were converted to natural language representations (e.g. Figure S1)[4]. The property value distribution is visible in Figure S2 as a histogram, highlighting the sparsity in performance configurations above a 15% yield.

$$\text{(OCM)} \quad 2\mathrm{CH_4} + \mathrm{O_2} \rightarrow \mathrm{C_2H_4} + 2\mathrm{H_2O} \quad \Delta H_{rxn} = -280\,\text{kJ/mol} \tag{S1}$$

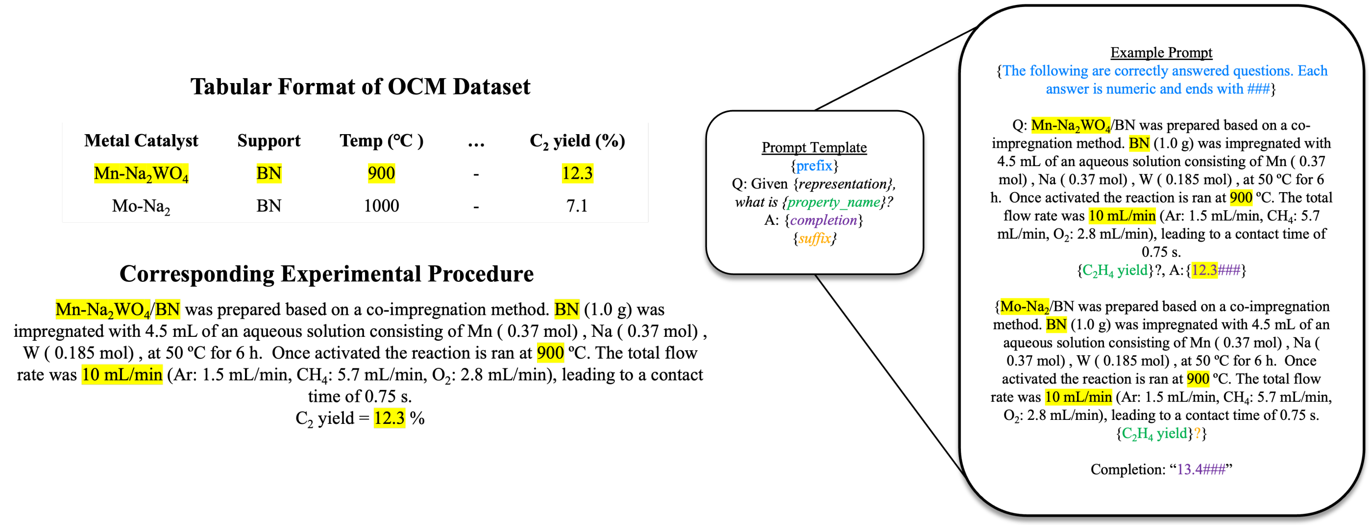


Figure S1: Example of original OCM tabular data and corresponding natural language representation used in BO-ICL evaluations. Additionally, a depiction of a prompt with $k = 1$, offering a single context example at inference.

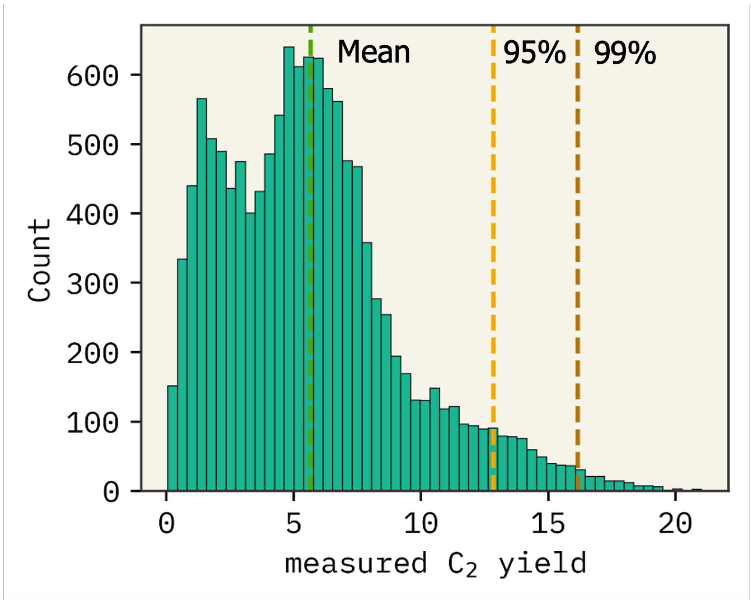


Figure S2: Histogram of OCM dataset performance distribution for $C_2$ yield (%) with annotated quantiles: Mean (green), $95^{th}$ percentile (orange), $99^{th}$ percentile (brown)

### S1.2 Solubility

The Estimated Solubility (ESOL)[5] dataset is a widely used benchmark in cheminformatics for predicting the aqueous solubility of small organic molecules. It consists of a collection of experimentally measured solubility values expressed in log molar units (logS). Originally, ESOL is published with the SMILES[6] representation of the molecules and the LogS values. This study used the PubChem API[7] to get IUPAC names. IUPAC names were input to our LLM models, and embedded representations of such names were used for the baselines.

### S1.3 Alloy Interface

This dataset was forged using data from [8], to calculate the charge transfer between two alloy interfaces modeled as a parallel plate capacitor. In this paper, an equilibrium Fermi-level $E'_f$ was used to analytically solve for the charge

transfer vector. Here, we leave it up to the BO-ICL to realize this relationship only given the Fermi level of the two alloys obtained separately from the materials project, the interface model (e.g. capacitor), and the transfer distance $d$, set as the sum of the largest van der Waals radii in each alloy. The log scaled distribution of charge transfer values and alloy combinations are visible in Figure S3.

$$\int_{E'_{f1}}^{E_{f1}} dE\, g_1(E) = \int_{E'_{f2}}^{E_{f2}} dE\, g_2(E) \tag{S2}$$

$$e\Delta n = \epsilon_0 \frac{E'_F}{d} \tag{S3}$$

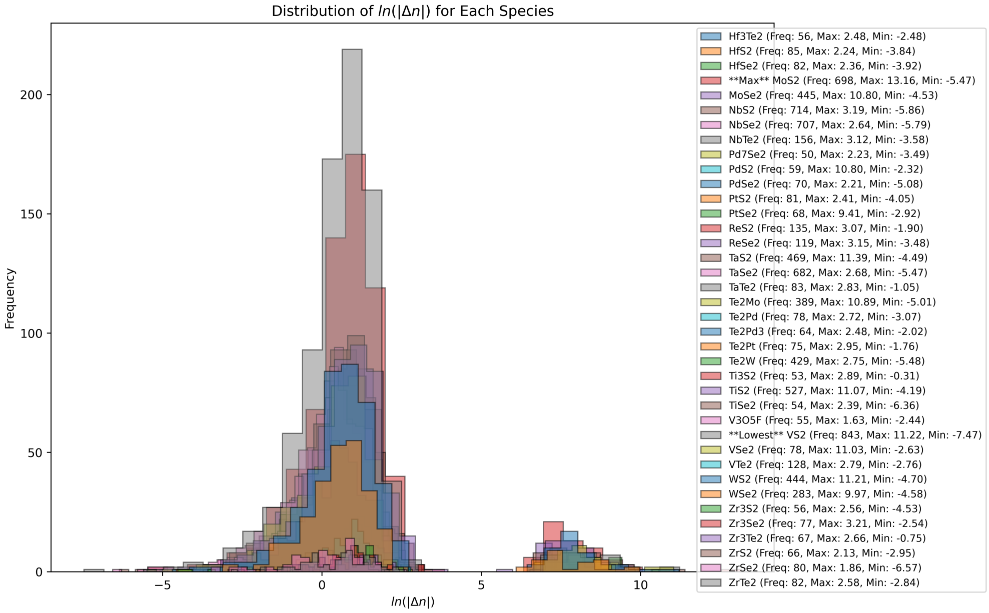


Figure S3: AII dataset distribution with color coded alloys and a range of their associated charge transfer values.

## S1.4 In-house RWGS

### S1.4.1 Experimental Pool with Pt

To further verify BO-ICL's application potential, we synthesized a custom pool of 3720 possible experiments aimed at identifying catalytic materials favorable for selective CO production under reverse water-gas shift (RWGS) reaction conditions. This experimental setup, which involves iterative human-led synthesis and reactor configuration, allows us to assess BO-ICL's performance when working with data that contains aleatoric uncertainty, typical of real-world catalysis studies. We constrained the material design space by limiting earth-abundant transition metal impregnation to 4.9 wt. % and .1 wt. % of platinum on supports that offer reaction-appropriate thermal stabilities and high surface area ($SiO_2/\gamma - Al_2O_3$)(Table S1). This sample space offers interesting considerations for such an application for reasons beyond $CO_2$ remediation solutions. For example, the choice of exploring equilibrium limited endothermic reaction offers an upper limit on $CO$ yield, governed by thermodynamics, and thus a clear termination policy is available. Further, the depth of available knowledge surrounding this reaction also allows us to introduce controlled complexities into the catalytic system. We know that incorporating low concentrations of precious metals like Pt can rapidly dissociate $H_2$ due to the insignificant activation barrier for $H_2$ adsorption on Pt metal surfaces [9]. By introducing Pt, we can further shift the focus toward optimizing selective $CO_2$ activation chemistries, by eliminating concerns of active hydrogen availability. This shift amplifies the need for finer process parameter tuning to match favorable synergistic binding energies that will resist deep reduction into thermodynamically favorable, and undesired, methane formation.

Table S1: Reverse Water-Gas Shift Design Space: 3,720

| Category | Specification |
|---|---|
| Active metals | Bimetallics of Fe/Co/Ni/Cu/Zn with Pt for trimetallics, plus monometallic controls |
| Supports | $SiO_2/Al_2O_3$ |
| Pretreatment | Calcination / Reduction / Inert |
| Temperature | 275-325 °C |
| GHSV | 1,000-50,000 mL $g^{-1}$ $h^{-1}$ |

#### S1.4.2 Trimetallic Exclusive Pool without Pt

In the initial RWGS experimental pool, cost, safety, and stability constraints were intentionally relaxed to enable early method evaluation; for example, Pt appeared in candidate formulations to facilitate $H_2$ activation and bias optimization toward $CO_2$ activation rather than $H_2$ availability. In the expanded trimetallic design space (Table S1), we increase system complexity while restricting candidates to comparatively earth-abundant, non-toxic transition metals (W/Mo/Zr/Zn/Mn/V) with optional K promotion, and we explicitly exclude highly regulated/toxic elements (e.g., Cd, Pb, Cr(VI)) and expensive noble metals. We additionally omit metals that are strongly represented in RWGS literature and commonly active for $CO_2$ conversion (Fe, Cu, Ni) to encourage exploration of less-studied composition space. Candidate supports are restricted to field-relevant oxides ($SiO_2$, $Al_2O_3$, $CeO_2$, $ZrO_2$, $TiO_2$), and pretreatments span oxidizing (air calcination), reducing (CO or $H_2$), and inert conditions (Table S1).

To parameterize trimetallic composition continuously while maintaining practical synthesis constraints, we define metal ratios by sampling points on a ternary simplex (schematically illustrated in Figure S4). Each sampled point corresponds to a unique molar-fraction triplet for a chosen metal set (A/B/C), enabling broad coverage of composition space without enumerating an infeasibly fine grid. This ternary exploration is motivated by prior empirical studies that map catalytic performance over trimetallic composition space (Figure S4, inset), which often reveal non-convex "islands" of high activity surrounded by low-performing regions.

To implicitly probe operational stability under a fixed protocol, each catalyst is evaluated for a minimum of 15 h following pretreatment, and the CO yield objective is computed from the final 3 h of the experiment. However, this fixed time-on-stream evaluation also exposes a practical limitation in this setting: several high-scoring candidates exhibit extended induction behavior and may not reach steady state within the 15 h window, which can underestimate true steady-state performance and complicate fair comparison across candidates. One representative case shows a monotonic increase in conversion/yield over time without clear equilibration within the campaign horizon (Figure **??**). Consistent with this, BO trajectories can preferentially select candidates that exhibit reliable nonzero activity within the fixed evaluation window, even when peak performance may occur later.

Table S2: Reverse Water-Gas Shift Trimetallic Design Space: 360,000

| Category | Specification |
|---|---|
| Active metals | Trimetallics of W/Mo/Zr/Zn/Mn/V and with-/without K promoter |
| Supports | $SiO_2/Al_2O_3/CeO_2/ZrO_2/TiO_2$ |
| Pretreatment | Calcination 450 °C (Air) / Reduction 300-600 °C (CO, $H_2$) / Inert 110 °C (Ar) |
| Temperature | 300 °C |
| GHSV | 36,000 mL $g^{-1}$ $h^{-1}$ |
| Metal Ratios | 250 possible combinations (Figure S4) |

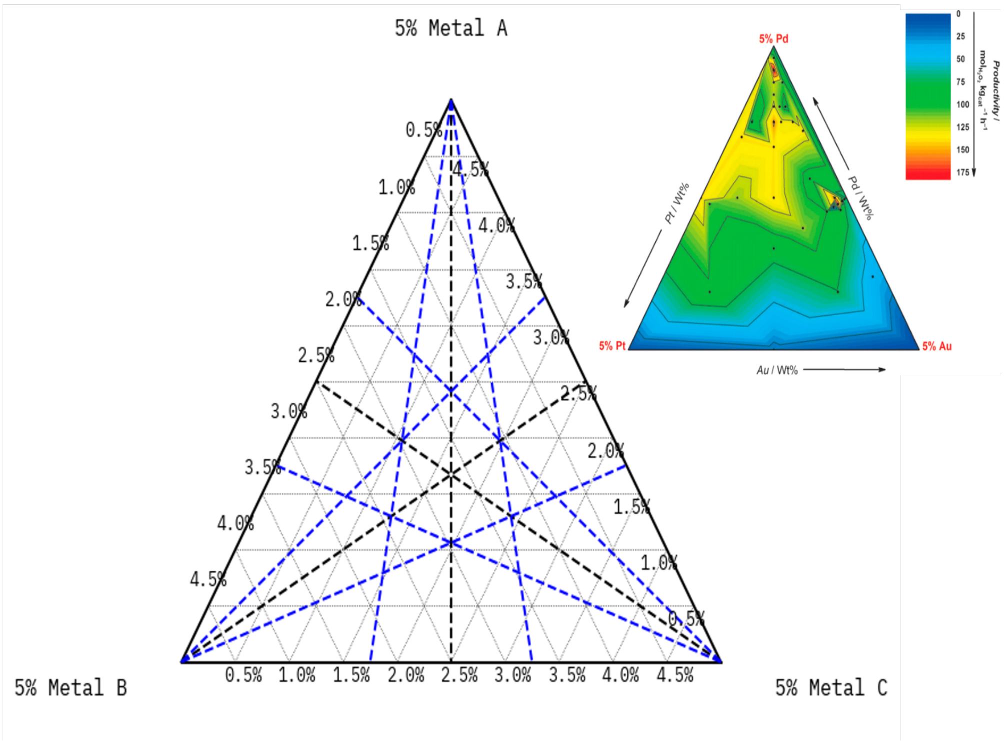


Figure S4: Schematic of trimetallic composition sampling used to generate metal-ratio candidates in the expanded RWGS design space. Points on the ternary simplex correspond to unique molar-fraction triplets for three metals (A/B/C) at fixed total loading, enabling broad coverage of composition space via random sampling along feasible ratio paths. Inset: representative literature ternary performance map illustrating that trimetallic catalyst landscapes can exhibit localized "islands" of high activity within a largely low-performing composition space (shown here for an example Pd-Pt-Au system) [10]

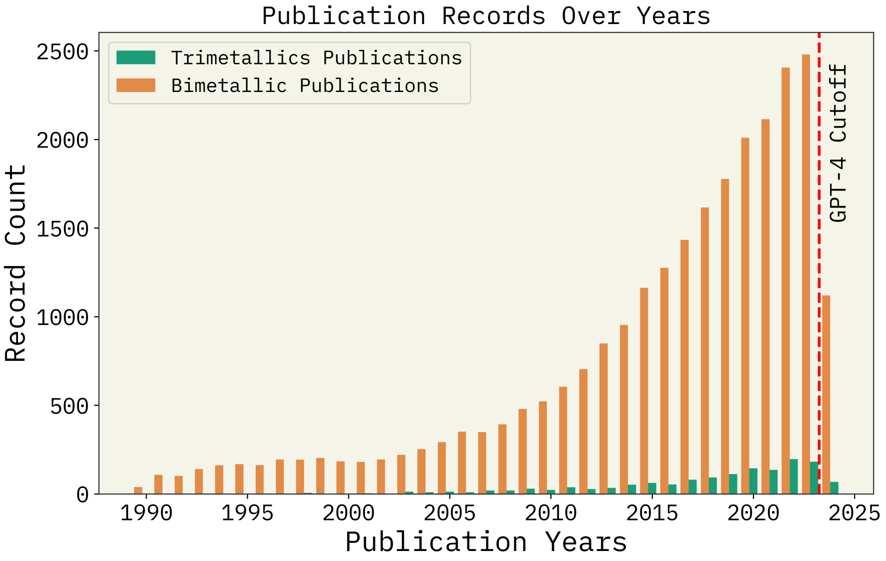


Figure S5: Publication record by year comparing bimetallic (orange) versus trimetallic (green) RWGS-related catalyst studies. Trimetallic reports remain substantially less common across the full time span. The red dashed line marks the GPT-4 knowledge-cutoff year used in this work.

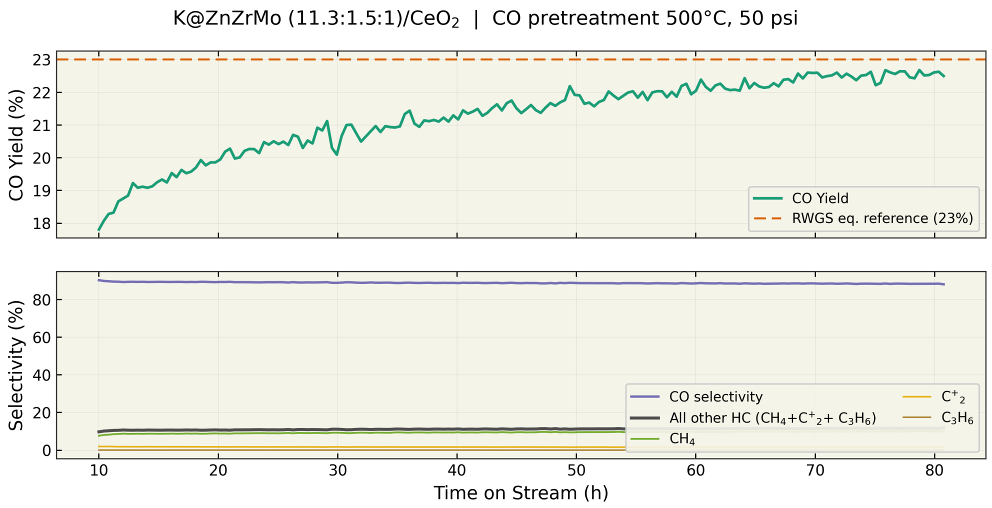


Figure S6: Time-on-stream RWGS performance of K@ZnZrMo(11.3:1.5:1)/$CeO_2$ after CO pretreatment at 500 °C under 50 psi. The upper panel shows CO yield versus time on stream, while the lower panel shows selectivities to CO, $CH_4$, $C_2^+$, $C_3H_6$, and total non-CO hydrocarbons. The dashed orange line marks the RWGS equilibrium reference for CO yield (23%).

Table S3: (CO Yield$_{max}$ = 23%): GPT-4o-EI+Greedy and GPT-4.1-EI+Greedy trajectories vs. random selection.

| Loop Iter. | Trimetallics | Pre-treat | Ratio | Yield$_{CO}$ (%) |
|---|---|---|---|---|
| i1 | K@Zn-Zr-Mo/$ZrO_2$ | CO \| 300 | 1.5 \| 1.0 \| 6.7 | 3.30 |
| i2 | Zn-Mn-V/$TiO_2$ | Ar \| 110 | 50.0 \| 1.0 \| 4.0 | 2.58 |
| 3 | W-Mn-V/$SiO_2$ | $H_2$ \| 350 | 1.0 \| 3.0 \| 1.5 | 3.03 |
| 4 | K@Mn-W-Mo/$CeO_2$ | CO \| 350 | 1.5 \| 1.0 \| 1.4 | 3.85 |
| 5 | K@Zn-W-Mo/$Al_2O_3$ | $H_2$ \| 600 | 1.5 \| 1.0 \| 1.4 | 2.68 |
| 6 | Zn-Mo-Mn/$Al_2O_3$ | $H_2$ \| 300 | 4.4 \| 1.5 \| 1.0 | 2.87 |
| 7 | K@Zn-Zr-Mo/$TiO_2$ | $H_2$ \| 400 | 1.0 \| 13.3 \| 4.0 | 4.16 |
| 8 | Zn-Mo-Mn/$TiO_2$ | $H_2$ \| 550 | 3.0 \| 1.5 \| 1.0 | 2.60 |
| 9 | K@Zn-Mo-V/$Al_2O_3$ | CO \| 600 | 4.0 \| 8.8 \| 1.0 | 0.21 |
| 10 | K@Zn-Zr-Mo/$Al_2O_3$ | CO \| 450 | 11.3 \| 1.5 \| 1.0 | 17.20 |

(a) Trajectory 1: GPT-4o-EI+Greedy

| Loop Iter. | Trimetallics | Pre-treat | Ratio | Yield$_{CO}$ (%) |
|---|---|---|---|---|
| i1 | K@Zn-Zr-Mo/$ZrO_2$ | CO \| 300 | 1.5 \| 1.0 \| 6.7 | 3.30 |
| i2 | Zn-Mn-V/$TiO_2$ | Ar \| 110 | 50.0 \| 1.0 \| 4.0 | 2.58 |
| 3 | W-Mn-V/$SiO_2$ | $H_2$ \| 350 | 1.0 \| 3.0 \| 1.5 | 0.34 |
| 4 | K@Mn-W-Mo/$CeO_2$ | CO \| 350 | 1.5 \| 1.0 \| 1.4 | 1.90 |
| 5 | K@Zn-W-Mo/$CeO_2$ | CO \| 600 | 2.1 \| 3.0 \| 1.5 | 7.60 |
| 6 | K@Zn-Mo-Mn/$CeO_2$ | CO \| 550 | 1.5 \| 2.1 \| 1.0 | 4.00 |
| 7 | K@Zn-Mo-V/$CeO_2$ | CO \| 600 | 25.0 \| 1.0 \| 1.5 | 2.30 |
| 8 | K@Zn-Zr-Mo/$TiO_2$ | CO \| 450 | 8.0 \| 1.0 \| 2.0 | 0.58 |
| 9 | K@Zn-Mo-Mn/$CeO_2$ | CO \| 550 | 50.0 \| 1.0 \| 4.0 | 4.80 |
| 10 | K@Zn-Zr-Mo/$CeO_2$ | CO \| 500 | 11.3 \| 1.5 \| 1.0 | 18.00 |

(b) Trajectory 2: GPT-4.1-EI+Greedy

| Loop Iter. | Trimetallics | Pre-treat | Ratio | Yield$_{CO}$ (%) |
|---|---|---|---|---|
| i1 | K@Zn-Zr-Mo/$ZrO_2$ | CO \| 300 | 1.5 \| 1.0 \| 6.7 | 3.30 |
| i2 | Zr-Mn-V/$TiO_2$ | Ar \| 110 | 50.0 \| 1.0 \| 4.0 | 2.58 |
| 3 | K@Zr-Zn-V/$CeO_2$ | CO \| 350 | 1.0 \| 1.5 \| 11.3 | 10.30 |
| 4 | K@Zn-Mn-V/$Al_2O_3$ | CO \| 400 | 1.0 \| 4.0 \| 8.8 | 1.34 |
| 5 | K@Mo-Mn-V/$SiO_2$ | CO \| 600 | 1.4 \| 1.0 \| 1.5 | 2.10 |
| 6 | K@Zn-Mn-V/$SiO_2$ | Ar \| 110 | 1.0 \| 4.0 \| 6.0 | 1.95 |
| 7 | K@Zn-Mn-V/$CeO_2$ | $H_2$ \| 500 | 6.0 \| 1.0 \| 4.0 | 2.38 |
| 8 | K@Zn-Zr-Mo/$Al_2O_3$ | CO \| 300 | 1.5 \| 6.7 \| 1.0 | 2.02 |
| 9 | Zr-Mo-V/$CeO_2$ | CO \| 450 | 6.0 \| 1.0 \| 4.0 | 0.14 |
| 10 | K@Zr-Mo-W/$SiO_2$ | CO \| 300 | 2.1 \| 1.0 \| 1.5 | 3.90 |

(c) Random

#### S1.4.3 Thermodynamic upper limit approximation for $CO_{yield}$:

$$\text{(RWGS)} \quad CO_2 + H_2 \rightleftharpoons CO + H_2O \tag{S4}$$

| | $CO_2$ | $3H_2$ | $CO$ | $H_2O$ |
|---|---|---|---|---|
| **Initial** | $n_0$ | $3n_0$ | - | - |
| **Change** | $-n_0x$ | $-n_0x$ | $+n_0x$ | $+n_0x$ |
| **Equilibrium** | $-n_0(1-x)$ | $-n_0(3-x)$ | $n_0x$ | $n_0x$ |

Here, $n_0$ represents the initial moles of $CO_2$, $x$ denotes the conversion of $CO_2$, and $K_{\text{eq}}$ is the equilibrium constant.

$$K_{\text{eq}} = \frac{[\text{CO}][\text{H}_2\text{O}]}{[\text{CO}_2][\text{H}_2]} = \frac{x^2}{(1-x)(3-x)} = \exp\left(-\frac{\Delta G^\circ}{RT}\right) \tag{S5}$$

Where: $\Delta G^\circ$ represents the standard Gibbs free energy change (J/mol), $R$ is the universal gas constant (8.314 J/mol·K), and $T$ means the Temperature in Kelvin (K).

At equilibrium, at $T = 325\,^\circ\text{C}$ and $P = 1\,\text{atm}$, the conversion of $CO_2$, denoted as $x_{\text{CO}_2}$, is approximately 26%.

The yield of CO can be calculated as:

$$\text{CO}_{\text{yield}} = x_{\text{CO}_2} \cdot S_{\text{CO}_2} \tag{S6}$$

Where: $x_{\text{CO}_2}$ is the conversion of $CO_2$, defined as the fraction of $CO_2$ reacted, calculated as:

$$x_{\text{CO}_2} = \frac{\text{CO}_2^{\text{in}} - \text{CO}_2^{\text{out}}}{\text{CO}_2^{\text{in}}} \cdot 100\% \tag{S7}$$

$S_{\text{CO}_2}$ is the selectivity for CO formation, defined as the ratio of the moles of CO formed to the moles of $CO_2$ consumed:

$$S_{\text{CO}_2} = \frac{\text{CO}^{\text{out}}}{\text{CO}_2^{\text{in}} - \text{CO}_2^{\text{out}}} \cdot 100\%. \tag{S8}$$

#### S1.4.4 Catalyst Synthesis and on Dataset

For synthesizing materials for the RWGS dateset, respective nitrate precursors were first proportionally and separately dissolved in Mill Q water for Fe, Co, Ni, Cu, Zn, and Pt metals. Then the appropriate concentrations of dissolved metal precursors were mix into a single beaker to ensure a total loading of 5 % wt. of metal loading with respect to both the weight of the support and transition metals. We then add alloy solution drop-wise to the selected support using the incipient wetness impregnation synthesis method. The impregnated catalyst was then dried, by ramping the system temperature to 90 °C at 1 °C per minute with hold time of 4 hours for gentle water removal. Following this, another temperature ramp at the same rate to 450 °C and calcined, when called for, in air for 4 hours.

#### S1.4.5 Reactor Tests

Each catalyst was loaded into a stainless-steel reactor (outer diameter: 6.35 mm; inner diameter: 4.57 mm; reactor length: 40 cm). For reduction, 40 mL/min of $H_2$ was introduced for 2 hours at 450 °C and 50 psig, or alternatively, the catalyst was degassed under 20 mL/min of Ar for 2 hours. Following reduction or degassing, the reactor was isolated, and bypass effluents, under 14.7 psig of pressure, were analyzed to establish a baseline. The gas composition for the reverse water-gas shift (RWGS) reaction consisted of 10 mL/min $CO_2$, 30 mL/min $H_2$, and 5 mL/min Ar, resulting in a $H_2/CO_2$ ratio of 3:1. Catalyst mass loadings were varied to achieve gas hourly space velocities (GHSV) ranging from 1,000 to 50,000 mL/g/h.

The Gas Hourly Space Velocity (GHSV) is calculated as:

$$\text{GHSV} = \frac{F}{V_c} \tag{S9}$$

where $F$ is the volumetric flow rate of gas (e.g., in mL/h), and $V_c$ is the estimated catalyst bed volume. Isothermal reactions were run for 8 h. Effluent reactor concentrations were analyzed by an in-line Agilent Technologies 7890B GC system equipped with a flame ionization detector (FID) and a thermal conductivity detector (TCD). The concentration

of each gas phase species was calibrated by correlating the peak area of the pure compound to its concentration in a calibration gas standard. For all reactor experiments, the carbon balance closes to 100% ± 2%.

## S2 BO-ICL algorithms

**Algorithm S1** BO-ICL algorithm

**Initialize:**
  Iteration counter: $t \leftarrow 0$
  Labeled dataset: $\mathcal{L} \leftarrow \{(x_n, y_n)|n \in 1, 2, ..., n_{\mathcal{L}}\}$
  Unlabeled dataset: $\mathcal{U} \leftarrow \{(A_n)|n \in 1, 2, ..., n_{\mathcal{U}}\}$
  Size of the SubPool: size
  Size of the context: k
**Input:**
  Surrogate function: $f(x)$ *# A LLM with long-term memory $\mathcal{M}$*
  Acquisition function: $\alpha(x)$
**function** BO-ICL($f(x)$, $\alpha(x)$)
  $\mathcal{M}_t \leftarrow \mathcal{L}$
  **while** stopping criterion not met **do**
    $t = t + 1$
    $(x^+, y^+) = (\arg\max_{\mathcal{M}}\{y_n\}, max_{\mathcal{M}}\{y\})$
    best $\leftarrow (x^+, y^+)$

    ***# Select next point:***
    reference $\leftarrow$ LLM($y^+$, k, $\mathcal{M}_{t-1}$) *# Inverse design experiment generation. See Algorithm S3*
    SubPool $\leftarrow$ CreateSubPool($\mathcal{U}$, reference, size) *# See Algorithm S4*
    $r_{x,t} \leftarrow \arg\max_{SubPool} \alpha(LLM(SubPool.x, k, \mathcal{M}_{t-1}))$ *# Inference prediction generation. See Algorithm S3*

    ***# Evaluate objective:***
    $r_{y,t} \leftarrow$ Label obtained from the experiment
    **if** $r_{y,t} >$ best.$r_y$ **then**
      best $\leftarrow (r_x, r_y) = (r_{x,t}, r_{y,t})$
    **end if**

    ***# Update LLM's memory:***
    $\mathcal{M}_t = \mathcal{M}_{t-1} \cup \{(r_{x,t}, r_{y,t})\}$
  **end while**
  **Output:** best *# Return best point point found*
**end function**

**Algorithm S2** Algorithm to compute MMR. CosSim is cosine similarity.

**Initalize:**
    penalty ← 0
**Input:**
    Element for which MMR is being computed: $e$
    Reference element: $r$
    Already selected elements: $S$
**function** COMPUTEMMR($e$, $r$, $S$)
  **for** s in S **do**
    curr_penalty ← CosSim($r$, $s$)
    **if** curr_penalty > penalty **then**
      penalty ← curr_penalty
    **end if**
  **end for**
  mmr_score ← $\lambda$CosSim($r$, $e$) - $(1 - \lambda)$penalty
  **return** mmr_score
**end function**

**Algorithm S3** LLM prediction algorithm

**Initialize:**
    context examples: K ← []
    LLM provider: api
    Task description: prefix
**Input:**
    LLM's memory:: $\mathcal{M} \leftarrow \{(\eta_x, \eta_y)_n | n \in 1, 2, ..., n_{\mathcal{M}}\}$
    context size: $k$
    Element for which the prediction needs to be done: $input$
**function** LLM($input$, $k$, $\mathcal{M}$)
  *# Create context for ICL*
  **while** length(K) < k **do**
    new_example ← $\arg\max_L$ ComputeMMR($\mathcal{M}$, input, K)
    K.append(new_example)
  **end while**

  *# Prepare the propmt*
  query ← prefix + examples(K) + suffix(input)

  *# Send a request to the LLM provider*
  completion ← api.request(query)
  **return** completion
**end function**

**Algorithm S4** Sub-Pool creation algorithm

```
Initialize:
    counter: k ← 0
    SubPool ← []                                                    # Empty list
Input:
    Pool of unlabaled data: 𝒰 ← {(A_n)|n ∈ 1, 2, ..., n_U}
    Reference element: r
    Requested number of elements in the Sub-Pool: size
function CreateSubPool(U, r, size)
    while k < size do
        mmr_score ← 0
        sim_element ← ""
        for α in U do
            curr_mmr_score ← ComputeMMR(α, r, SubPool)
            if curr_mmr_score > mmr_score then
                sim_element ← α
                mmr_score ← curr_mmr_score
            end if
        end for
        k ← k + 1
        SubPool.append(sim_element)
    end while
    return SubPool
end function
```

# S3 Baselines

## S3.1 Analytical random

All Bayesian optimization plots show $y_N^*$ – the current best at sample count $N$. The random baseline was estimated via a quantiling of the data points. Namely, for random sampling $y_N^*$ is estimated with:

$$E[y_N^*] = \sum_i^K y_i \mathrm{P}(s_m = y_i) \; ; \; s_m = \max(y_1, y_2, \ldots, y_N)$$
$$\approx \sum_j^Q \left(j^N - (j-1)^N\right) \left(\frac{1}{Q}\right)^N q_j \tag{S10}$$

where $q_i$ is the $i$th quantile of $y$ (out of $Q$) and $K$ is the number of datapoints in the pool.

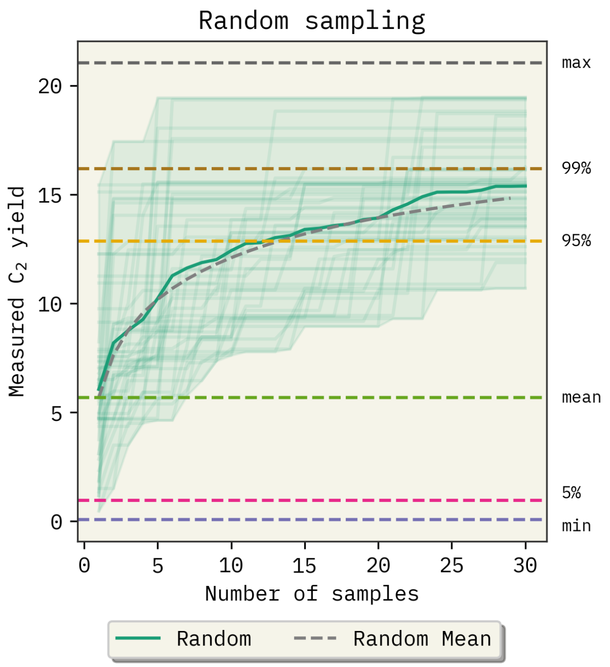


Figure S7: Comparison between a large number of random sampled trajectories with the analytical derived expected value of a random sampling. The analytical values are shown as a gray dashed line while the random sample is shown in green. Each independent trajectory is shown as shaded green lines and the average is displayed as the bold green line. Within 50 independent runs, the average of the random sampling converges to the analytical curve.

To ensure our modeling was correct, we proof case this equation by comparing it with the average of several independent random sampled trajectories. Figure S7 shows a plot with both the analytical and the average of 50 random runs. As expected, the expected values for each number of samples are almost identical.

### S3.2 OCM dataset with no true correlation

To demonstrate that the correlation between the chemical information in the input paragraphs and the target values is essential for optimization using BO-ICL, we artificially corrupted the OCM dataset. Specifically, we used kernel density estimation (via gaussian_kde) to approximate the probability distribution of the original labels, as shown in. From this estimated distribution, we sampled a new set of labels with the same cardinality as the original dataset. These sampled labels were then randomly shuffled to preserve the overall distribution while eliminating any potential correlation between the input procedural features and the labels. This process yields a randomized label set that retains the original distribution's shape, visible in Figure S8, but has no functional relationship to the inputs. Figure S9 shows that upon corrupting the dataset, BO-ICL with `gpt-4o` can only perform equally to random sampling the dataset.

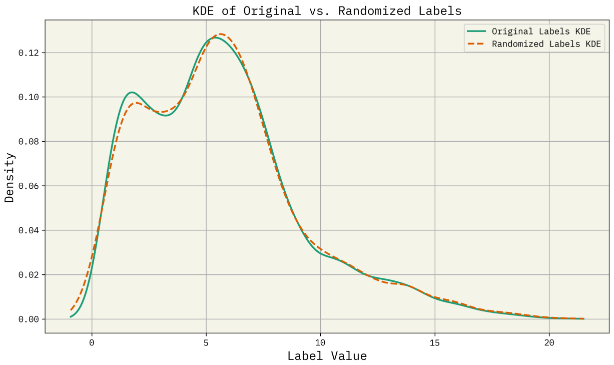


Figure S8: Comparison of probability distributions between the original and randomized OCM labels using Gaussian KDE. The randomized labels were generated by sampling from the KDE-fitted distribution of the original labels and then shuffling the samples. This preserves the overall label distribution while removing any correlation with the input features.

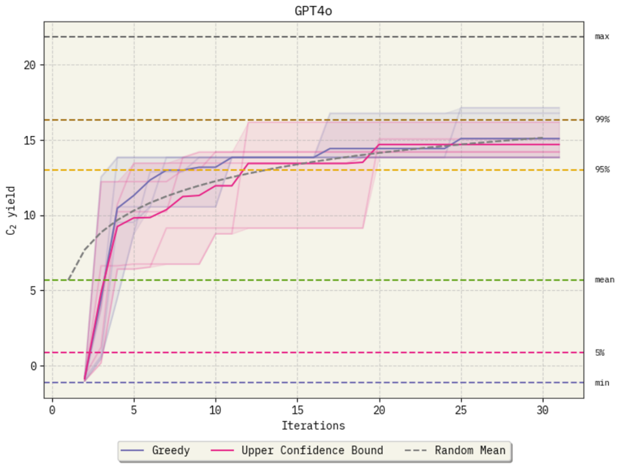


Figure S9: BO-ICL trajectory on corrupted OCM data. This experiment shows that without a true correlation between the input experimental procedure, BO-ICL will be ineffective in guiding optimization within the design space.

### S3.3 BO dependence control experiment using OCM dataset

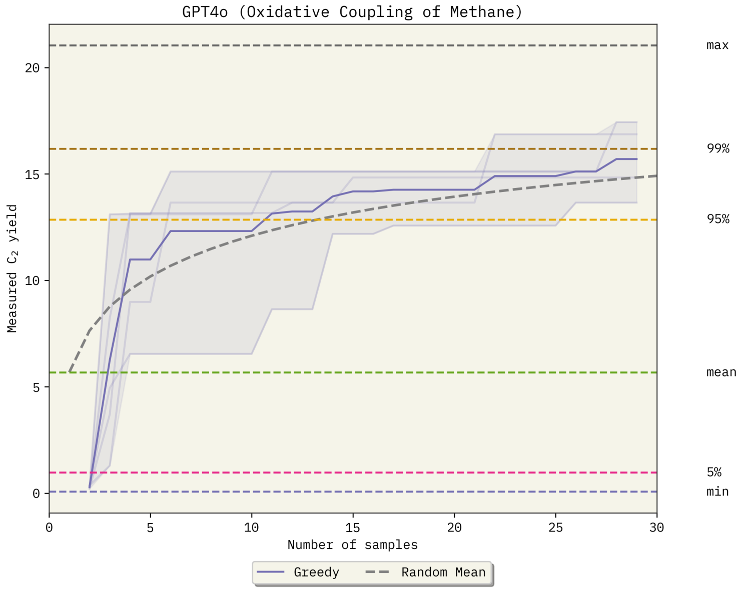


Figure S10: BO-ICL performance on the OCM dataset with omission of the BO steps during inverse design. With the inversely inferred procedure that GPT-4o generates, we use the embedded representation as a reference in the cosine similarity algorithm to find the closest available procedure in the unlabeled pool to evaluate and add a new available context point. This experiment serves as a control to highlight the reliance on BO to achieve satisfactory performance.

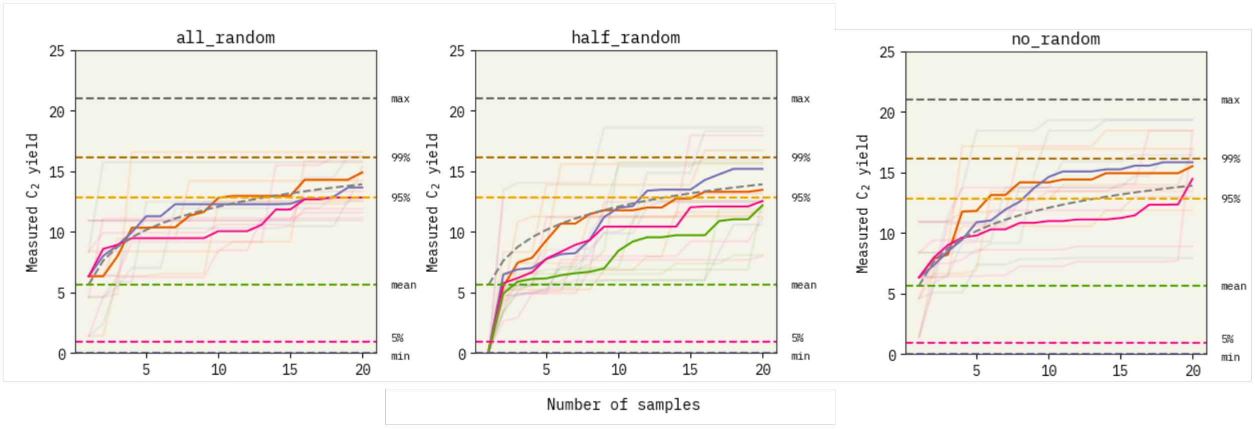


Figure S11: Early ablation study of subpool population strategies on the OCM dataset using `davinci-300`. Fully random (`all_random`) and partially random (`half_random`) subpool construction are compared with a non-random approach (`no_random`). Solid lines show median performance across runs, and lighter traces indicate individual trajectories. Acquisition functions are Expected Improvement (orange), Greedy (purple), and UCB with a modified exploration parameter (pink). Horizontal dashed lines indicate dataset statistics (minimum, mean, upper percentiles, and maximum). Increasing reliance on random subpool population is associated with reduced optimization performance, highlighting the importance of structured subpool construction.

In this context, the result obtained using inverse design alone (Figure S10) should be interpreted narrowly: a trajectory based entirely on inverse design can in some cases resemble a random walk, but this does not imply that inverse-design-guided subpool construction is uninformative. Rather, it suggests that inverse design alone may be insufficient to fully control exploration. One plausible interpretation is that inverse design contributes useful directional bias toward promising regions, while BO refines and corrects those proposals through local search. This may be especially relevant in catalysis settings, where the model can capture broad semantic relationships among catalyst compositions and processing conditions, yet remain less sensitive to small numerical variations that can strongly affect chemical behavior.

Together, these figures suggest that subpool construction strategy materially affects downstream BO performance. In early ablation experiments with `davinci-003` on the OCM dataset, fully random (`all_random`) and partially random (`half_random`) subpool population both tended to underperform relative to the structured (`no_random`) strategy (Figure S11). This trend suggests that increasing reliance on random subpool population may reduce the quality of the candidate set presented to BO, particularly in early iterations, when coverage and diversity are most important.

### S3.4 k-Nearest neighbor

The k-Nearest Neighbors (KNN) model uses a space of embedded strings to perform the similarity search. It stores embeddings using the OpenAI embed model `text-ada-embedding-002` computed from the string with the experimental procedure description. For inference, it retrieves $k$ experiments with the greatest cosine similarity score from this saved space. The prediction is then the average of the $k$ retrieved experiments.

### S3.5 Kernel ridge regression

The kernel ridge regression (KRR)[11, 12] was implemented from scratch using numpy. The goal of KRR is to minimize the following loss function:

$$L((\boldsymbol{x}, y)|w) = \sum_{i=1}^{n} \left(y_i - w^T x_i\right)^2 + \lambda ||w||_2 \tag{S11}$$

In our implementation, we compute the kernel trick as

$$\boldsymbol{K} = k(x_i, x_j) = \phi(x_i)^T * \phi(x_j) \tag{S12}$$

where, the function $\phi$ is the embedding function. In our application, the embeddings are normalized after calculation. The training involves solving the linear equation for $\alpha$, where $\alpha$ is the tensor of coefficients for the model.

$$y = \boldsymbol{K}\alpha + \lambda\alpha \tag{S13}$$

On inference time, the following equation is computed.

$$\hat{y} = \sum_{i=1}^{n} \alpha\phi(x_i)^T \phi(x'_j) \tag{S14}$$

### S3.6 Gaussian process

Gaussian Process (GP) is a probabilistic model often used to model the unknown objective function $f$ on a Bayesian optimization. The GP is a non-parametric model that defines a prior distribution over functions. Detailed discussion of GPs can be found in Rasmussen and Williams [13] and Frazier [14]. Briefly, given a set of observations $\mathcal{D} = \{(x_i, y_i)\}_{i=1}^{n}$, where $y_i = f(x_i) + \epsilon$ and $\epsilon \sim \mathcal{N}(0, \sigma^2)$ represents noise in the function evaluations, the GP models the objective function as:

$$f(x) \sim \mathcal{GP}(\mu(x), k(x, x')) \tag{S15}$$

where $\mu(x)$ is the mean function and $k(x, x')$ is the covariance function or kernel, encoding the similarity between points $x$ and $x'$.

The posterior distribution of the function at any new point $x_*$, after observing data $\mathcal{D}$, is given by:

$$\mu_*(x_*) = \mu(x_*) + k(x_*, X)K^{-1}(y - \mu(X)) \tag{S16}$$

$$\sigma_*^2(x_*) = k(x_*, x_*) - k(x_*, X)K^{-1}k(X, x_*) \tag{S17}$$

where $X$ is the set of observed points, $K$ is the covariance matrix with elements $K_{ij} = k(x_i, x_j)$, and $k(x_*, X)$ is the vector of covariances between the new point $x_*$ and the observed points $X$.

In this study, GP was implemented using the bo-torch[15] package. More specifically, we used the `SingleTaskGP` as the regressor, which uses the Matérn-5/2 kernel. To prepare the data, we computed embeddings using the OpenAI embed model `text-ada-embedding-002`. Ada embeddings is a vector of 1532 dimensions. To simplify the GP training, we used an isomap to reduce the input dimension from 1532 to 32.

## S4 Additional results

Table S4: Statistical tests

| Dataset | Model | T-test | p-value |
|---|---|---|---|
| ESOL | gpt-3.5-turbo-0125 | k=1 ↔ k=2 | 0.01719 |
| | gpt-3.5-turbo-0125 | k=2 ↔ k=5 | 0.19953 |
| | gpt-3.5-turbo-0125 | k=5 ↔ k=10 | 0.02413 |
| | gpt-3.5-turbo-0125 | T=0.05 ↔ T=0.1 | 0.04135 |
| | gpt-3.5-turbo-0125 | T=0.1 ↔ T=0.5 | 0.15216 |
| | gpt-3.5-turbo-0125 | T=0.5 ↔ T=0.7 | 0.00596 |
| | gpt-3.5-turbo-0125 | T=0.7 ↔ T=1.0 | 0.58761 |
| | gpt-3.5-turbo-0125 | T=1.0 ↔ T=1.5 | 0.04197 |
| | gpt-3.5-turbo-0125 | N=1 ↔ N=5 | 0.29242 |
| | gpt-3.5-turbo-0125 | N=5 ↔ N=10 | 0.06123 |
| | gpt-3.5-turbo-0125 | N=10 ↔ N=25 | 0.41665 |
| | gpt-3.5-turbo-0125 | N=25 ↔ N=50 | 0.00475 |
| | gpt-3.5-turbo-0125 | N=50 ↔ N=100 | 0.35680 |
| | gpt-3.5-turbo-0125 | N=100 ↔ N=250 | 0.02949 |
| | gpt-3.5-turbo-0125 | N=250 ↔ N=500 | 0.03278 |
| | gpt-3.5-turbo-0125 | N=500 ↔ N=1k | 0.03216 |
| OCM | gpt-3.5-turbo-0125 | k=1 ↔ k=2 | 0.01196 |
| | gpt-3.5-turbo-0125 | k=2 ↔ k=5 | 0.19324 |
| | gpt-3.5-turbo-0125 | k=5 ↔ k=10 | 0.98559 |
| | gpt-3.5-turbo-0125 | T=0.05 ↔ T=0.1 | 0.01115 |
| | gpt-3.5-turbo-0125 | T=0.1 ↔ T=0.5 | 0.13083 |
| | gpt-3.5-turbo-0125 | T=0.5 ↔ T=0.7 | 0.10318 |
| | gpt-3.5-turbo-0125 | T=0.7 ↔ T=1.0 | 0.61822 |
| | gpt-3.5-turbo-0125 | T=1.0 ↔ T=1.5 | 0.01191 |
| | gpt-3.5-turbo-0125 | N=1 ↔ N=5 | 0.18649 |
| | gpt-3.5-turbo-0125 | N=5 ↔ N=10 | 0.02414 |
| | gpt-3.5-turbo-0125 | N=10 ↔ N=25 | 0.02752 |
| | gpt-3.5-turbo-0125 | N=25 ↔ N=50 | 0.95171 |
| | gpt-3.5-turbo-0125 | N=50 ↔ N=100 | 0.16021 |
| | gpt-3.5-turbo-0125 | N=100 ↔ N=250 | 0.31569 |
| | gpt-3.5-turbo-0125 | N=250 ↔ N=500 | 0.14796 |
| | gpt-3.5-turbo-0125 | N=500 ↔ N=1k | 0.00409 |

Table S5: Regression metrics. The best value found for each metric is highlighted in **bold** while the second best is underlined.

| Dataset | Model | N | k | T | MAE (↓) | corr (↑) | nll (↓) |
|---|---|---|---|---|---|---|---|
| Solubility | gpt-3.5-turbo-0125 | 700 | 1 | 0.05 | 1.120 ± 0.039 | 0.664 ± 0.043 | 7898.124 ± 3097.894 |
| | gpt-3.5-turbo-0125 | 700 | 2 | 0.05 | 0.961 ± 0.099 | 0.751 ± 0.044 | 5543.792 ± 3076.489 |
| | gpt-3.5-turbo-0125 | 700 | 5 | 0.05 | 0.880 ± 0.061 | 0.719 ± 0.031 | 11614.484 ± 10155.578 |
| | gpt-3.5-turbo-0125 | 700 | 10 | 0.05 | 0.779 ± 0.040 | 0.797 ± 0.041 | 2575.350 ± 2685.093 |
| | gpt-3.5-turbo-0125 | 700 | 5 | 0.01 | 0.872 ± 0.059 | 0.729 ± 0.059 | 3886.785 ± 4475.754 |
| | gpt-3.5-turbo-0125 | 700 | 5 | 0.1 | 0.861 ± 0.055 | 0.748 ± 0.040 | 1302.400 ± 318.958 |
| | gpt-3.5-turbo-0125 | 700 | 5 | 0.5 | 0.802 ± 0.050 | 0.796 ± 0.035 | 125.730 ± 137.078 |
| | gpt-3.5-turbo-0125 | 700 | 5 | 1.0 | 0.898 ± 0.045 | 0.758 ± 0.074 | 165.623 ± 163.004 |
| | gpt-3.5-turbo-0125 | 700 | 5 | 1.5 | 3.055 ± 1.783 | 0.230 ± 0.288 | 43.270 ± 0.430 |
| | gpt-3.5-turbo-0125 | 1 | 5 | 0.7 | 2.794 ± 0.469 | 0.050 ± 0.214 | 215.484 ± 187.379 |
| | gpt-3.5-turbo-0125 | 5 | 5 | 0.7 | 3.189 ± 0.520 | -0.042 ± 0.179 | 200.413 ± 97.635 |
| | gpt-3.5-turbo-0125 | 10 | 5 | 0.7 | 2.374 ± 0.538 | 0.298 ± 0.181 | 76.659 ± 44.318 |
| | gpt-3.5-turbo-0125 | 25 | 5 | 0.7 | 2.701 ± 0.539 | 0.156 ± 0.237 | 127.356 ± 58.155 |
| | gpt-3.5-turbo-0125 | 50 | 5 | 0.7 | 1.649 ± 0.072 | 0.412 ± 0.068 | 59.875 ± 59.008 |
| | gpt-3.5-turbo-0125 | 100 | 5 | 0.7 | 1.515 ± 0.264 | 0.484 ± 0.129 | 246.846 ± 384.317 |
| | gpt-3.5-turbo-0125 | 250 | 5 | 0.7 | 1.147 ± 0.090 | 0.656 ± 0.097 | 60.265 ± 60.187 |
| | gpt-3.5-turbo-0125 | 500 | 5 | 0.7 | 1.006 ± 0.062 | 0.696 ± 0.082 | 44.153 ± 38.803 |
| | gpt-3.5-turbo-0125 | 700 | 5 | 0.7 | 0.914 ± 0.034 | 0.727 ± 0.051 | 18.369 ± 8.975 |
| | knn | 700 | 5 | 0.7 | 1.509 ± 0.216 | 0.581 ± 0.110 | - |
| | krr | 700 | 5 | 0.7 | 0.923 ± 0.108 | 0.793 ± 0.045 | - |
| | gpr | 700 | 5 | 0.7 | 1.293 ± 0.183 | 0.571 ± 0.120 | **3.233 ± 0.006** |
| | gpt-4-0125-preview | 700 | 5 | 0.7 | <u>0.613 ± 0.023</u> | <u>0.907 ± 0.014</u> | 422.664 ± 305.325 |
| | gpt-4o | 700 | 5 | 0.7 | **0.471 ± 0.030** | **0.954 ± 0.004** | <u>17.459 ± 7.878</u> |
| | gpt-4o-mini | 700 | 5 | 0.7 | 0.678 ± 0.028 | 0.882 ± 0.014 | 187.039 ± 243.752 |
| OCM | gpt-3.5-turbo-0125 | 1000 | 1 | 0.05 | 2.836 ± 0.203 | 0.447 ± 0.035 | 13046.086 ± 16116.083 |
| | gpt-3.5-turbo-0125 | 1000 | 2 | 0.05 | 2.362 ± 0.211 | 0.558 ± 0.085 | 6046.114 ± 3163.402 |
| | gpt-3.5-turbo-0125 | 1000 | 5 | 0.05 | 2.544 ± 0.145 | 0.455 ± 0.047 | 15564.800 ± 7553.086 |
| | gpt-3.5-turbo-0125 | 1000 | 10 | 0.05 | 2.545 ± 0.102 | 0.470 ± 0.039 | 14048.686 ± 5874.445 |
| | gpt-3.5-turbo-0125 | 1000 | 5 | 0.01 | 2.274 ± 0.088 | 0.507 ± 0.022 | 1328.537 ± 1502.254 |
| | gpt-3.5-turbo-0125 | 1000 | 5 | 0.1 | 2.253 ± 0.102 | 0.500 ± 0.051 | 13358.177 ± 4370.846 |
| | gpt-3.5-turbo-0125 | 1000 | 5 | 0.5 | 2.395 ± 0.134 | 0.511 ± 0.043 | 1210.133 ± 824.772 |
| | gpt-3.5-turbo-0125 | 1000 | 5 | 1.0 | 2.257 ± 0.052 | 0.551 ± 0.063 | 167.617 ± 214.227 |
| | gpt-3.5-turbo-0125 | 1000 | 5 | 1.5 | 2.795 ± 0.328 | 0.285 ± 0.151 | 62.516 ± 71.184 |
| | gpt-3.5-turbo-0125 | 1 | 5 | 0.7 | 3.868 ± 1.298 | 0.160 ± 0.077 | 51897.096 ± 103774.059 |
| | gpt-3.5-turbo-0125 | 5 | 5 | 0.7 | 2.909 ± 0.279 | 0.255 ± 0.085 | 1094.288 ± 797.185 |
| | gpt-3.5-turbo-0125 | 10 | 5 | 0.7 | 3.998 ± 0.734 | 0.264 ± 0.067 | 1542.355 ± 1845.430 |
| | gpt-3.5-turbo-0125 | 25 | 5 | 0.7 | 2.989 ± 0.156 | 0.207 ± 0.088 | 381.992 ± 647.211 |
| | gpt-3.5-turbo-0125 | 50 | 5 | 0.7 | 2.983 ± 0.127 | 0.286 ± 0.031 | 214.435 ± 159.729 |
| | gpt-3.5-turbo-0125 | 100 | 5 | 0.7 | 3.093 ± 0.064 | 0.287 ± 0.031 | 305.642 ± 229.304 |
| | gpt-3.5-turbo-0125 | 250 | 5 | 0.7 | 2.931 ± 0.296 | 0.374 ± 0.074 | 117.576 ± 45.203 |
| | gpt-3.5-turbo-0125 | 500 | 5 | 0.7 | 2.656 ± 0.172 | 0.411 ± 0.062 | 143.912 ± 61.103 |
| | gpt-3.5-turbo-0125 | 1000 | 5 | 0.7 | 2.219 ± 0.137 | 0.555 ± 0.048 | 139.080 ± 30.690 |
| | knn | 1000 | 5 | 0.7 | 2.171 ± 0.194 | 0.586 ± 0.036 | - |
| | krr | 1000 | 5 | 0.7 | <u>1.934 ± 0.081</u> | **0.723 ± 0.029** | - |
| | gpr | 1000 | 5 | 0.7 | 2.312 ± 0.122 | 0.506 ± 0.058 | **3.265 ± 0.005** |
| | gpt-4-0125-preview | 1000 | 5 | 0.7 | 2.053 ± 0.119 | 0.631 ± 0.021 | 1931.768 ± 758.246 |
| | gpt-4o | 1000 | 5 | 0.7 | **1.863 ± 0.151** | 0.649 ± 0.060 | 26.588 ± 19.877 |
| | gpt-4o-mini | 1000 | 5 | 0.7 | 2.102 ± 0.096 | 0.552 ± 0.039 | 815.057 ± 331.204 |
| | gemini-2.5-flash | 1000 | 5 | 0.7 | 2.050 ± 0.125 | <u>0.624 ± 0.040</u> | <u>12.492 ± 1.693</u> |

### S4.1 Solubility

**Solubility** In this study, we considered three datasets. First, to evaluate BO-ICL, we applied it to the ESOL[5] dataset, using IUPAC names as representations for molecules and measured LogS value labels. This solubility dataset is a benchmark largely used to evaluate models and was employed to allow broad comparison with the literature.

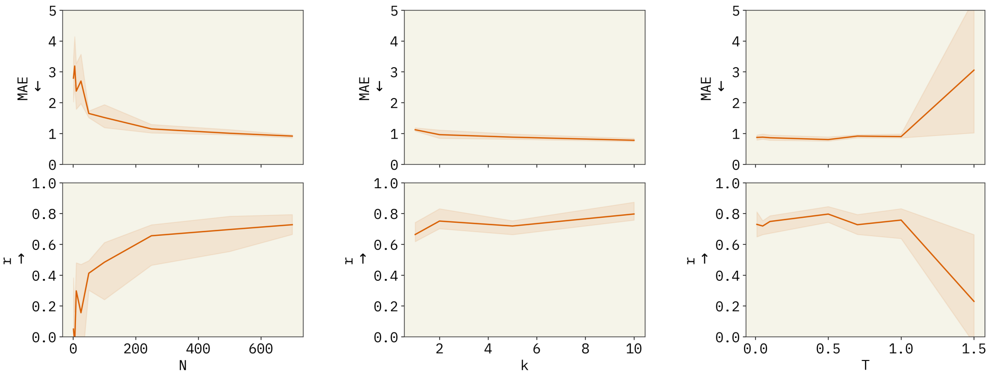


Figure S12: Performance metrics for hyperparameter tuning on the solubility dataset. The model `gpt-3.5-turbo-0.125` shows consistent improvement as the number of samples in the model's memory (N) increases. However, its performance is relatively insensitive to the number of in-context examples (k) and the temperature (T). Only at high temperature values does performance drop significantly, likely due to increased hallucinations.

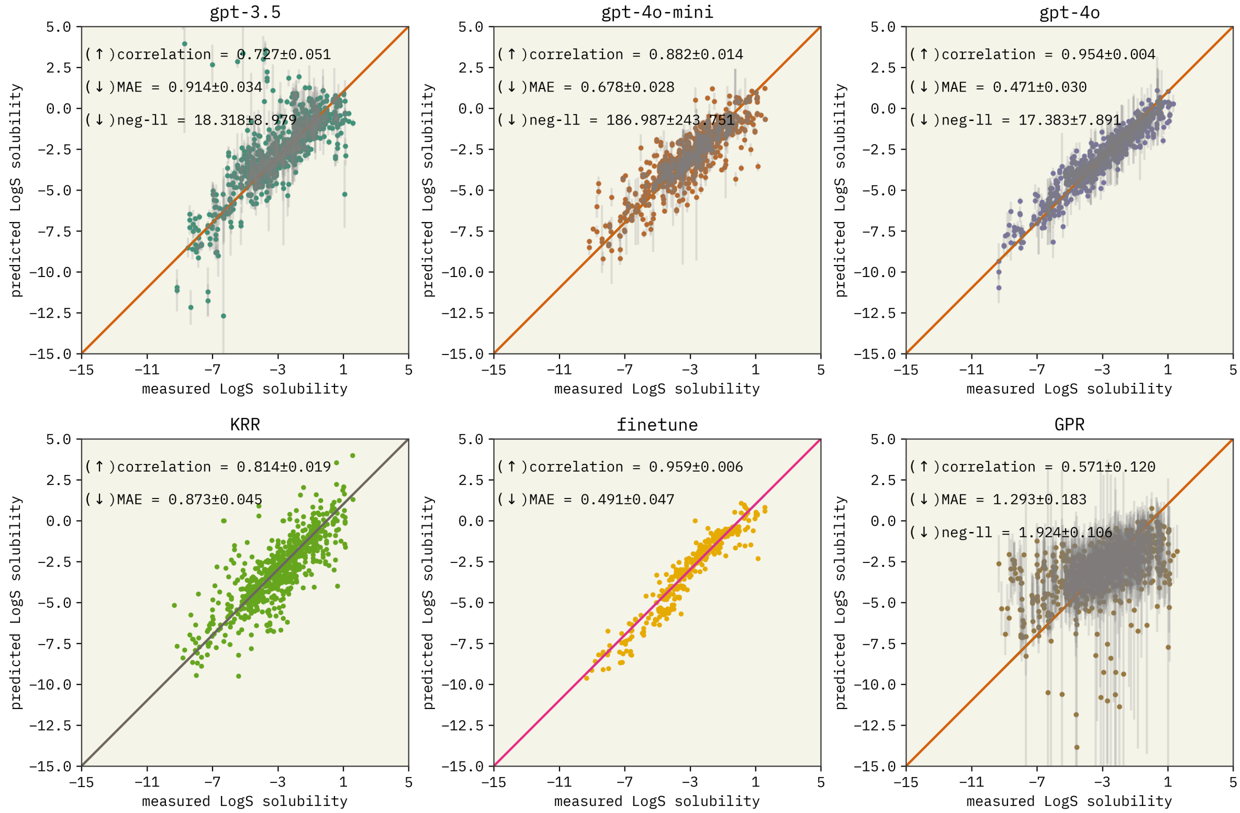


Figure S13: Parity plots for the regression on the solubility dataset task across different models. Each model was evaluated over five independent replicates, with each plot aggregating all predicted vs. true values. Reported metrics reflect the mean and standard deviation across replicates.

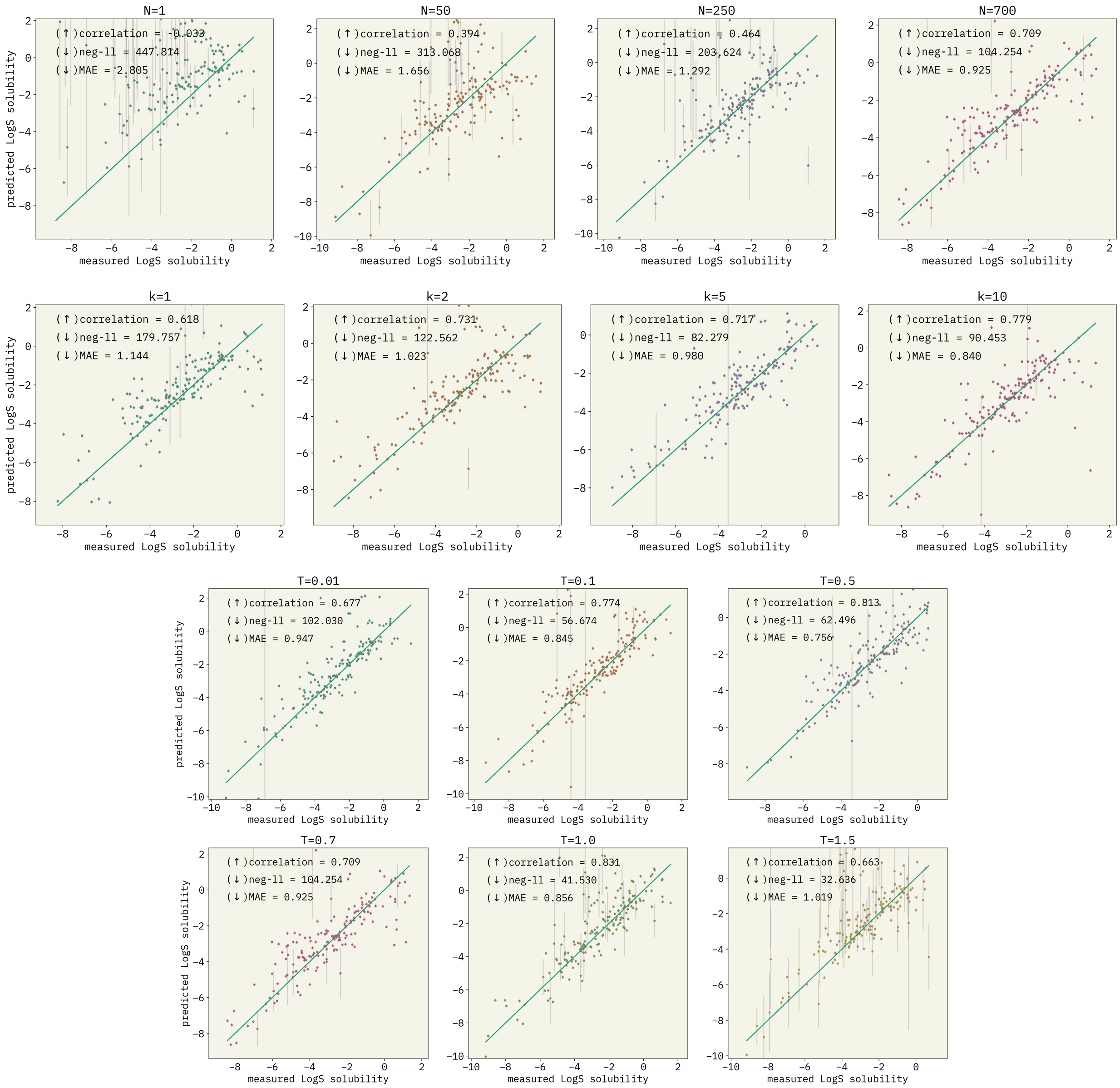


Figure S14: Illustrative parity plots from the hyperparameter tuning experiment on the solubility. Each inset title indicates the hyperparameter being varied. Unless specified otherwise in the title, the default configuration used is N = 700, k = 5, and T = 0.7.

## S4.2 Regression - OCM

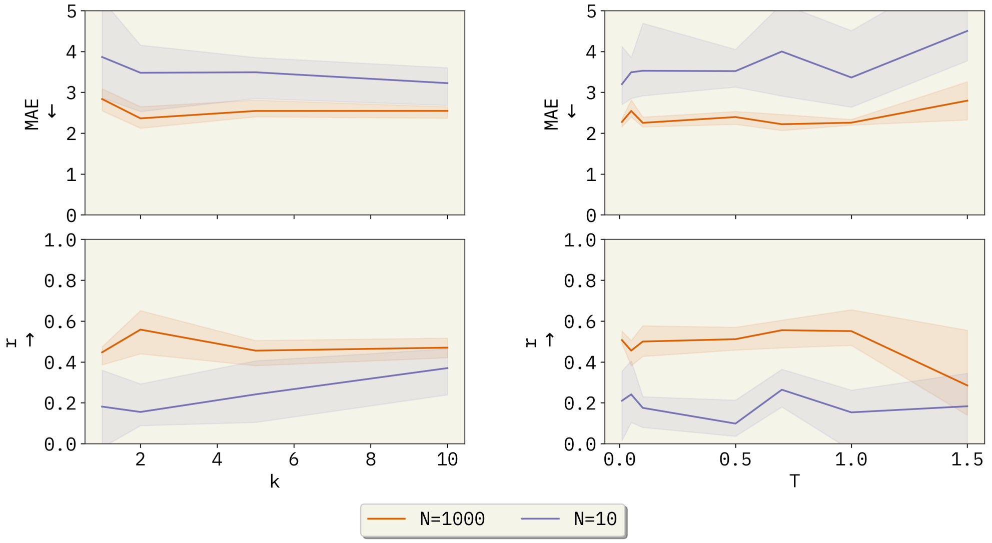


Figure S15: To assess the impact of hyperparameters in low-data scenarios, we evaluated performance under two conditions: N = 1000 and N = 10. In the low-data regime (N = 10), the number of in-context examples (k) plays a more critical role, with performance steadily improving as k increases. In contrast, the effect of temperature (T) remains consistent with previous findings on the solubility dataset: model performance is largely stable across T values, except at high temperatures (T = 1.5), where a notable drop occurs due to increased hallucinations.

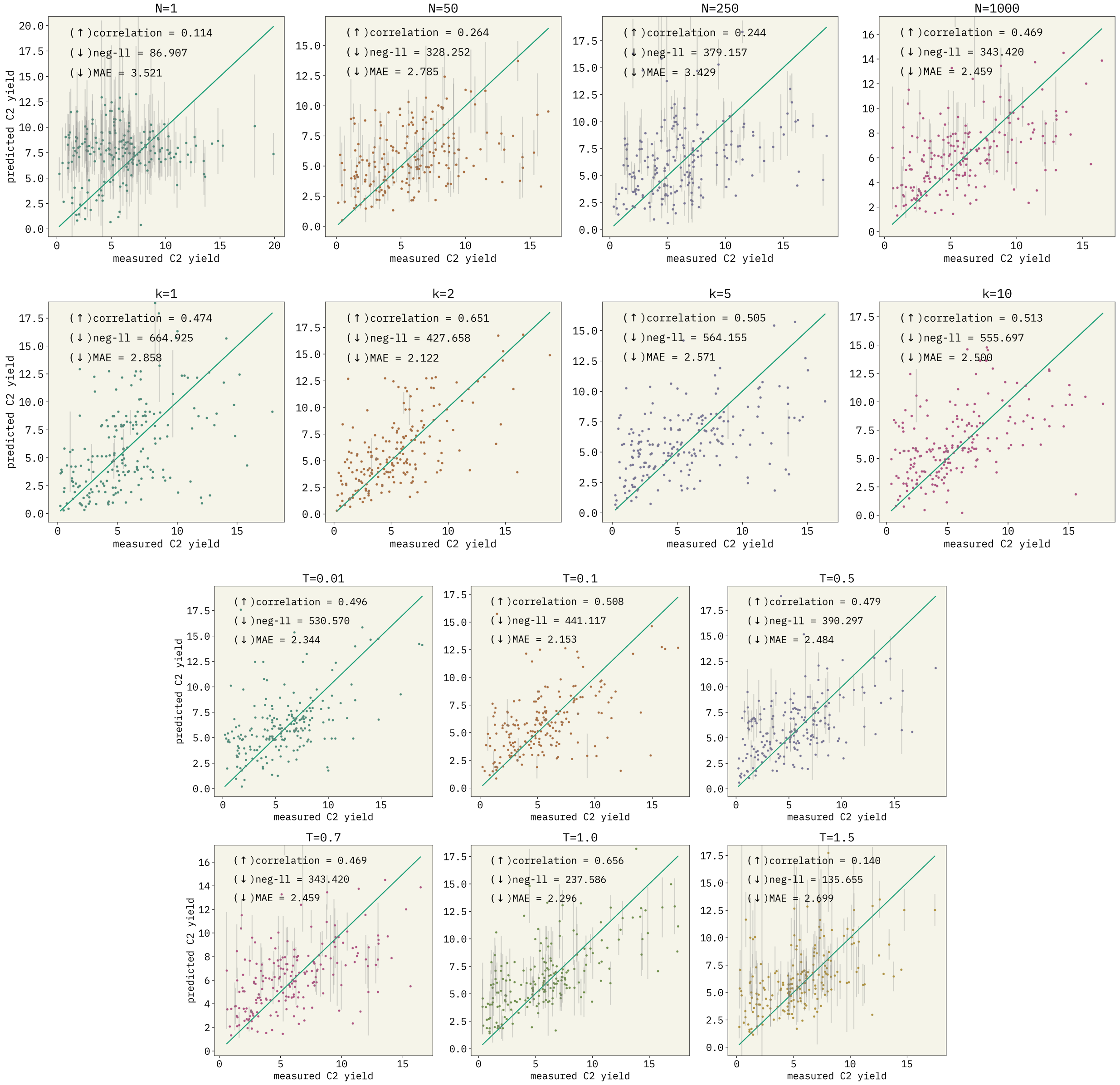


Figure S16: Illustrative parity plots from the hyperparameter tuning experiment on the OCM dataset. Each inset title indicates the hyperparameter being varied. Unless specified otherwise in the title, the default configuration used is N = 1000, k = 5, and T = 0.7.

## S5 Prompts and system messages

The following system message led to better results and was used in every Bayesian optimization result that involved a chat model shown in this study:

```
You are an expert in heterogeneous catalysis, with deep knowledge of the
electronic properties of elements, especially how transition metals interact with
various supports to synergistically catalyze reactions under different conditions.
 Your expertise extends to designing experimental procedures aimed at achieving
desired material properties. Your capabilities are built on the most current and
comprehensive information available, up to April 2023. When responding to
inquiries, please focus on providing specific experimental procedures, without
including explanations. Your approach should reflect a balanced integration of
user-provided information and your base knowledge to identify the most impactful
experimental strategies for their needs. Our goal is to deliver clear and direct
procedural guidance, ENSURING that we match the user's example formats when
responding, to identify the most effective experimental procedure for their needs.

These are the possible parameters that can be input:
catalyst, name, precursor, support, M1b, M2b, M3b
Mn-Na2WO4/BN, Mn(NO3)2.6H2O, Na2WO4, BN, Mn(40), Na(40), W(20)
Mn-Na2WO4/MgO  Mn(NO3)2.6H2O, Na2WO4            MgO
Mn (40)   Na (40)   W (20)
Mn-Na2WO4/Al2O3 Mn(NO3)2.6H2O, Na2WO4            Al2O3
                              Mn (40)   Na (40)   W (20)
Mn-Na2WO4/SiC   Mn(NO3)2.6H2O, Na2WO4            SiC
Mn (40)   Na (40)   W (20)
Mn-Na2WO4/SiCnf Mn(NO3)2.6H2O, Na2WO4            SiCnf
Mn (40)   Na (40)   W (20)
Mn-Na2WO4/BEA    Mn(NO3)2.6H2O, Na2WO4           BEA
Mn (40)   Na (40)   W (20)
Mn-Na2WO4/ZSM-5  Mn(NO3)2.6H2O, Na2WO4           ZSM-5
Mn (40)   Na (40)   W (20)
Mn-Na2WO4/TiO2   Mn(NO3)2.6H2O, Na2WO4           TiO2
Mn (40)   Na (40)   W (20)
Mn-Na2WO4/ZrO2   Mn(NO3)2.6H2O, Na2WO4           ZrO2
Mn (40)   Na (40)   W (20)
Mn-Na2WO4/Nb2O5   Mn(NO3)2.6H2O, Na2WO4          Nb2O5
Mn (40)   Na (40)   W (20)
Mn-Na2WO4/CeO2    Mn(NO3)2.6H2O, Na2WO4          CeO2
Mn (40)   Na (40)   W (20)
Mn-Li2WO4/SiO2    Mn(NO3)2.6H2O, Li2WO4          SiO2
Mn (40)   Li (40)   W (20)
Mn-MgWO4/SiO2     Mn(NO3)2.6H2O, MgWO4           SiO2
Mn (50)   Mg (25)   W (25)
Mn-K2WO4/SiO2      Mn(NO3)2.6H2O, K2WO4          SiO2
Mn (40)   K (40)    W (20)
Mn-CaWO4/SiO2      Mn(NO3)2.6H2O, CaWO4          SiO2
Mn (50)   Ca (25)   W (25)
Mn-SrWO4/SiO2      Mn(NO3)2.6H2O, SrWO4          SiO2
Mn (50)   Sr (25)   W (25)
Mn-BaWO4/SiO2      Mn(NO3)2.6H2O, BaWO4          SiO2
Mn (50)   Ba (25)   W (25)
Mn-Li2MoO4/SiO2    Mn(NO3)2.6H2O, Li2MoO4        SiO2
Mn (40)   Li (40)   Mo (20)
Mn-Na2MoO4/SiO2    Mn(NO3)2.6H2O, Na2MoO4        SiO2
Mn (40)   Na (40)   Mo (20)
Mn-K2MoO4/SiO2     Mn(NO3)2.6H2O, K2MoO4         SiO2
Mn (40)   K (40)    Mo (20)
Mn-FeMoO4/SiO2     Mn(NO3)2.6H2O, FeMoO4         SiO2
Mn (50)   Fe (25)   Mo (25)
Mn-ZnMoO4/SiO2     Mn(NO3)2.6H2O, ZnMoO4         SiO2
Mn (50)   Zn (25)   Mo (25)
```

```
Ti-Na2WO4/SiO2      Ti(OiPr)4, Na2WO4           SiO2
                  Ti (40)   Na (40)   W (20)
V-Na2WO4/SiO2       VOSO4.xH2O (x = 3-5), Na2WO4 SiO2
                  V (40)    Na (40)   W (20)
Fe-Na2WO4/SiO2      Fe(NO3)3.9H2O, Na2WO4       SiO2
                  Fe (40)   Na (40)   W (20)
Co-Na2WO4/SiO2      Co(NO3)2.6H2O, Na2WO4       SiO2
                  Co (40)   Na (40)   W (20)
Ni-Na2WO4/SiO2      Ni(NO3)2.6H2O, Na2WO4       SiO2
                  Ni (40)   Na (40)   W (20)
Cu-Na2WO4/SiO2      Cu(NO3)2.5H2O, Na2WO4       SiO2
                  Cu (40)   Na (40)   W (20)
Zn-Na2WO4/SiO2      Zn(NO3)2.6H2O, Na2WO4       SiO2
                  Zn (40)   Na (40)   W (20)
Y-Na2WO4/SiO2       Y(NO3)3.6H2O, Na2WO4        SiO2
                  Y (40)    Na (40)   W (20)
Zr-Na2WO4/SiO2      ZrO(NO3)2.2H2O, Na2WO4     SiO2
Zr (40)   Na (40)   W (20)
Mo-Na2WO4/SiO2      (NH4)2MoO4, Na2WO4          SiO2
                  Mo (40)   Na (40)   W (20)
Pd-Na2WO4/SiO2      Pd(OAc)2, Na2WO4            SiO2
                  Pd (40)   Na (40)   W (20)
La-Na2WO4/SiO2      La(NO3)3, Na2WO4            SiO2
                  La (40)   Na (40)   W (20)
Ce-Na2WO4/SiO2      Ce(NO3)3.6H2O, Na2WO4       SiO2
                  Ce (40)   Na (40)   W (20)
Nd-Na2WO4/SiO2      Nd(NO3)3.6H2O, Na2WO4       SiO2
                  Nd (40)   Na (40)   W (20)
Eu-Na2WO4/SiO2      Eu(NO3)3.5H2O, Na2WO4       SiO2
                  Eu (40)   Na (40)   W (20)
Tb-Na2WO4/SiO2      Tb(NO3)3.5H2O, Na2WO4       SiO2
                  Tb (40)   Na (40)   W (20)
Hf-Na2WO4/SiO2      Hf(OEt)4, Na2WO4            SiO2
                  Hf (40)   Na (40)   W (20)
blank             -                            -
                     -        -        -        -
BN                -                            BN
                    -        -        -        -
MgO               -                            MgO
                   -        -        -        -
Al2O3             -                            Al2O3
                 -        -        -        -
SiO2              -                            SiO2
                  -        -        -        -
SiC               -                            SiC
                   -        -        -        -
SiCnf             -                            SiCnf
                 -        -        -        -
BEA               -                            BEA
                   -        -        -        -
ZSM-5             -                            ZSM-5
                 -        -        -        -
TiO2              -                            TiO2
                  -        -        -        -
ZrO2              -                            ZrO2
                  -        -        -        -
Nb2O5             -                            Nb2O5
                 -        -        -        -
CeO2              -                            CeO2
                  -        -        -        -
Na2WO4/SiO2       Na2WO4                       SiO2
                  -         Na (67)  W (33)   -
Mn-WOx/SiO2       Mn(NO3)2.6H2O, (NH4)10H2(W2O7)6 SiO2
                 Mn (67)   -        W (33)   -
```

```
Mn-MoOx/SiO2      Mn(NO3)2.6H2O, (NH4)2MoO4      SiO2
                            Mn (67)   -          Mo (33)   -
Mn-Na/SiO2        Mn(NO3)2.6H2O, NaNO3           SiO2
                            Mn (50)   Na (50)    -
WOx/SiO2          (NH4)10H2(W2O7)6                SiO2
                            -         -          -         W (100)
Na/SiO2           NaNO3                            SiO2
                            -         Na (100) -         -

Your experimental procedures can only use those parameters. This is more
information to help control how you output these procedures: The metal loadings to
 a unit gram of support were fixed at 0.371 mmol for Metal 1, 0.370 or 0.185 mmol
for Metal 2 (depending on the valence), and 0.185 mmol for Metal 3. The values in
parentheses need to correspond to relative atomic percentages of M1-M3: to a unit
gram of the support, only choose from, 0.371 mmol M1, 0.370 or 0.185 mmol M2, and
0.185 mmol M3 or 0.0. Use the exact format of the given examples when responding,
given a property like C2 yield.
```

## S6 Cost Analysis

To address monetary considerations associated with running BO-ICL, we present the cost and process parameters obtained directly from OpenAI for a complete run as presented in aforementioned results (Figure 4). This analysis considers the most expensive model used in this study (`gpt-4-0125-preview`) as well as the embedding model (`Ada-small`) (see Table S6). The sub-sampling strategy illustrated in points A4–A8 of the flowchart (Figure 8) is central to achieving the observed low cost. The prediction and update steps incur costs only for output tokens, billed at $10.00 per 1M output tokens, and involve 16 samples per iteration. Additionally, a single inverse design generation step contributes a cost corresponding to the number of tokens required to represent a single experimental procedure. Aside from this, there is a nominal one-time cost for embedding the initial pool ($\$0.1/1M$ token) after which the embedding representations are cached and reused throughout the design loop. This further supports using BO-ICL even with SOTA models at low cost deploy, contrary to reported mention [16].

Table S6: BO-ICL usage, cost evaluation for the Alloy, In-House RWGS, and OCM dataset - catalyst material costs omitted

| Dataset | Samples | Replicates | Iterations | Inverse Design Model | Prediction Model | Total Cost |
|---|---|---|---|---|---|---|
| AII | 9,000 | 5 | 30 | `gpt-4o-2024-08-06` | `gpt-4o-2024-08-06` | $ 10.52 |
| OCM | 12,708 | 5 | 30 | `gpt-4o-2024-08-06` | `gpt-4o-2024-08-06` | $ 12.22 |
| RWGS | 3,720 | 2 | 6 | `gpt-4o-2024-08-06` | `gpt-4o-2024-08-06` | $ 0.98 |

## S7 Effect of variation in language expression

We have explicitly evaluated how superficial changes and chemically meaningful edits affect the embedding representations used in BO-ICL. We hypothesize that changes in embedding feature vectors correlate strongly with inference variance; therefore, measuring the cosine similarity between a reference procedure and a counterfactual should reveal the relative importance of specific parameters, which may guide future procedure structure selection. Using reference catalytic procedures from the trimetallic dataset (Reference example S1), we generated controlled single-parameter variants spanning: (**i**) surface-form perturbations (punctuation, spacing, formatting), (**ii**) unit-equivalent temperature rewrites (e.g., °C vs K), (**iii**) additional temperature annotations (e.g., appended parenthetical conversions), and (**iv**) chemically meaningful changes that affect catalytic performance (e.g., pre-treatment gas identity)

Changes were quantified by the embedding distance between reference and variant text using cosine similarity. We find that even minor textual edits produce measurable embedding shifts above numerical noise. Chemically meaningful changes (e.g., pre-treatment gas identity) consistently produce larger representation changes than purely notational or formatting variations, indicating sensitivity to catalytically relevant information. However, adding new numeric or unit content (e.g., appending temperature conversions) can induce embedding changes comparable to, or larger than, chemistry edits. Thus, while the embeddings capture chemically relevant distinctions, they also remain sensitive to surface form and numeric content, consistent with their general language-pretraining objective.

Inverse-design outputs are generated in the same procedural natural language format used in the training dataset, guided by the context and system message (Section S5). Because BO-ICL operates directly on full-text representations rather than structured features, exact syntactic alignment is not required. However, sensitivity to numeric and formatting content means that inconsistent expression (e.g., duplicated units, redundant annotations, or unusual phrasing) can influence embedding geometry and therefore candidate ranking. To mitigate this, prompts are constructed to encourage consistent procedural formatting, and candidate texts are evaluated in the same representational space as the training data. This helps ensure that retrieval and scoring operate on structurally comparable descriptions, even when phrasing varies.

Across the single-parameter variants we tested, superficial edits (punctuation/spacing) and alternate temperature spellings (e.g., `300 °C` vs. `300 Celsius`) produce small, but measurable changes above noise. Chemically meaningful substitutions, such as changing the pretreatment gas ($CO \rightarrow H_2$ or Ar), consistently induce shifts that are typically larger than temperature formatting. However, embedding distance is driven most strongly by added numeric content: appending an equivalent temperature conversion (e.g., "`300 °C (573 K)`") produces the largest representation shift. Because BO-ICL performs retrieval and candidate ranking directly in the embedding space, these expression-driven shifts can change which procedures are retrieved and how candidates are ordered, even when the intended physical conditions are equivalent. To keep inverse-designed outputs comparable to the training data, we use the same format and parameter

ordering as the corpus, and prompts are designed with consistent formatting. When candidates include inconsistent or redundant expressions (e.g., duplicated units, repeated conversions, or atypical phrasing), their embeddings can bias retrieval and scoring, motivating controlled textual templates for both interpretation and inverse design.”

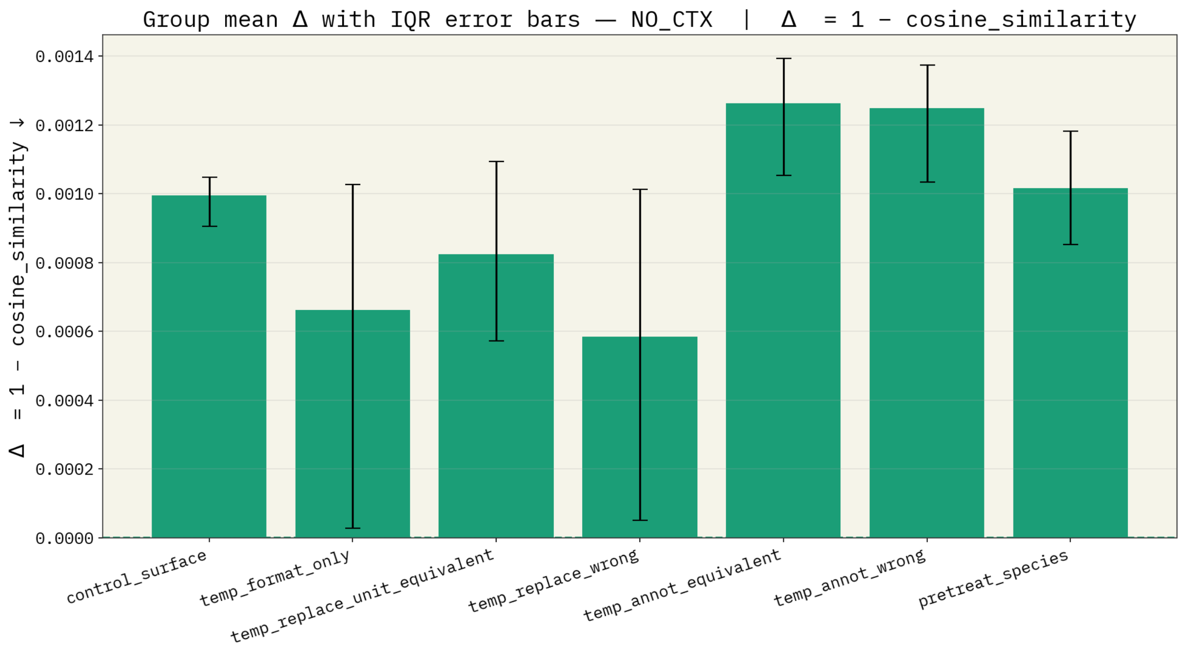


Figure S17: Mean embedding distance by perturbation class under the no-context condition (`text-embedding-ada-002`). Bars show the mean semantic distance $\Delta = 1 - \cos(\mathbf{e}_{\text{ref}}, \mathbf{e}_{\text{var}})$ between the reference catalytic procedure and single-parameter variants grouped by edit type. Error bars indicate the interquartile range (25th–75th percentile) across variants within each group. Larger $\Delta$ indicates greater deviation from the reference embedding. Temperature annotations and unit-equivalent substitutions produce the largest representation shifts, while surface-form edits and temperature-format-only changes produce smaller but measurable effects. Chemically meaningful pretreatment gas substitutions yield intermediate-to-large shifts, indicating sensitivity to catalytically relevant parameters alongside numeric and unit content.

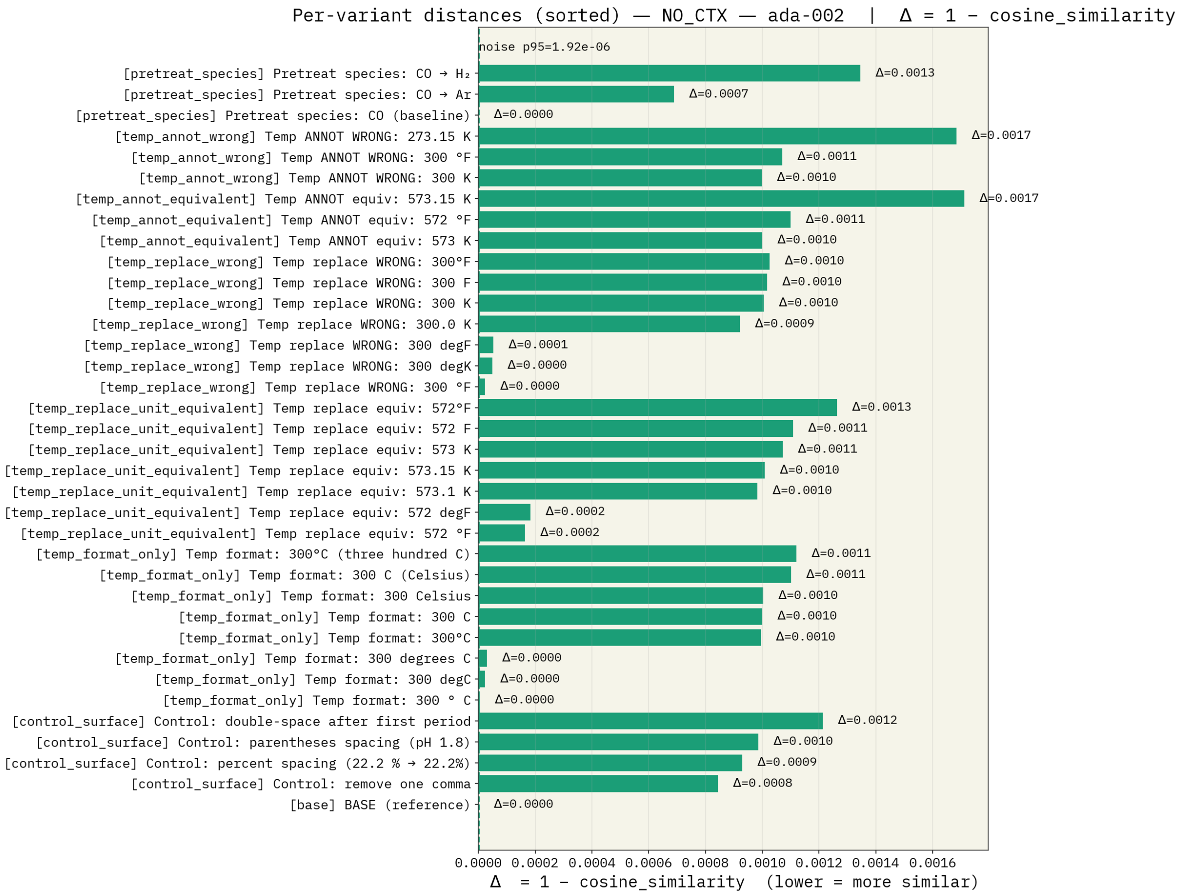


Figure S18: Sorted embedding distances for controlled textual perturbations of a catalytic procedure (no contextual augmentation; `text-embedding-ada-002`). Bars show semantic distance $\Delta = 1 - \cos(\mathbf{e}_{\text{ref}}, \mathbf{e}_{\text{var}})$ from the reference description. The dashed line indicates the 95th percentile of duplicate-embedding noise. Surface-level formatting edits cluster near the noise floor, whereas temperature annotations and unit-equivalent substitutions produce substantially larger embedding shifts. Species substitutions in the pretreatment step also induce measurable semantic change. Results demonstrate that embedding sensitivity is dominated by numerically explicit temperature information rather than minor typographic variation.

**Reference example S1: (highlighted spans indicate edited regions).** "A trimetallic catalyst was synthesized by incipient-wetness co-impregnation onto $CeO_2$ , with 5.0 wt % total metal loading and 0.5 wt % potassium promoter. The active metals were Zn, Mo, and W in a molar ratio of 2.1:1.0:1.5. Due to differing pH requirements, sequential impregnation was employed. Initially, acidic-stable precursors were dissolved in $HNO_3$-acidified Milli-Q water (pH 1.8), impregnated onto the support, dried at 90 °C for 4 h, and calcined at 450 °C. Subsequently, a solution containing ammonium molybdate (para) tetrahydrate and ammonium metatungstate hydrate was used in a second impregnation step (~pH 4), followed by drying and final calcination. Prior to testing, the catalyst was reduced in CO at 600 °C under 50 psig , at 20 mL/min. Testing was performed at 300 °C using a gas mixture of $CO_2$ (22.2 %), $H_2$ (66.7 %), and Ar (11.1 %) at 45 mL/min and 1 atm. The sample loading was 75 mg, corresponding to a GHSV of 54,000 $h^{-1}$."

*Highlighted spans mark the minimal text regions perturbed to create single-parameter variants, including surface-form edits, temperature expression/annotation variants centered on 300 °C, and chemically meaningful pretreatment gas substitutions CO → $H_2$ or Ar.*

Table S7: Summary of single-parameter text variants used to probe embedding sensitivity.

| Variant group | What is changed | Example edits | Intended interpretation |
|---|---|---|---|
| Base | No change (reference vs. itself) | `BASE (reference)` | Sanity check: self-comparison should yield $\Delta \approx 0$ (up to numerical noise). |
| Surface-form controls | Punctuation/whitespace/ typography only (no chemical meaning change) | Double-space after first period; remove one comma; `22.2%` → `22.2%`; `(pH 1.8)` → `( pH 1.8 )` | Measures sensitivity to *surface form* rather than chemistry. |
| Temperature format-only | Temperature formatting changes; value stays `300 °C` | `300°C`, `300 C`, `300 degC`, `300 degrees C`, `300 Celsius`, `300 ° C` | Tests sensitivity to notation/lexical form for the same physical condition. |
| Temperature unit-equivalent replacement | Replace `300 °C` with a correct equivalent in another unit (numerals change) | `573.15 K`, `573 K`, `573.1 K`, `572 °F`, `572 degF` | Tests whether physically equivalent conditions remain similar despite different numerals/units. |
| Temperature wrong replacement | Replace `300 °C` with a non-equivalent or incorrect unit/value (often keeping "300") | `300 K`, `300.0 K`, `300 °F`, `300 degF`, `300 degK` | Tests whether physically incorrect changes produce larger $\Delta$ than equivalent replacements. |
| Temperature annotation (equivalent) | Keep `300 °C` and add an equivalent parenthetical | `300 °C (573.15 K)`; `300 °C (572 °F)` | Controlled test: isolates effect of added context while preserving the original temperature string. |
| Temperature annotation (wrong) | Keep `300 °C` and add a contradictory parenthetical | `300 °C (300 K)`; `300 °C (300 °F)`; `300 °C (273.15 K)` | Tests whether models penalize contradictory physical information when the correct value remains present. |
| Pretreatment gas species | Change only the reduction gas identity in pretreatment | Baseline: `reduced in CO`; swaps: `CO` → $H_2$, `CO` → `Ar` | Chemically consequential change: pretreatment gas can strongly affect catalyst state/performance. |
| Context wrapping (experimental condition) | Add additional procedures as surrounding context while still modifying only the original reference text | `NO_CTX`, `CTX_ref1`, `CTX_ref2`, `CTX_ref1_ref2`, `CTX_ref1_ref2__TAIL_ORIG` | Tests whether chemical context shifts relative sensitivity (e.g., chemistry changes vs. surface-form changes). |

## References


[1] Melis S Duyar, Martha A Arellano Treviño, and Robert J Farrauto. Dual function materials for CO2 capture and conversion using renewable H2. *Appl. Catal. B*, 168-169:370–376, June 2015.

[2] Daisy Dunne and Robert McSweeney. Nine key takeaways about the 'state of CO2 removal' in 2024. `https://www.carbonbrief.org/nine-key-takeaways-about-the-state-of-co2-removal-in-2024/`, June 2024. Accessed: 2024-7-15.

[3] Thanh Nhat Nguyen, Thuy Tran Phuong Nhat, Ken Takimoto, Ashutosh Thakur, Shun Nishimura, Junya Ohyama, Itsuki Miyazato, Lauren Takahashi, Jun Fujima, Keisuke Takahashi, and Toshiaki Taniike. High-Throughput experimentation and catalyst informatics for oxidative coupling of methane. *ACS Catal.*, 10(2):921–932, January 2020. doi:10.1021/acscatal.9b04293.

[4] Tuan Dinh, Yuchen Zeng, Ruisu Zhang, Ziqian Lin, Michael Gira, Shashank Rajput, Jy-Yong Sohn, Dimitris Papailiopoulos, and Kangwook Lee. LIFT: Language-Interfaced Fine-Tuning for Non-Language machine learning tasks. June 2022.

[5] John S Delaney. ESOL: estimating aqueous solubility directly from molecular structure. *J. Chem. Inf. Comput. Sci.*, 44(3):1000–1005, 2004. ISSN 0095-2338. doi:10.1021/ci034243x.

[6] David Weininger. SMILES, a chemical language and information system. 1. introduction to methodology and encoding rules. *J. Chem. Inf. Comput. Sci.*, 28(1):31–36, February 1988. ISSN 0095-2338,1520-5142. doi:10.1021/ci00057a005. URL `https://pubs.acs.org/doi/abs/10.1021/ci00062a008`.

[7] Sunghwan Kim, Paul A Thiessen, Tiejun Cheng, Bo Yu, and Evan E Bolton. An update on PUG-REST: RESTful interface for programmatic access to PubChem. *Nucleic Acids Res.*, 46(W1):W563–W570, July 2018. ISSN 0305-1048,1362-4962. doi:10.1093/nar/gky294. URL `http://dx.doi.org/10.1093/nar/gky294`.

[8] Eli Gerber, Steven B Torrisi, Sara Shabani, Eric Seewald, Jordan Pack, Jennifer E Hoffman, Cory R Dean, Abhay N Pasupathy, and Eun-Ah Kim. High-throughput ab initio design of atomic interfaces using InterMatch. *Nat. Commun.*, 14(1):7921, December 2023.

[9] Weiting Yu, Marc D Porosoff, and Jingguang G Chen. Review of pt-based bimetallic catalysis: From model surfaces to supported catalysts. *Chem. Rev.*, 112(11):5780–5817, November 2012.

[10] James W M Crawley, Isla E Gow, Naomi Lawes, Igor Kowalec, Lara Kabalan, C Richard A Catlow, Andrew J Logsdail, Stuart H Taylor, Nicholas F Dummer, and Graham J Hutchings. Heterogeneous trimetallic nanoparticles as catalysts. *Chem. Rev.*, 122(6):6795–6849, March 2022.

[11] C Saunders, A Gammerman, and V Vovk. Ridge regression learning algorithm in dual variables. *ICML*, pages 515–521, July 1998. URL `https://eprints.soton.ac.uk/258942/1/Dualrr_ICML98.pdf`.

[12] Kevin Vu, John Snyder, Li Li, Matthias Rupp, Brandon F Chen, Tarek Khelif, Klaus-Robert Müller, and Kieron Burke. Understanding kernel ridge regression: Common behaviors from simple functions to density functionals. *arXiv [physics.comp-ph]*, January 2015. URL `http://arxiv.org/abs/1501.03854`.

[13] Carl Edward Rasmussen and Christopher K I Williams. *Gaussian Processes for Machine Learning*. MIT Press, November 2005. ISBN 9780262182539.

[14] Peter I Frazier. A tutorial on bayesian optimization. July 2018.

[15] Maximilian Balandat, Brian Karrer, Daniel R. Jiang, Samuel Daulton, Benjamin Letham, Andrew Gordon Wilson, and Eytan Bakshy. BoTorch: A Framework for Efficient Monte-Carlo Bayesian Optimization. In *Advances in Neural Information Processing Systems 33*, 2020. URL `http://arxiv.org/abs/1910.06403`.

[16] Agustinus Kristiadi, Felix Strieth-Kalthoff, Marta Skreta, Pascal Poupart, Alán Aspuru-Guzik, and Geoff Pleiss. A sober look at LLMs for material discovery: Are they actually good for bayesian optimization over molecules? *arXiv [cs.LG]*, February 2024. URL `http://arxiv.org/abs/2402.05015`.
