# Supplementary material for: Bayesian Optimization of Catalysis with In-Context Learning
Source: ACS Cent Sci. 2026 Apr 14;12(5):599–615. doi: 10.1021/acscentsci.5c02418 (PMC13220214; doi:10.1021/acscentsci.5c02418)
Supplement: Supplementary file 2 [file oc5c02418_si_002.pdf]

oc-2025-02418s.R1

Name: Peer Review Information for "Bayesian optimization of catalysis with in-context learning"

First Round of Reviewer Comments

Reviewer: 1

Comments to the Author

This paper introduces a novel BO-ICL framework that integrates Bayesian optimization (BO) with in-context learning (ICL) of large language models (LLMs), tailored for design optimization in materials science. The efficacy of this proposed method is validated across multiple benchmark datasets and experimental synthesis. The work is technically sound and well aligned with the scope of the ACS Central Science. However, before publication, a major revision is quite needed, and the comments are listed as follows:

1. The authors should clarify the specific advantages of their proposed BO-ICL compared to Gaussian process regression (GPR) using LLM embeddings. Although the abstract mentions that the assumption of smoothness in GPR poses challenges in heterogeneous catalysis, the examples show that BO-ICL performs similarly to GPR. Additionally, while BO-ICL avoids complex hyperparameter tuning of the model itself, it still requires adjustments for parameters in sub-pool construction. The authors should explicitly compare their method to existing BO-GP applications and explain why BO-ICL offers a clear advantage in catalysis optimization.

2. The authors should compare the performance of their proposed method with a direct approach where LLM is used to recommend the next experimental sample, similar to the inverse design used for generating reference points in sub-pool construction. Such a comparison is essential to elucidate the specific role of BO within the BO-ICL.

3. The authors should consider the potential biases introduced by the sub-pool construction, which involves a single round of inverse design and MMR pre-screening based on the result of inverse design to reduce token consumption. Relying on just one

round of inverse design and the MMR method may introduce biases, potentially leading to the exclusion of samples with higher acquisition function scores.

4. The authors should discuss how variations in language expression (e.g., "900℃" vs "900 Celsius" vs "reaction temperature set to 1173K") impact model performance. Additionally, they should clarify whether the text structure generated through inverse design aligns with the structure of data, and explain how any inconsistencies could affect the retrieval or prediction processes.

5. What is the difference between the "completion model" and the "chat model" in Figure 5? According to the text, both appear to be based on GPT-4o, with only the acquisition function differing.

6. The authors should carefully review and revise the manuscript to address basic errors throughout the text, e.g.,

a) The abstract should specify the full form of "ICL" for clarity, as it is used without definition.

b) The abbreviation "KRR" is inconsistently capitalized throughout the manuscript, for example, lines 13, 34, 38, and 54 on page 4

c) What do the shaded areas represent in Figures 4 and 5? And, in Figure 4, what do the darker lines (but not the darkest) within the shaded area represent for UCB and Greedy?

d) The "upper confidence bound" in the legend of Figure 5 should be formatted consistently with Figure 4, using the abbreviation instead of the full form.

e) On page 14, line 19, Algorithm 2 is mentioned, but it does not appear in the main text.

f) On page 14, line 10, the manuscript references the pseudo-code for the BO-ICL implementation (Algorithm 1), but the actual Algorithm 1 shown is the "Bayesian Optimization Policy for Reaction Runs."

g) What is the full form of "MMR" mentioned on page 14 and “RAG” mentioned on page 4.

h) In line 3 of page 14, the "T" in "Total" should be lowercase.

i) There is an inconsistency between the order of subfigures in Figure 7 and the description in the caption. Additionally, the caption mentions the catalyst formulation K@ZnZrMo(8.0:1.0:2.0)/TiO_2, but this is not shown in the figure. Meanwhile, the

K@ZnMoMn(2.1:3.0:1.5)/CeO2 formulation is introduced in the figure but is not discussed in the text.

Reviewer: 2

Comments to the Author

This manuscript introduces BO-ICL, a framework that employs large language models (LLMs) as surrogate models for Bayesian optimization (BO) in catalyst discovery. By leveraging in-context learning (ICL), the authors demonstrate that frozen LLMs can directly map natural language descriptions of experimental procedures to catalytic performance, effectively bypassing traditional feature engineering and explicit model training. This work is innovative and compellingly bridges generative AI with experimental automation. This manuscript is suitable for publication in ACS Central Science after minor revisions. Below are some comments.

1.The authors first use an LLM-based inverse design to generate a ideal procedure (x'), followed by MMR-based sub-sampling from the candidate pool U. The rationale for not using the LLM to directly recommend N ideal procedures should be clarified. Furthermore, the validity of this virtual x' lacks benchmarking. If the generated x' is strongly biased by the model's prior, subsequent MMR retrieval may be confined to a potentially erroneous subspace.

2.The manuscript applies a fixed uncertainty scaling factor (e.g., 5), which is optimized on the OCM dataset, directly to other tasks such as RWGS. The authors should provide a justification for why a scalar parameter tuned on one task exhibits such strong cross-domain transferability.

3.As the BO loop progresses, the labeled dataset L (and thus the memory M) increases in both size and quality. The authors should discuss whether keeping a constant k value remains optimal throughout the process.

4.Sub-sampling is employed across all datasets to manage API latency and cost. If feasible, the authors should provide a benchmark on a smaller dataset comparing sub-sampling against a full global predictive search.

5.In Table S4, a parameter configuration is listed as N = 1 with k = 5. Mathematically, if the total number of observations N is 1, it is impossible to retrieve k = 5 distinct and relevant examples for the prompt context. The authors should clarify the definitions of N and k in this setting or correct any potential labeling errors to ensure the integrity of the supplementary results.

6.The mathematical expression for MMR in Figure 8 appears to contain an error. A thorough check of the manuscript is recommended to ensure consistent and accurate notation throughout.

Author's Response to Peer Review Comments:

Response to Reviewer Comments for:
Bayesian optimization of catalysis with in-context learning

Reviewer 1:

This paper introduces a novel BO-ICL framework that integrates Bayesian optimization (BO) with in-context learning (ICL) of large language models (LLMs), tailored for design optimization in materials science. The efficacy of this proposed method is validated across multiple benchmark datasets and experimental synthesis. The work is technically sound and well aligned with the scope of the ACS Central Science. However, before publication, a major revision is quite needed, and the comments are listed as follows:

Response:

Thank you for your helpful and insightful comments. They have helped clarify the findings of our manuscript and added rigor that will be useful to the readers of *ACS Central Science.*

1. The authors should clarify the specific advantages of their proposed BO-ICL compared to Gaussian process regression (GPR) using LLM embeddings. Although the abstract mentions that the assumption of smoothness in GPR poses challenges in heterogeneous catalysis, the examples show that BO-ICL performs similarly to GPR. Additionally, while BO-ICL avoids complex hyperparameter tuning of the model itself, it still requires adjustments for parameters in sub-pool construction. The authors should explicitly compare their method to existing BO-GP applications and explain why BO-ICL offers a clear advantage in catalysis optimization.

Response:

Thank you for this insightful comment. We agree that the manuscript should more clearly articulate the practical advantages and limitations of BO-ICL relative to

Bayesian optimization with Gaussian process surrogates using LLM embeddings (BO-GP), particularly given that both approaches achieve similar optimization performance in our benchmarks.

In this work, BO-ICL is not intended to universally outperform BO-GP numerically. Rather, it is designed to provide a simpler and more robust optimization workflow when working with language-derived representations, and the discrete, irregular design spaces typical of heterogeneous catalysis.[1] The key differences between the approaches are as follows:

(1) Model construction and training

1. BO-GP requires explicit surrogate specifications, including kernel selection, parameter estimation, and numerical stabilization procedures. These modeling choices can substantially affect optimization behavior.
2. In contrast, BO-ICL uses the pretrained LLM directly as an implicit surrogate and requires no model training or kernel engineering.

(2) Hyperparameter burden

1. BO-GP involves multiple interacting hyperparameters, including kernel parameters, acquisition optimization settings, and dimensionality-reduction parameters when embeddings are high-dimensional. These choices can significantly affect model stability and performance.
2. BO-ICL does introduce parameters related to sub-pool construction; however, these do not involve surrogate model fitting and can be specified using straightforward heuristics that describe the system. In our experience, model predictions are less sensitive to these settings than to GP kernel or dimensionality-reduction.

(3) High-dimensional embedding representations

1. LLM embeddings are typically high-dimensional (1536 dimensions in our work), which can make GP training computationally expensive and numerically ill-conditioned, particularly in small-data regimes. To enable stable BO-GP optimization, we applied dimensionality reduction (Isomap to 32 dimensions), which introduced additional modeling assumptions and hyperparameters (*e.g.,* neighborhood size, target dimension).
2. In contrast, BO-ICL operates directly on the original representation without dimensionality reduction, potentially leading to higher quality predictions, particularly with higher dimension embeddings.

(4) Small-data operation

1. Catalytic optimization campaigns often operate with limited experimental data. GP surrogates can become poorly conditioned or unstable with small sample sizes.
2. BO-ICL is able to predict performance across multiple applications with minimal initialization, because it does not require surrogate fitting.

(5) Suitability for heterogeneous catalysis design spaces

1. Catalytic performance often exhibits abrupt changes arising from discrete compositional or structural differences. Such behavior may violate the smoothness and stationarity assumptions implicit in many commonly used GP kernels.
2. BO-ICL does not impose an explicit smoothness model, and instead relies on semantic structure encoded in the pretrained representations and few-shot in-context learning.

Despite these structural differences between BO-GP and BO-ICL, our results show that BO-ICL achieves performance comparable to BO-GP when both methods use the same LLM-derived representations. We therefore position BO-ICL not as a universally more accurate surrogate, but as a practically convenient, ready-to-use alternative that avoids surrogate training, reduces modeling choices, and remains stable in small data settings encountered in catalytic optimization.

Action:

We have revised the manuscript with the following additions:

1. Comparing BO-ICL and BO-GP in terms of modeling assumptions, hyperparameters, and data requirements.
2. Clarifying that comparable performance is obtained despite BO-ICL having a simpler and more robust modeling pipeline.
3. Emphasizing that the primary advantage of BO-ICL is practical usability in language-based catalysis optimization workflows.

These revisions appear in **Section 2.2 (page 7)**:

"*Across optimization scenarios using adopted datasets, we benchmark BO-ICL against Bayesian optimization with Gaussian process surrogates (BO-GP), implemented as a widely used baseline. To ensure comparability, both approaches operate on identical feature representations derived from LLM embeddings.*

We acknowledge that Gaussian process surrogates are not always naturally suited to language-derived feature spaces or to the discrete, irregular design landscapes characteristic of heterogeneous catalysis. Small changes in catalyst composition, support identity, or promoter loading can produce abrupt performance shifts that violate the smoothness and assumptions implicit in many commonly used GP

kernels. In practice, obtaining accurate BO-GP predictions requires multiple modeling choices and hyperparameter adjustments that are not required with BO-ICL.

We hypothesize that language-based representations in pretrained LLM embeddings provide a chemically meaningful geometry that partially regularizes the optimization landscape while allowing candidates to be directly specified as discrete, human-interpretable design descriptions without training a task-specific model[67]. However, these embeddings are highdimensional (1532 dimensions in this work), which can make GP training computationally expensive and numerically ill-conditioned, particularly in the small-data regime typical of catalyst screening. To enable stable BO-GP optimization, we apply dimensionality reduction (Isomap to 32 dimensions), introducing additional modeling assumptions (e.g., preservation of local neighborhood structure) and hyperparameters such as neighborhood size and target dimensionality that can influence optimization performance[68].

BO-ICL instead uses the pretrained LLM directly as an implicit surrogate and operates on the original language representation without kernel specification, surrogate fitting, or dimensionality reduction. Candidate selection is guided through few-shot in-context examples, leveraging both the semantic structure encoded in the embedding space and the data-efficient generalization behavior of the LLM."

2. The authors should compare the performance of their proposed method with a direct approach where the LLM is used to recommend the next experimental sample, similar to the inverse design used for generating reference points in sub-pool construction. Such a comparison is essential to elucidate the specific role of BO within the BO-ICL.

Response:

The reviewer makes a good suggestion for benchmarking the BO-ICL algorithm. To run the experiment, we selected the following parameters for exclusive reliance on LLM inverse design: lambda = 1 (MMR), "greedy" acquisition function, and sub-pool size (k=1). These parameters lead to a cosine similarity ranking dependence for single-point sub-pool filling and assess performance with omission of the BO steps. The cosine similarity step is to help confine selection of the next evaluation point to accessible design parameters in the pool because the output experimental description from inverse design may not always match available points in the unlabeled pool, regardless of coercion from the system message. **Figure S11** exhibits behavior closely aligning with the analytical random trajectory. This result supports reliance on the BO steps associated with BO-ICL: MMR for sub-pool population, regression for mean and uncertainty estimates, and the acquisition function for ranking the next best evaluation points.

Action:

Experimental description and results have been added to section **S3.3** in the manuscript. Results are reproduced in the figure below, for the reviewer's convenience (**Figure S11**).

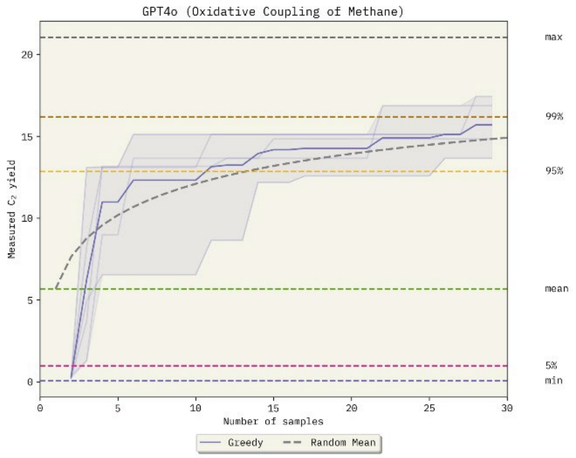

Figure S11: *BO-ICL performance on the OCM dataset with omission of the BO steps during inverse design. With the inversely inferred procedure that GPT-4o generates, we use the embedded representation as a reference in the cosine similarity algorithm to find the closest available procedure in the unlabeled pool to evaluate and add a new available context point. This experiment serves as a control to highlight the reliance on BO to achieve satisfactory performance.*

3. The authors should consider the potential biases introduced by the sub-pool construction, which involves a single round of inverse design and MMR pre-screening based on the result of inverse design to reduce token consumption. Relying on just one round of inverse design and the MMR method may introduce biases, potentially leading to the exclusion of samples with higher acquisition function scores.

Response:

Thank you for pointing out the potential bias introduced by the inference-sampling (IS) steps in BO-ICL, specifically the use of a single round of inverse design followed by MMR pre-screening for sub-pool population. We agree that relying on a single iteration of the IS step can produce a sub-pool that is not fully representative of the global design space and may exclude candidates with high acquisition values.

In this work, we chose a single IS call for the following reasons: **(1)** Introducing multiple inverse design and MMR rounds would add at least two additional hyperparameters (the number of IS rounds), for which there is currently limited theoretical guidance, potentially reducing reproducibility; **(2)** increased LLM calls and token usage; and **(3)** we observed robust empirical performance across five distinct datasets using a single IS implementation, suggesting that additional complexity is not required for the applications in our manuscript. At the same time, we acknowledge that in very large, highly heterogeneous, or multi-modal design spaces, increasing the number of IS rounds (and/or enlarging the

candidate pool prior to MMR) may help mitigate selection bias. We leave the choice to the user, which may be useful depending on the selected language model and design space.

Action:

To address the reviewer's concern, we have revised the **Limitations** section to indicate that our default single-iteration IS strategy may introduce selection bias during sub-pool construction, and clarified that the number of IS iterations as a tunable parameter trades off token cost against coverage of the design space. We have also added brief guidance on situations that may require increased IS rounds (*e.g.,* very large or heterogeneous design spaces). The excerpt from the **Limitations** section is below:

"*A related limitation arises from sub-pool construction. In the default BO-ICL implementation, we use a single inverse-design call followed by maximal marginal relevance (MMR) to populate a sub-pool and reduce token cost and inference latency. While efficient, this single-round inferencesampling (IS) step can, in principle, under-cover the global design space and exclude candidates with high acquisition values that lie outside the retrieved region, since BO is effectively performed over a filtered subset rather than the full pool. We adopt a single IS round to limit hyperparameter proliferation and improve reproducibility, as multiple inverse-design/MMR passes introduce additional user-defined settings (e.g., number of IS rounds and intermediate pool sizes) and increase LLM query cost. Empirically, we observe stable performance across datasets with a single IS round, suggesting this added complexity is unnecessary for the problem classes studied here. However, for very large, highly heterogeneous, or strongly multimodal design spaces, increasing the number of IS rounds and/or enlarging the candidate set prior to MMR may improve coverage and mitigate selection bias; thus, the number of IS iterations can be treated as a tunable parameter that trades off cost for design space coverage.*"

4. The authors should discuss how variations in language expression (e.g., "900°C" vs "900 Celsius" vs "reaction temperature set to 1173K") impact model performance. Additionally, they should clarify whether the text structure generated through inverse design aligns with the structure of data and explain how any inconsistencies could affect the retrieval or prediction processes.

Response:

Thank you for raising this important point regarding the sensitivity of language-based representations to variations in expressions and structure. We have performed the following analysis to understand how text-based changes and inconsistencies affect the resulting model performance:

(1) Effect of variations in language expression

We explicitly evaluated how superficial changes and chemically meaningful edits affect the embedding representations in BO-ICL. Using reference procedures from the trimetallic dataset (**Section S1.4.2, page 5**), we generated controlled variants spanning: Superficial perturbations (punctuation, spacing, formatting); unit-equivalent temperature rewrites (e.g., °C vs. K); additional temperature annotations (*e.g.,* appended parenthetical conversions); and chemically meaningful changes that will affect catalytic performance (*e.g.,* pre-treatment gas identity). Changes were quantified as the embedding distance between reference and variant text using cosine similarity.

We find that minor textual edits produce measurable embedding shifts above numerical noise. Chemically meaningful changes (*e.g.,* pre-treatment gas identity) consistently produce more significant representation changes than varied notation or formatting, indicating sensitivity to catalytically relevant information. However, the addition of new numeric or unit content (*e.g.,* appended temperature conversions) can induce embedding changes that are comparable to or larger than chemical edits. Thus, while the embeddings capture chemically relevant information, they also remain sensitive to superficial content, consistent with language pretraining intentions.

(2) Structure of inverse-designed text

Inverse design outputs are generated in the same procedural natural language format used in the training dataset, coerced by the context and system message (**Section S5**). Because BO-ICL operates directly on full-text representations rather than structured features, exact syntactic alignment is not required. However, sensitivity to numeric and formatting means that inconsistent expressions (*e.g.,* duplicated units, redundant annotations, or unusual phrasing) can influence embeddings and candidate rankings.

To mitigate these potential inconsistencies, prompts have been constructed with consistent formatting and are evaluated in the same representational space as the training data, ensuring that retrieval and scoring operate on comparable descriptions, even when phrasing varies. The reference procedure, with bold text highlighting the locations that were modified across variants, is as follows:

> *A trimetallic catalyst was synthesized by incipient-wetness co-impregnation onto CeO_2* ***,*** *with* ***5.0 wt %*** *total metal loading and* ***0.5 wt %*** *potassium promoter.* ***The*** *active metals were Zn, Mo, and W in a molar ratio* ***of*** *2.1:1.0:1.5. Due to differing pH requirements, sequential impregnation was employed. Initially, acidic-stable precursors were dissolved in HNO_3-acidified Milli-Q water (pH 1.8), impregnated onto the support, dried at 90 °C for 4 h, and calcined at 450 °C. Subsequently, a solution containing ammonium molybdate (para) tetrahydrate and ammonium metatungstate*

hydrate was used in a second impregnation step ***(~pH 4)****, followed by drying and final calcination. Prior to testing, the catalyst was reduced in* ***CO*** *at 600* ***°C*** *under 50 psig**, at** 20 mL/min. Testing was performed at 300 °C using a gas mixture of* CO_2 ***(22.2 %),*** H_2 ***(66.7 %), and*** *Ar* ***(11.1 %)*** *at 45 mL/min and 1 atm.* ***The*** *sample loading was 75 mg, corresponding to* ***a GHSV of 54,000*** *h^{-1}.*

Overall, the embedding space reflects both chemical content and textual formatting. Chemically consequential edits produce larger representation shifts than purely cosmetic formatting changes, but numeric and unit annotations can also strongly influence the embeddings. These findings support our use of controlled textual formats when interpreting BO-ICL predictions and motivates maintaining consistent procedural structure in inverse designed candidates.

Action:

We have added the following additional analysis and figure to the Supporting Information:

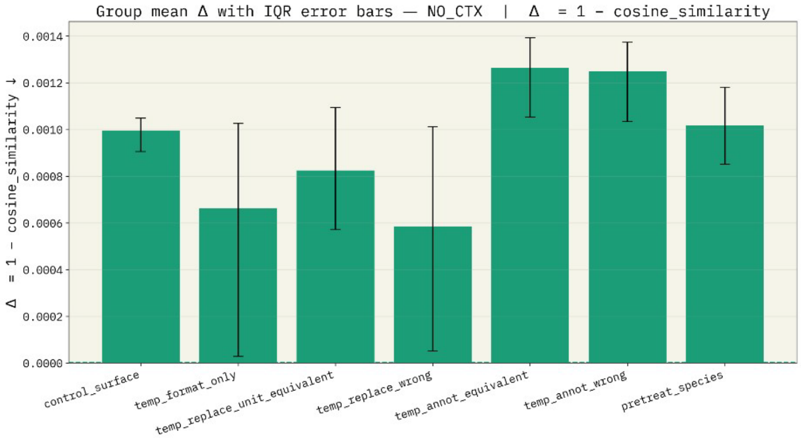

Figure S17: Mean embedding distance by perturbation class under the no-context condition (text-embeddingada-002). Bars show the mean semantic distance Δ = 1 − cosine_similarity (e_ref, e_var) between the referenc
catalytic procedure and single-parameter variants grouped by edit type. Error bars indicate the interquartile range (25th--75th *percentile) across variants within each group. Larger Δ indicates greater deviation from the reference embedding. Temperature annotations and unit-equivalent substitutions produce the largest representation shifts, while superficial edits and format-only changes produce smaller but measurable effects. Chemically meaningful pretreatment gas substitutions yield intermediate-to-large shifts, indicating sensitivity to catalytically relevant parameters.*

We have also added the following text to **Section S7 (page 26)**:

"*We have explicitly evaluated how superficial changes and chemically meaningful edits affect the embedding representations used in BO-ICL. We hypothesize that changes in embedding feature vectors correlate strongly with inference variance;*

therefore, measuring the cosine similarity between a reference procedure and a counterfactual should reveal the relative importance of specific parameters, which may guide future procedure structure selection. Using reference catalytic procedures from the trimetallic dataset (Reference example S1), we generated controlled single-parameter variants spanning: ***(i)*** *superficial perturbations (punctuation, spacing, formatting),* ***(ii)*** *unit-equivalent temperature rewrites (e.g., °C vs K),* ***(iii)*** *additional temperature annotations (e.g., appended parenthetical conversions), and* ***(iv)*** *chemically meaningful changes that affect catalytic performance (e.g., pre-treatment gas identity).*

Changes were quantified by the embedding distance between reference and variant text using cosine similarity. We find that even minor edits produce measurable embedding shifts above numerical noise. Chemically meaningful changes (e.g., pre-treatment gas identity) consistently produce larger representation changes than purely notational or formatting variations, indicating sensitivity to catalytically relevant information. However, adding new numeric or unit content (e.g., appending temperature conversions) can induce embedding changes comparable to, or larger than, chemistry edits. Thus, while the embeddings capture chemically relevant distinctions, they also remain sensitive to form and numeric content, consistent with their general languagepretraining objective.

*Inverse-design outputs are generated in the same procedural natural language format used in the training dataset, guided by the context and system message (****Section S5****). Because BO-ICL operates directly on full-text representations rather than structured features, exact syntactic alignment is not required. However, sensitivity to numeric and formatting content means that inconsistent expression (e.g., duplicated units, redundant annotations, or unusual phrasing) can influence embedding geometry and therefore candidate ranking. To mitigate this, prompts are constructed to encourage consistent procedural formatting, and candidate texts are evaluated in the same representational space as the training data. This helps ensure that retrieval and scoring operate on structurally comparable descriptions, even when phrasing varies.*

Across the single-parameter variants we tested superficial edits (punctuation/spacing) and alternate temperature spellings (e.g., "300 °C" vs "300 Celsius"), which produce small, but measurable changes above noise. Chemically meaningful substitutions, such as changing the pretreatment gas (CO to H_2 *or Ar), consistently induce shifts that are typically larger than temperature formatting. However, embedding distance is driven most strongly by added numeric content, for example, appending an equivalent temperature conversion (e.g., "300 °C (573 K)") produces the largest representation shift.*

Because BO-ICL performs retrieval and candidate ranking directly in the embedding space, these expression-driven shifts can change which procedures are retrieved and how candidates are ordered, even when the intended physical conditions are equivalent. To keep inverse-designed outputs comparable to the training data, we use the same format and parameter ordering as the corpus, and prompts are designed with consistent formatting. When candidates include inconsistent or redundant expressions (e.g., duplicated units, repeated conversions, or atypical phrasing), their embeddings can bias retrieval and scoring, motivating controlled textual templates for both interpretation and inverse design."

5. What is the difference between the "completion model" and the "chat model" in Figure 5? According to the text, both appear to be based on GPT-4o, with only the acquisition function differing.

Response:

Thank you for pointing out this ambiguity. The key difference is the post-training alignment applied to the underlying GPT base model: a "completion model" does not undergo reinforcement learning from human feedback (RLHF), whereas a "chat model" is a completion model that has been further aligned with RLHF to produce conversation-style responses. As a result, during the inverse design step of BO-ICL we used a system message with the chat model to control the response style, since this additional alignment can introduce unwanted variations.

Action:

In **Section 2.2.2 (page 9)**, we have described the key differences between the "completion model" and the "chat model" and how they affect BO-ICL:

"*Using the AII dataset, we further examine whether different LLMs are better suited to certain inference steps within the BO-ICL workflow. For all other datasets, we use gpt-4o-2024-08-06 at every inference step (Figure 4). In contrast, for the property-value prediction and uncertaintyestimation step (workflow step A7), we use the davinci-002 base completion model. Empirically, davinci-002 produces better-calibrated uncertainty estimates on AII, indicating that predicted uncertainties more closely track observed prediction errors than gpt-4o-2024-08-06 (Figure 5, center). One possible explanation is that instruction-tuned models optimized with reinforcement learning from human feedback (RLHF) may trade off probabilistic calibration for human preferences, which can be disadvantageous when accurate uncertainty quantification is required.*

Our decision to incorporate davinci-002 comes from the observed importance of model calibration on overall performance (see Section 5). Using a well-calibrated off-the-shelf model for the regression step alleviates the need for post-training calibration and reduces the number of initially labeled data points required to achieve satisfactory performance. For the inverse-design generation step (workflow step O1 in Section 5.2), we use gpt-4o-2024-08-06, as its RLHF training encourages an output structure that more closely aligns with the natural-language format of the experimental procedures. This alignment is particularly useful for the similarity comparison and retrieval steps in the optimization loop (workflow steps A2–O3, and Section 2.2.3). The performance differences when using a single model (gpt-4o-2024-08-06) versus a combination of a base model and a chat model (davinci-002 and gpt-4o-2024-08-06) in the workflow may further highlight the critical role of accurate uncertainty estimation when comparing upper confidence bound (UCB) trajectories (see Section 5.1 for acquisition function details)."

6. The authors should carefully review and revise the manuscript to address basic errors throughout the text, e.g.,

Response:

Thank you for the valuable comment and careful attention to the formatting of the manuscript. We have addressed all comments, as indicated below.

Actions:

a) The abstract should specify the full form of "ICL" for clarity, as it is used without definition. Addressed.

b) The abbreviation "KRR" is inconsistently capitalized throughout the manuscript, for example, lines 13, 34, 38, and 54 on page 4. Addressed.

c) What do the shaded areas represent in Figures 4 and 5? And, in Figure 4, what do the darker lines (but not the darkest) within the shaded area represent for UCB and Greedy?

Addressed in the figure captions in the manuscript. The lighter lines are the replicate trajectories: 5 replicates per BO run. The darker line is the average of these replicate trajectories. The shaded areas represent the range of the outputs, highlighting the variance of the BO process.

d) The "upper confidence bound" in the legend of Figure 5 should be formatted consistently with Figure 4, using the abbreviation instead of the full form. Addressed.

e) On page 14, line 19, Algorithm 2 is mentioned, but it does not appear in the main text. Addressed.

f) On page 14, line 10, the manuscript references the pseudo-code for the BO-ICL implementation (Algorithm 1), but the actual Algorithm 1 shown is the "Bayesian Optimization Policy for Reaction Runs." Addressed.

g) What is the full form of "MMR" mentioned on page 14 and “RAG” mentioned on page 4. Addressed.

h) In line 3 of page 14, the "T" in "Total" should be lowercase. Addressed.

i) There is an inconsistency between the order of subfigures in Figure 7 and the description in the caption. Additionally, the caption mentions the catalyst formulation K@ZnZrMo(8.0:1.0:2.0)/TiO2, but this is not shown in the figure. Meanwhile, the K@ZnMoMn(2.1:3.0:1.5)/CeO2 formulation is introduced in the figure but is not discussed in the text. Addressed.

Reviewer 2:

This manuscript introduces BO-ICL, a framework that employs large language models (LLMs) as surrogate models for Bayesian optimization (BO) in catalyst discovery. By leveraging in-context learning (ICL), the authors demonstrate that frozen LLMs can directly map natural language descriptions of experimental

procedures to catalytic performance, effectively bypassing traditional feature engineering and explicit model training. This work is innovative and compellingly bridges generative AI with experimental automation. This manuscript is suitable for publication in ACS Central Science after minor revisions. Below are some comments.

Response:

Thank you for your favorable opinion of our manuscript. We have fully addressed all of your insightful comments in our response below.

1. The authors first use an LLM-based inverse design to generate an ideal procedure (x'), followed by MMR-based sub-sampling from the candidate pool U. The rationale for not using the LLM to directly recommend N ideal procedures should be clarified. Furthermore, the validity of this virtual x' lacks benchmarking. If the generated x' is strongly biased by the model's prior, subsequent MMR retrieval may be confined to a potentially erroneous subspace.

Response:

Thank you for pointing out the potential bias in our work. This comment is very similar to that of Reviewer #1, comments #2 and #3, which we have addressed above. A few additional comments are here for the reviewer's convenience:

(1) Not using LLM directly to populate the subpool

Avoiding reliance on N inverse design procedures to populate the subpool offers two primary advantages: **(1)** Increased robustness against model hallucinations. By using a procedure from inverse design as the reference for MMR to populate the sub-pool, we ensure that the populated space includes procedures that exist in the unlabeled pool and balance model exploration; and **(2)** we avoid bias because if we were to rely on the model's N inverse design procedures given the same context, the result would be N similar procedures and less exploration of the experimental space.

(2) Benchmarking BO

The reviewer makes a good suggestion for benchmarking the BO-ICL algorithm. To run the experiment, we select the following parameters for exclusive reliance on LLM inverse design: lambda = 1 (MMR), "greedy" acquisition function, and sub-pool size (k=1). These parameters lead to a cosine similarity ranking dependence for single-point sub-pool filling and assess performance with omission of the BO steps. The cosine similarity step is to help confine the selection of the next

evaluation point to accessible design parameters in the pool because the output experimental description from inverse design may not always match available points in the unlabeled pool, regardless of coercion from the system message. **Figure S11** (see response to Reviewer #1, Comment #2) exhibits behavior closely aligned with the analytical random trajectory. This result supports reliance on the BO steps associated with BO-ICL: MMR for sub-pool population, regression for mean and uncertainty estimates, and the acquisition function for ranking the next best evaluation points.

Action:
Rationale on the reason for avoiding exclusive reliance on the LLM for sub-pool suggestion and population has been added to the methods section (**Section S3**). This control experiment and its results have been added to **Section S3.3** of the Supporting Information. The results are also reproduced in **Figure S11** (see response to Reviewer #1, Comment #2).

2. The manuscript applies a fixed uncertainty scaling factor (e.g., 5), which is optimized on the OCM dataset, directly to other tasks such as RWGS. The authors should provide a justification for why a scalar parameter tuned on one task exhibits such strong cross-domain transferability.

Response:
We agree that using a fixed uncertainty scaling factor (*e.g.,* 5), tuned on OCM and then applying the same scaling factor to RWGS requires justification. It is important to clarify that we did not assume cross-domain transferability *a priori*. The fact that the same scaling performed well across tasks was an empirical observation and should not be interpreted as a guaranteed property of the method.

A plausible explanation is that this scalar compensates for systematic miscalibration in the LLM's predictive uncertainty rather than encoding domain-specific structure. In our setting, the surrogate's uncertainty estimates can be mis-scaled due to limited context, heteroscedastic noise, and model-form mismatch. A single multiplicative factor can therefore act as a global calibration "temperature" on uncertainty. If the dominant issue is "uncertainty amplitude" rather than relative ranking, then many reasonable rescalings can improve acquisition behavior across domains.

Our interpretation is consistent with standard Bayesian optimization practice: scaling plays a role analogous to the exploration coefficient in UCB-style acquisition functions (*e.g.,* β) that rescales the uncertainty term to set the exploration-exploitation tradeoff. Therefore, the value of "5" is not special to OCM, it is one instance of a tuning knob for controlling exploration when uncertainty is imperfectly calibrated.

Action:

We expanded the **Limitations** section to include the following: **(i)** clarifications that cross-task transfer is empirical and not guaranteed; **(ii)** explanations of the calibration/temperature interpretation; and **(iii)** recommendations for re-tuning the scaling factor when transferring to substantially different tasks. The revised text is included below for the reviewer's convenience:

"Uncertainty calibration introduces additional limitations. For global optimization, BO relies on relatively accurate uncertainty estimates to guide exploration[28, 34]. However, modern LLMs, particularly those aligned by RLHF, can exhibit miscalibrated confidence, complicating uncertainty estimation without access to substantial validation data [76, 80] (Section 5.3). This requirement partially conflicts with BO's primary advantage of optimizing objectives with minimal data. In this work, we mitigate calibration challenges by (i) using base models that empirically yield more stable uncertainty behavior in our setting, and (ii) leveraging transfer when a calibration mapping can be learned from a related dataset. For example, as described in Section 5, we derive an uncertainty scaling factor using a validation subset from the OCM dataset (via Uncertainty Toolbox) and find that this scaling improves BO-ICL behavior beyond OCM (e.g., AII,ESOL. and RWGS). While calibration is often treated as domain- and dataset-specific, these results suggest that cross-domain calibration transfer can be a practical compromise when only limited validation data are available."

3. As the BO loop progresses, the labeled dataset L (and thus the memory M) increases in both size and quality. The authors should discuss whether keeping a constant k value remains optimal throughout the process.

Response:

We agree that as the BO loop progresses, the labeled set L (and thus the memory M) increases in both size and quality, so a fixed retrieval count k is not guaranteed to remain optimal throughout the process. In this work, we kept k fixed for three pragmatic reasons. First, our sensitivity analysis (**Figure 9**) indicates that performance is not strongly improved by changing k in the regimes we tested (adapted OCM and ESOL; N = 10 and N = 1000 where N = unlabeled pool size). In particular, we selected k = 5 because increasing or decreasing k did not yield consistent gains (*e.g.,* opposing trends in correlation and MSE for regression tasks), suggesting a trade-off between additional context and diluted relevance.

Second, bounding k is consistent with prior in-context learning findings: beyond a modest number of demonstrations, returns may saturate or degrade due to attention dilution and contextwindow constraints[2]. This makes a constant, small k a robust default across tasks and models.

Third, while k is fixed, the retrieved set is not. At each BO iteration, the k examples included in the prompt are dynamically re-selected from the current memory (M) based on relevance to the current query. As M grows and improves, retrieval quality improves accordingly, so the method benefits from the increasing

quality of M even without increasing k. In other words, we leverage growth in M primarily through better examples rather than more examples.

That said, we agree with the reviewer that adaptive strategies for k (*e.g.,* increasing k as M expands, or selecting k based on retrieval confidence, uncertainty, or diversity) are a natural extension of our work.

Action:

We now explicitly discuss the prospect of treating k as a tunable (and potentially adaptive) hyperparameter in the **Limitations** section, and we clarify that k is fixed, but retrieval is dynamic across BO iterations (corresponding excerpt below):

"To reduce this failure mode, we seed BO-ICL with diverse initial experiments. After selecting an initial reference point, we compute cosine similarity between its embedding and all remaining candidates and select the most dissimilar point (a farthest-point heuristic) to construct initial context with maximum diversity. In our experiments, we use k = 2, but this approach generalizes to larger k by iteratively selecting the candidate with the largest minimum distance to the running labeled set. When larger labeled datasets are available, practitioners should explicitly balance allocating labeled data to seed a diverse BO-ICL context set versus reserving labeled data for supervised fine-tuning or calibration, since both choices can materially affect exploration behavior and sample efficiency. While this initialization strategy improves coverage in practice, it does not guarantee global exploration, particularly in very large or strongly multimodal design spaces.

As the BO loop progresses, the labeled set L (and memory M) grows, so a fixed retrieval count k is not guaranteed to remain optimal. Nevertheless, we keep k fixed as a pragmatic design choice for three reasons: ***(i)*** *Sensitivity analysis (Figure 9) shows that varying k in the tested regimes yields no consistent improvement, suggesting a trade-off between adding helpful demonstrations and diluting relevance under a finite context budget;* ***(ii)*** *keeping k modest is consistent with prior findings in in-context learning that gains can saturate or degrade as the number of demonstrations increases [65]; and* ***(iii)****k is fixed, but retrieval is dynamic: at each iteration, the k demonstrations are re-selected from the current M based on relevance, so the method benefits from improved memory quality primarily through better example selection rather than more examples. We view adaptive k schedules (e.g., increasing k with |M| or conditioning on retrieval confidence or diversity) as a natural extension of this work."*

4. Sub-sampling is employed across all datasets to manage API latency and cost. If feasible, the authors should provide a benchmark on a smaller dataset comparing sub-sampling against a full global predictive search.

Response:

Thank you for suggesting this benchmark. We agree it would be an informative baseline. When the candidate pool is small enough that API latency and inference

cost are not limiting, users should avoid sub-sampling and instead evaluate the full candidate set directly, since this eliminates sampling variance and provides the most faithful assessment of BO performance.

We did not include a full global predictive-search baseline in this study primarily because our focus is on large candidate spaces (large N) where exhaustive evaluation is impractical and expensive. This trade-off was also reflected in our surrogate experiments: fine-tuning improved regression accuracy (*e.g.,* **Figure 2** parity plots), but at increased training and/or inference cost, suggesting that more expensive modeling choices (including exhaustive candidate evaluation) are more practical when the candidate space is sufficiently small and/or when ample initial labeled data are available.

Action:

We added a statement to the **Limitations** section recommending that, when feasible, practitioners prioritize evaluation over the true candidate space (rather than a controlled sub-sampled space), while explicitly weighing the associated latency, monetary cost, and environmental implications of large-scale inference. The excerpt is below:

"Sub-sampling is used across datasets to reduce API latency and inference cost, but it introduces sampling variance and can obscure the true behavior of the optimization policy. When the candidate pool is small enough that cost and latency are not limiting, practitioners should avoid sub-sampling and instead evaluate the full candidate set directly, since exhaustive scoring eliminates sampling variance and provides the most faithful assessment of BO performance. We did not include a full global predictive-search baseline in this study because our focus is the regime where BO is typically most useful, large candidate spaces where exhaustive evaluation is impractical, and because full-space LLM querying can be expensive and energy-intensive at scale. More generally, the most appropriate choice depends on the candidate-space size and resource constraints. Full evaluation is preferable when feasible, while sub-sampling is a practical compromise when throughput, monetary cost, or environmental considerations dominate."

5. In Table S4, a parameter configuration is listed as N = 1 with k = 5. Mathematically, if the total number of observations N is 1, it is impossible to retrieve k = 5 distinct and relevant examples for the prompt context. The authors should clarify the definitions of N and k in this setting or correct any potential labeling errors to ensure the integrity of the supplementary results.

Response:

Thank you for pointing out that additional clarifications are needed to explain Table S4. To clarify the definitions of N and k: N denotes the size of the available retrieval pool, while k is the **maximum** number of examples used for the prompt. When

N<5, the model simply includes all available samples rather than enforcing exactly k=5. We have clarified this in the Supporting Information to avoid confusion.

Action:
We have added some detail to **Section 5.2** to address the reviewer's comment:

"*The {few-shot template} formats the context by concatenating k examples using the following structure: "Given {representation}. What is {property_name}? {completion}". Figure 1 illustrates how the prompt is constructed when selecting k = 1 example as context. In all BO-ICL experiments presented, we use k = 5; if the available context count is less than k, all available context is included in the prompt. Finally, the {suffix} contains the primary query of interest for which the LLM should provide a completion.*"

6. The mathematical expression for MMR in Figure 8 appears to contain an error. A thorough check of the manuscript is recommended to ensure consistent and accurate notation throughout.

Response:
Thank you for pointing out our error in the flow chart in term 2 (**Figure 8**) of the MMR equation, where the query should not be involved in the quantitative pool diversity check.

Action:
We have corrected the expression to match the correct formulation as shown in equation 6 of the manuscript (**Figure 8**).

Editor Comments/Formatting Needs:

Author Affiliations: Please include postal codes/country in the author affiliations in the publication file(s). Addressed.

Major Objects: Please cite each major object (figures, tables, equations and schemes) in the manuscript text. If a figure is not cited as a whole, at least one part must be cited (e.g. Figure 1 or Figure 1a). Equations 1, 4 and 7 are not cited. Addressed.

References: Please make sure that the number of references in the Reference list matches the number of references cited in the manuscript text. References 92 to 97 do not appear to be cited in the manuscript. Addressed. These citations were referred to in the SI and have now been assigned in the revised SI references.

Supporting Information: If the manuscript is accompanied by any Supporting Information for Publication, a brief description of the supplementary material is required in the manuscript, before the reference list. The appropriate format is: Supporting Information. Brief statement in nonsentence format listing the contents of the material supplied as Supporting Information. Please list each supporting item individually. Addressed.

Supporting Information: Please number all pages in the following format: S1, S2, S3, etc. Addressed.

References:

1. Su, Y.; Wang, X.; Ye, Y.; Xie, Y.; Xu, Y.; Jiang, Y.; Wang, C. Automation and Machine Learning Augmented by Large Language Models in a Catalysis Study. Chem. Sci. **2024**, 15 (31), 12200–12233.

2. Many-Shot In-Context Learning. https://arxiv.org/html/2404.11018v3.

oc-2025-02418s.R2

Name: Peer Review Information for "Bayesian optimization of catalysis with in-context learning"

Second Round of Reviewer Comments

Reviewer: 1

Comments to the Author

The authors have comprehensively and diligently addressed mostly the reviewers' comments and suggestions. Particularly, the inclusion of evidence supporting their claims is helpful. The remaining points requiring revision are as follows:

1. The authors have demonstrated that the performance obtained by relying entirely on LLM inverse design is highly consistent with that of analytical random trajectory (Figure S10 in SI). Based on this conclusion, can we further argue extremely that the inverse-design based strategy is in fact redundant during subpool construction? In other words, replacing

the inverse design results with single random samples for subpool construction, followed by BO, may also achieve performance comparable to existing methods.

2. I thank the authors for the clarification regarding the completion model and chat model. In the in-house RWGS case, the legend of Figure 6 indicates that the greedy strategy (green line) uses the completion model, while the UCB strategy uses the chat model. However, both the figure caption and the main text only mention gpt-4o-2024-08-06 and do not specify the use of the completion model (davinci 002). Where is the typo?

3. There remains an issue with the left panel of Figure 7. The title shows K@ZnMnMo(2.1:1.5:1.0)/CeO_2, but this catalyst is not mentioned anywhere in the main text or the figure caption.

4. The abbreviation krr appears in lowercase on Page 4, Line 24 and Page 4, Line 44, which is inconsistent with the uppercase notation used elsewhere. A thorough check of the manuscript is recommended to ensure consistent and accurate notation throughout.

Author's Response to Peer Review Comments:

Response to Reviewer Comments for:
Bayesian optimization of catalysis with in-context learning

Reviewer 1:

1. The authors have demonstrated that the performance obtained by relying entirely on LLM inverse design is highly consistent with that of analytical random trajectory (Figure S10 in SI). Based on this conclusion, can we further argue extremely that the inverse-design based strategy is in fact redundant during subpool construction? In other words, replacing the inverse design results with single random samples for subpool construction, followed by BO, may also achieve performance comparable to existing methods.

Response:

We thank the reviewer for raising this important point that requires clarification. Our results do not support the conclusion that using the inverse-design algorithm for subpool population is redundant. In early ablation experiments using davinci-003, we explicitly tested fully random (“all_random”) and partially random (“half_random”) strategies for subpool population alongside the current strategy (“no_random”) in **Figure R1**. As shown in the figure, both strategies involving randomly selected points in the subpool tended to perform worse with respect to the desired

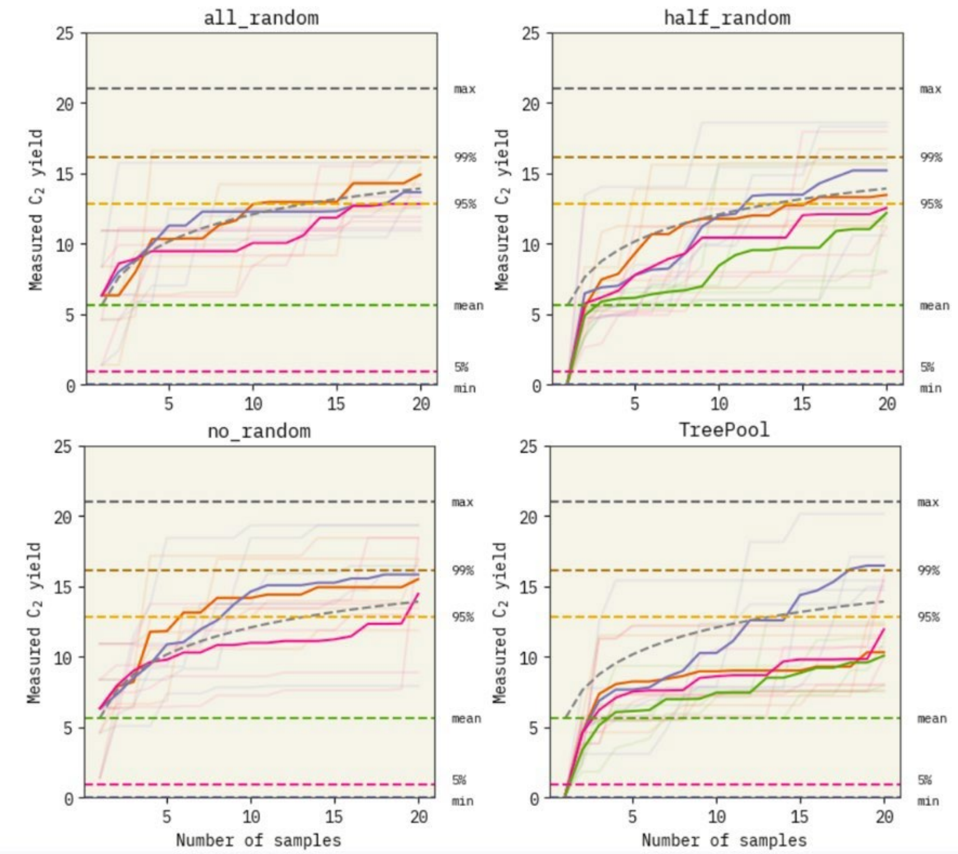

Figure R1: *Early ablation tests using different methods for subpool population using the OCM dataset. (acquisition functions: orange: Expected Improvement, purple: Greedy, pink: UCB w/ modified exploration parameter). Empirically, the data implies a negative correlation with randomly selected subpool population.*

objective. This suggests a measurable impact on BO performance, although we acknowledge that the statistical significance of this effect remains uncertain.

We therefore interpret **Figure S10** more narrowly. Specifically, the figure shows that pure reliance on the model's bias can in some cases resemble a random walk, but this does not imply that inverse-design-guided subpool construction is unnecessary for the BO-ICL algorithm to perform well. Rather, the empirical results suggest that model-guided subpool construction improves the quality of the candidate set presented to the BO pipeline relative to relying on random sampling to approximate the population space before applying BO.

A likely reason is that a fully random subpool reduces our control over coverage of the design space, especially in early iterations, and may populate the candidate set with points that are redundant or insufficiently informative. In contrast, inverse-design-guided construction introduces model bias toward promising design regions, while BO hedges against overreliance on that bias by still providing an exploration-based correction.

One example of potentially undesired bias that may benefit from such hedging is our analysis of model representation priorities in comparisons between language-level prompt features and catalysis-relevant properties. This analysis suggests that the model may sometimes rely more strongly on semantic or surface-form cues than on fine-grained numerical variables. As a result, the model may correctly identify broader catalyst combinations or pretreatment species while remaining less sensitive to small changes in parameters such as temperature. This is a meaningful

limitation in catalysis, because even small numerical changes can produce significant differences in chemical behavior. Searching in the local region of an inversely proposed procedure may therefore help offset this bias by refining the model's initial suggestion within a chemically relevant neighborhood. More broadly, pretraining does not imply that the model's first proposed design is itself optimal, but rather that it may identify a point near a promising region of design space. Therefore, BO-ICL combined with inverse-design, may provide a way to exploit the useful directional bias of the model while correcting for limitations.

Action:

We have revised the manuscript to clarify that **Figure S10** does not demonstrate redundancy of inverse design, and to discuss the ablation results comparing random and non-random subpool construction more explicitly. We have also added ablation results referenced in the is response document to the SI **(Figure R1**/**Figure S11**). These additions are included below:

> "*In this context, the result obtained using inverse design alone (**Figure S10**) should be interpreted narrowly: a trajectory based entirely on inverse design can in some cases resemble a random walk, but this does not imply that inverse-design-guided subpool construction is uninformative. Rather, it suggests that inverse design alone may be insufficient to fully control exploration. One plausible interpretation is that inverse design contributes useful directional bias toward promising regions, while BO refines and corrects those proposals through local search. This may be especially relevant in catalysis settings, where the model can capture broad semantic relationships among catalyst compositions and processing conditions, yet remain less sensitive to small numerical variations that can strongly affect chemical behavior.*
>
> *Together, these figures suggest that subpool construction strategy materially affects downstream BO performance. In early ablation experiments with davinci-003 on the OCM dataset, fully random (all_random) and partially random (half_random) subpool population both tended to underperform relative to the structured (no_random) strategy (**Figure S11**). This trend suggests that increasing reliance on random subpool population may reduce the quality of the candidate set presented to BO, particularly in early iterations, when coverage and diversity are most important.*"

2. I thank the authors for the clarification regarding the completion model and chat model. In the in-house RWGS case, the legend of Figure 6 indicates that the greedy strategy (green line) uses the completion model, while the UCB strategy uses the chat model. However, both the figure caption and the main text only mention gpt-4o-2024-08-06 and do not specify the use of the completion model (davinci 002). Where is the typo?

Response:

This is an important distinction, thank you. The completion model here was *gpt-4 (completion model)*, not *gpt-4o (chat model)*.

Action:

The figure caption has been updated as shown below, and corresponding references elsewhere in the manuscript have also been updated:

> *"**Figure 6**: BO-ICL results on a pool of RWGS experiments. Purple: six randomly selected experiments. Green: BO-ICL with gpt-4 using a greedy acquisition function. Orange: BOICL with gpt-4o-2024-08-06 using UCB; stars indicate the surrogate mean prediction prior to execution and error bars indicate model uncertainty ($\mu \pm \sigma$). The dashed line denotes the equilibrium CO yield computed for the inlet composition and reactor temperature (Eq. S5)."*

3. There remains an issue with the left panel of Figure 7. The title shows K@ZnMnMo(2.1:1.5:1.0)/CeO_2, but this catalyst is not mentioned anywhere in the main text or the figure caption.

Response and action:

Thank you for this careful observation. It is important that the figures are clearly supported by the main text and that the information they present is not left unexplained. We have added the catalyst to the manuscript here for further clarity:

> *"Although alkali promotion (K), reducible oxides (e.g. CeO_2, ZrO_2), and transition metal carbides are often associated with CO$_2$ activation, these factors alone do not explain the catalyst performance, as closely related compositions such as K@ZnZrMo(2.1:1.5:1.0)/CeO_2, compared in **Figure** 7, are significantly less active."*

4. The abbreviation krr appears in lowercase on Page 4, Line 24 and Page 4, Line 44, which is inconsistent with the uppercase notation used elsewhere. A thorough check of the manuscript is recommended to ensure consistent and accurate notation throughout.

Response and action:

We thank the reviewer for highlighting this inconsistency. The manuscript has been updated to correct the notation. We also reviewed the manuscript for similar issues and did not identify any additional inconsistencies.
